# Supplementary material for: Exon junction complex dependent mRNA localization is linked to centrosome organization during ciliogenesis
Source: Nat Commun. 2021 Mar 1;12:1351. doi: 10.1038/s41467-021-21590-w (PMC7921557; doi:10.1038/s41467-021-21590-w)
Supplement: Supplementary file 1 — Supplementary Information [file 41467_2021_21590_MOESM1_ESM.pdf]

Supplementary information

Exon Junction Complex dependent mRNA localization is linked to centrosome organization during ciliogenesis

Oh Sung Kwon[1], Rahul Mishra[1,#], Adham Safieddine[2,3], Emeline Coleno[2,3], Quentin Alasseur[1], Marion Faucourt[1], Isabelle Barbosa[1], Edouard Bertrand[2,3], Nathalie Spassky[1] and Hervé Le Hir[1,*]

[1]Institut de Biologie de l'Ecole Normale Supérieure (IBENS), Ecole Normale Supérieure, CNRS, INSERM, PSL Research University, 46 rue d'Ulm, 75005, Paris, France

[2]Institut de Génétique Moléculaire de Montpellier, University of Montpellier, CNRS, Montpellier, France

[3]Equipe labélisée Ligue Nationale Contre le Cancer, University of Montpellier, CNRS, Montpellier, France

[#]Present address: Institute of Parasitology, Biology Centre, Czech Academy of Sciences, Ceske Budejovice, Czech Republic

*To whom correspondence should be addressed. Tel: +33 1 44 32 39 45; Email: lehir@ens.fr

This file includes:

Supplementary Figures 1 to 9

Supplementary Tables 1

Fig.S1

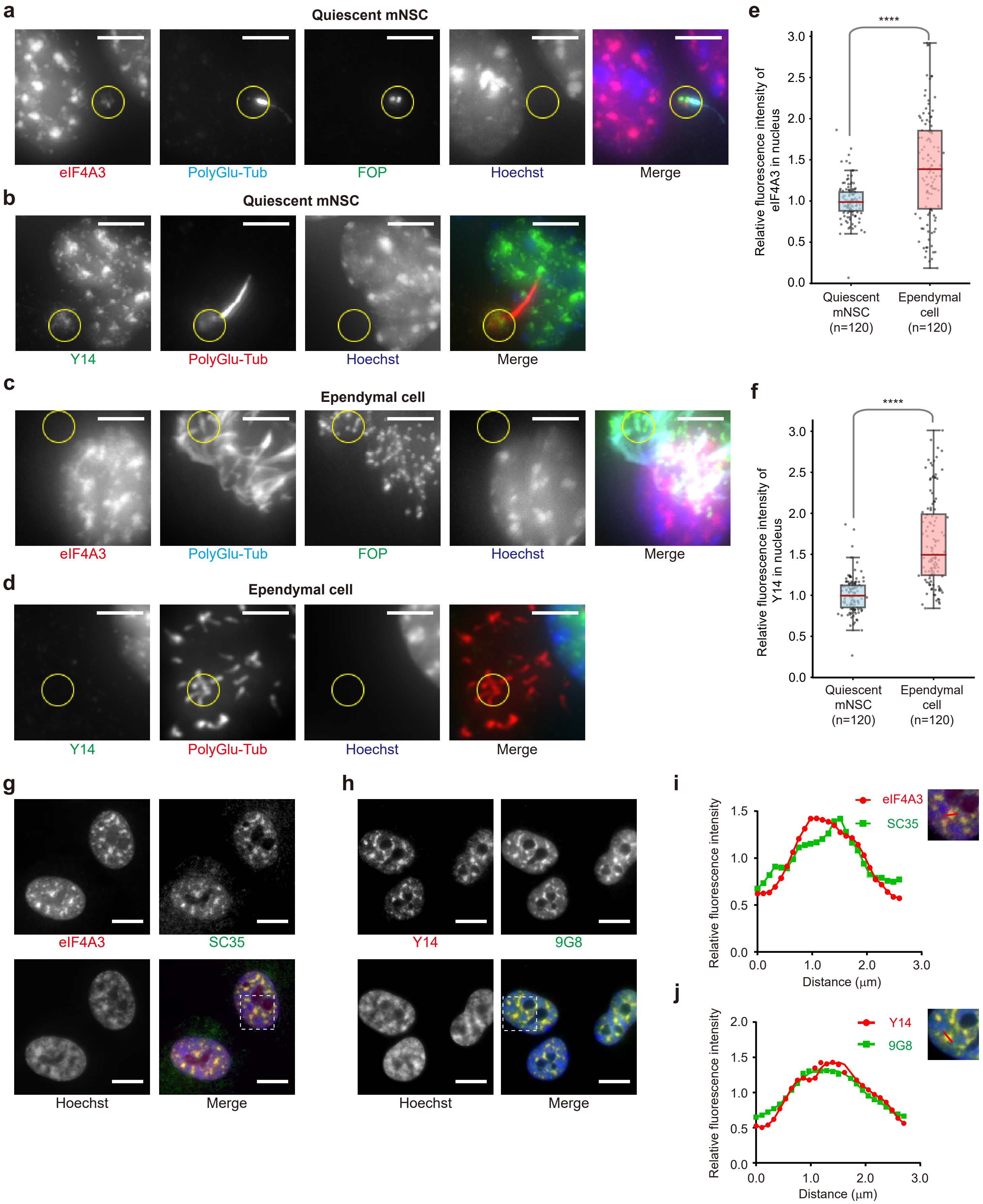
a
Quiescent mNSC
eIF4A3
PolyGlu-Tub
FOP
Hoechst
Merge
b
Quiescent mNSC
Y14
PolyGlu-Tub
Hoechst
Merge
c
Ependymal cell
eIF4A3
PolyGlu-Tub
FOP
Hoechst
Merge
d
Ependymal cell
Y14
PolyGlu-Tub
Hoechst
Merge
e
****
Relative fluorescence intensity of eIF4A3 in nucleus
Quiescent mNSC (n=120)
Ependymal cell (n=120)
f
****
Relative fluorescence intensity of Y14 in nucleus
Quiescent mNSC (n=120)
Ependymal cell (n=120)
g
eIF4A3
SC35
Hoechst
Merge
h
Y14
9G8
Hoechst
Merge
i
eIF4A3
SC35
Relative fluorescence intensity
Distance (μm)
j
Y14
9G8
Relative fluorescence intensity
Distance (μm)

Figure S1. Fluorescence intensity measurement of EJC proteins around centrosomes, increase of eIF4A3 and Y14 upon differentiation and their colocalization with nuclear speckle markers.

Quiescent mNSC (a, c) and multiciliated ependymal (b, d) cells were stained for eIF4A3 (a, b) or Y14 (c, d). Centrosomes were labeled by FOP antibody and primary cilia and centrioles were stained by poly-glutamylated tubulin (PolyGlu-Tub) antibody. Nuclei were stained by Hoechst. Images result from maximum intensity projections of 12 z-stacks acquired at every 0.5 μm. Fluorescence intensity of eIF4A3 (a) and Y14 (c) were determined in a 2 μm circle around centrosomes and base of cilia. In multiciliated ependymal cells, 2 μm circles were manually selected at the base of multicilia, and fluorescence intensities of eIF4A3 (b) and Y14 (d) were determined. Scale bars are 3 μm. Relative fluorescence intensities of eIF4A3 (Fig.1a, b) and Y14 (Fig. 1c, d) in the nucleus were determined in Hoechst stained area and plotted in panel e and f as described in legend of Figure 1. Boxes represent values between the 25th lower and 75th higher percentile, and the red lines mark the median. Whiskers above and below correspond to 0.35th lower and 99.65th higher percentile, respectively. **** $P \leq 0.0001$, two-tailed Mann-Whitney test. The number of cells analyzed in three independent experiments is provided (e, f). RPE1 cells were stained with eIF4A3 and SC35 (g) and Y14 and 9G8 (h) antibodies. Scale bars represent 10 μm (g, h). Images from white dashed squares from g and h are shown in i and j, respectively. Relative fluorescence intensity profile of eIF4A3 with SC35 (i) and Y14 with 9G8 (j) along the red line on nuclear speckle were plotted. Average fluorescence intensity of each protein on the line is set to 1.0. Source data are provided as a Source Data file.

Fig.S2

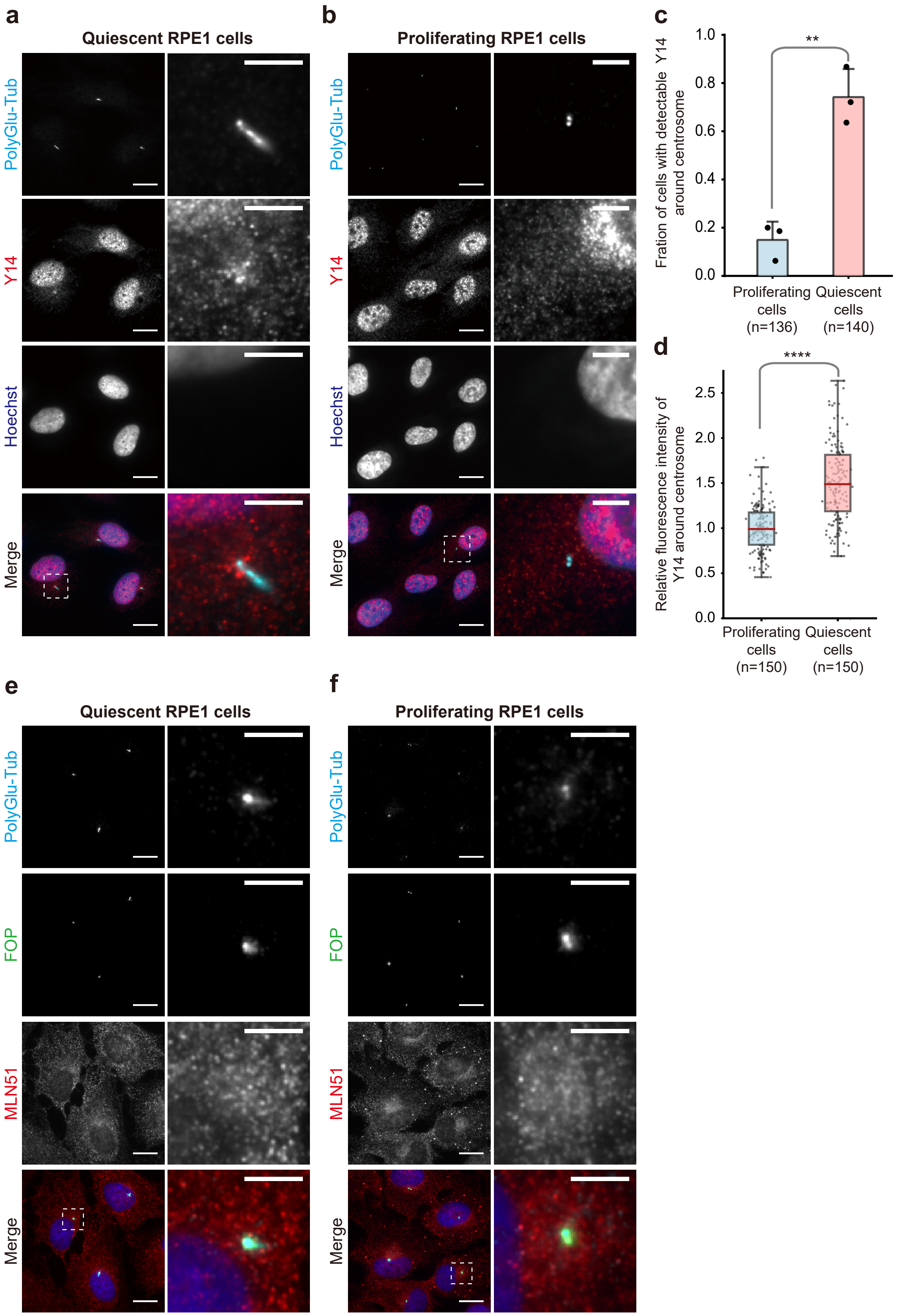
a
Quiescent RPE1 cells
b
Proliferating RPE1 cells
PolyGlu-Tub
Y14
Hoechst
Merge
c
Fration of cells with detectable Y14 around centrosome
**
1.0
0.8
0.6
0.4
0.2
0.0
Proliferating cells (n=136)
Quiescent cells (n=140)
d
Relative fluorescence intensity of Y14 around centrosome
****
2.5
2.0
1.5
1.0
0.5
0.0
Proliferating cells (n=150)
Quiescent cells (n=150)
e
Quiescent RPE1 cells
f
Proliferating RPE1 cells
PolyGlu-Tub
FOP
MLN51
Merge

Figure S2. Y14, core component of EJC accumulates around centrosome during quiescence, while MLN51 broadly exist in cytosol.

Proliferating (a, e) and quiescent (b, f) RPE1 cells were stained for Y14 (a, b) or MLN51 (e, f). Primary cilia and centrioles were stained by poly-glutamylated tubulin (PolyGlu-Tub) antibody (a, b, e, f). Centrosomes were labeled by FOP antibody (e, f). Nuclei were stained by Hoechst. Right panels show enlarged images of the white dashed square in left. Scale bars in left and right panels are 10 μm and 3 μm, respectively (a, b, e, f). The fraction of cells with detectable Y14 was determined in either proliferating or quiescent RPE1 cells. Columns and bars depict Mean + S.D of three independent experiments (c). Quantifications of Y14 fluorescence intensities were performed as described in the legend of figure 1 except that average fluorescence intensity of Y14 in proliferating cells is set to 1.0. Boxes represent values between the 25th lower and 75th higher percentile, and the red lines mark the median. Whiskers above and below correspond to 0.35th lower and 99.65th higher percentile, respectively (d). ** $P \leq 0.01$, and **** $P \leq 0.0001$, two-tailed t-test (c) and two-tiled Mann-Whitney test (d). The number of cells analyzed in three independent experiments is provided (c, d). Source data are provided as a Source Data file.

Fig.S3

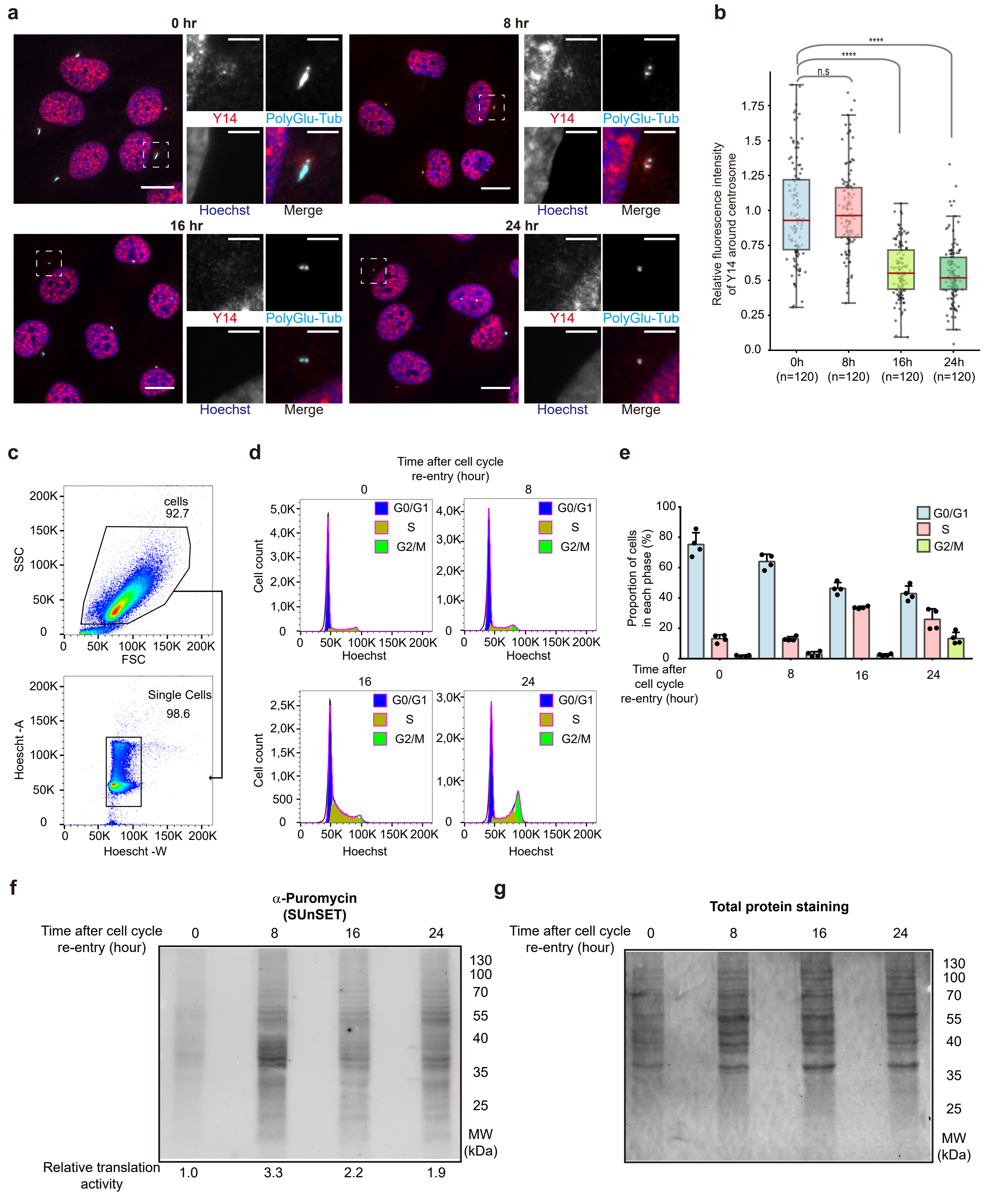
a
0 hr
8 hr
16 hr
24 hr
Y14
PolyGlu-Tub
Hoechst
Merge
b
Relative fluorescence intensity of Y14 around centrosome
n.s
****
****
0h (n=120)
8h (n=120)
16h (n=120)
24h (n=120)
c
cells 92.7
SSC
FSC
Single Cells 98.6
Hoescht -A
Hoescht -W
d
Time after cell cycle re-entry (hour)
0
8
16
24
G0/G1
S
G2/M
Cell count
Hoechst
e
Proportion of cells in each phase (%)
Time after cell cycle re-entry (hour)
f
α-Puromycin (SUnSET)
Time after cell cycle re-entry (hour)
0
8
16
24
130
100
70
55
40
35
25
MW (kDa)
Relative translation activity
1.0
3.3
2.2
1.9
g
Total protein staining
Time after cell cycle re-entry (hour)

Figure S3. Y14 around centrosome, RPE1 cell cycle and general translation activity upon cell cycle re-entry.

Quiescent RPE1 cells were incubated with 10 % serum containing media during the indicated times, and centrosomal Y14, cell cycle and translation efficiency were analyzed by immunofluorescence (a, b), flow cytometry (c, d), and SUnSET (e), respectively. Primary cilia and centriole were stained by poly-glutamylated tubulin (PolyGlu-Tub) antibody. Nuclei were stained by Hoechst. Right panels show enlarged images of the white dashed square in the left panel. Scale bars in the left panels are 10 μm, and scale bars in right panels are 3 μm (a). Quantification of Y14 fluorescence intensities was performed as described in the legend of figure 1. Average fluorescence intensity of Y14 in cells with 0 hr incubation is set to 1.0. Boxes represent values between the 25th lower and 75th higher percentile, and the red lines mark the median. Whiskers above and below correspond to 0.35th lower and 99.65th higher percentile, respectively. n.s $P > 0.05$ and **** $P \leq 0.0001$, two tailed Mann-Whitney test. The number of cells analyzed in three independent experiments is depicted (b). Gating strategy to analyze cell cycle is represented in c. Cells were selected based on SSC and FSC. Doublets of cells were excluded based on Hoechst-A and Hoechst-W (c). Representative cell cycle profiles at each incubation times are depicted (d). Cell cycle was determined by Hoechst staining. The proportion of cells in each phase was analyzed by FlowJo. Columns and bars depict Mean + S.D of four independent experiments (e). Nascent peptides were visualized by western blot with puromycin antibody (f), and average relative translation activity was normalized by total protein staining in three independent experiments (f, g). Translation efficiency at 0 hr is set to 1.0. Three independent experiments were performed (a, b, e, f). Source data are provided as a Source Data file.

Fig.S4

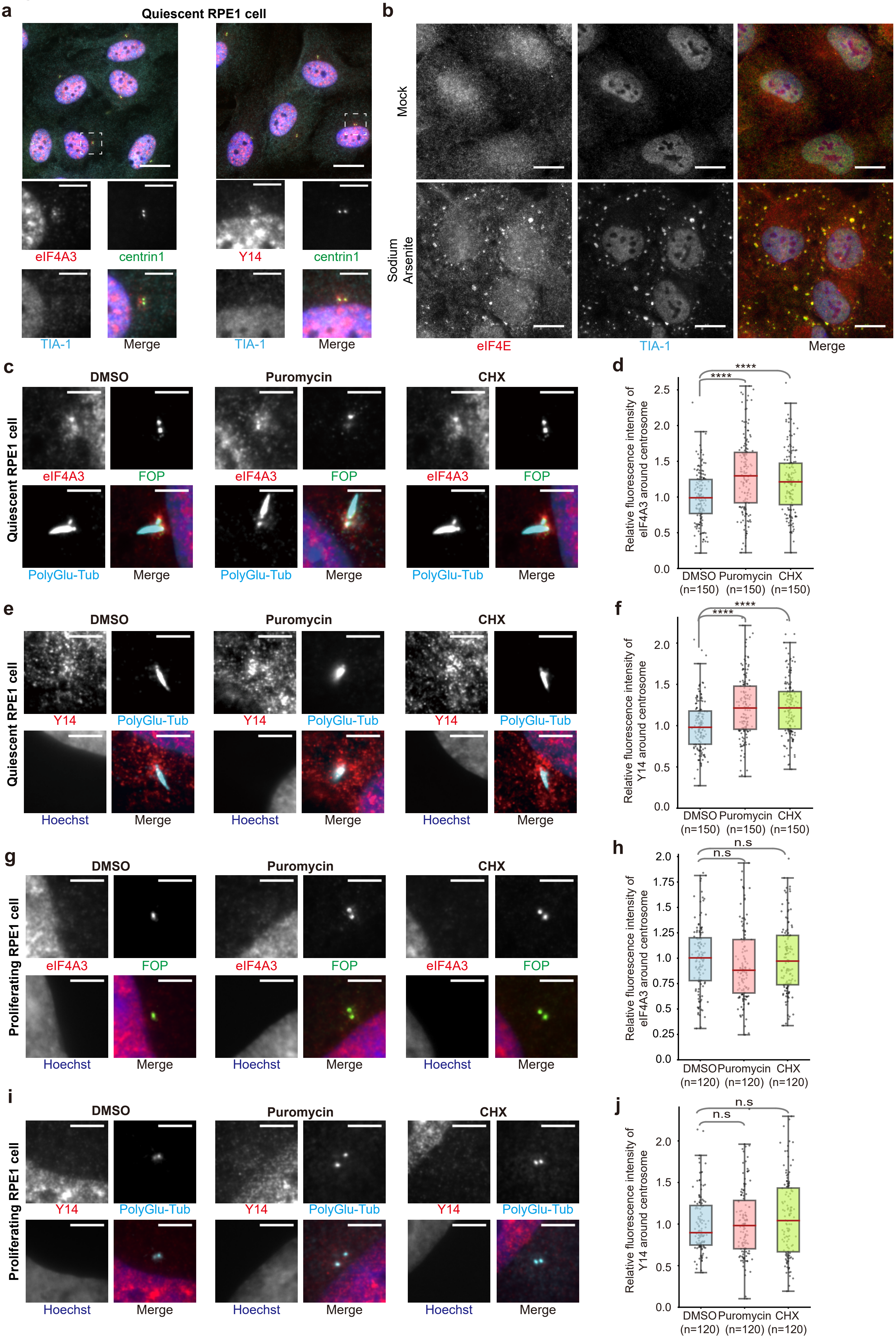
a
Quiescent RPE1 cell
eIF4A3
centrin1
Y14
centrin1
TIA-1
Merge
TIA-1
Merge
b
Mock
Sodium Arsenite
eIF4E
TIA-1
Merge
c
DMSO
Puromycin
CHX
Quiescent RPE1 cell
eIF4A3
FOP
PolyGlu-Tub
Merge
d
Relative fluorescence intensity of eIF4A3 around centrosome
****
****
DMSO (n=150)
Puromycin (n=150)
CHX (n=150)
e
DMSO
Puromycin
CHX
Quiescent RPE1 cell
Y14
PolyGlu-Tub
Hoechst
Merge
f
Relative fluorescence intensity of Y14 around centrosome
****
****
DMSO (n=150)
Puromycin (n=150)
CHX (n=150)
g
DMSO
Puromycin
CHX
Proliferating RPE1 cell
eIF4A3
FOP
Hoechst
Merge
h
Relative fluorescence intensity of eIF4A3 around centrosome
n.s
n.s
DMSO (n=120)
Puromycin (n=120)
CHX (n=120)
i
DMSO
Puromycin
CHX
Proliferating RPE1 cell
Y14
PolyGlu-Tub
Hoechst
Merge
j
Relative fluorescence intensity of Y14 around centrosome
n.s
n.s
DMSO (n=120)
Puromycin (n=120)
CHX (n=120)

Figure S4. Stress granules and general translation independent EJC accumulation around centrosome.

Quiescent RPE1 cells stably expressing centrin1-GFP were stained for stress granule marker protein TIA1 and either eIF4A3 or Y14 (a). Lower panels show enlarged images marked by white dashed square in the upper panel (a). RPE1 cells were treated with sodium arsenite or not (b) prior to fixation and stained with eIF4E and TIA-1 (b). Quiescent RPE1 cell (c, e) or proliferating RPE1 cells (g, i) were treated with DMSO, Puromycin, or Cycloheximide (CHX) prior to fixation and stained with either eIF4A3 (c, g) or Y14 (e, i) antibodies. Centrioles and cilia were stained by poly-glutamylated tubulin (PolyGlu-Tub) antibody (c, e, i) and centrosomes were labeled with FOP antibody (c, g). Nuclei were stained by Hoechst. Scale bars in the upper and lower panels are 10 μm and 3 μm, respectively (a). Scale bars in b and scale bars in c, e, g, i are 10 μm and 3 μm, respectively. Quantifications of fluorescence intensities of eIF4A3 (d, h) and Y14 (f, j) were performed as described in the legend of figure 1. The average fluorescence intensities of eIF4A3 or Y14 in DMSO treated cells are set to 1.0. Boxes represent values between the 25th lower and 75th higher percentile, and the red lines mark the median. Whiskers above and below correspond to 0.35th lower and 99.65th higher percentile, respectively. The numbers of cells analyzed from three independent experiments are depicted. n.s $P > 0.05$ and **** $P \leq 0.0001$, two-tailed Mann-Whitney test (d, f, h, j). Source data are provided as a Source Data file.

Fig.S5

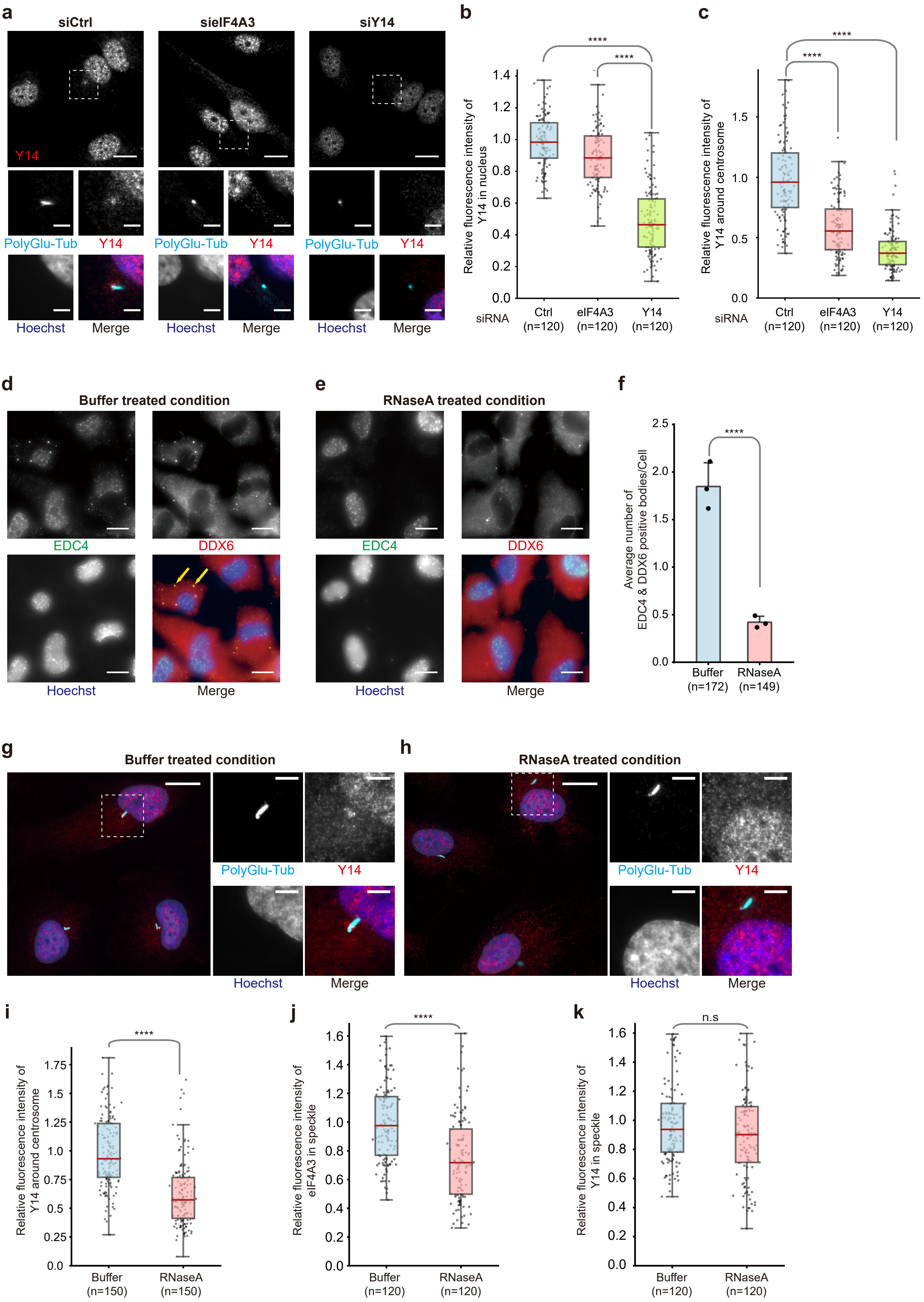
a
siCtrl
sieIF4A3
siY14
Y14
PolyGlu-Tub
Y14
Hoechst
Merge
b
Relative fluorescence intensity of Y14 in nucleus
siRNA
Ctrl (n=120)
eIF4A3 (n=120)
Y14 (n=120)
****
c
Relative fluorescence intensity of Y14 around centrosome
d
Buffer treated condition
EDC4
DDX6
Hoechst
Merge
e
RNaseA treated condition
f
Average number of EDC4 & DDX6 positive bodies/Cell
Buffer (n=172)
RNaseA (n=149)
g
Buffer treated condition
h
RNaseA treated condition
i
Relative fluorescence intensity of Y14 around centrosome
Buffer (n=150)
RNaseA (n=150)
j
Relative fluorescence intensity of eIF4A3 in speckle
Buffer (n=120)
RNaseA (n=120)
k
Relative fluorescence intensity of Y14 in speckle
n.s

Figure S5. RNA-dependent localization of assembled EJC around centrosome.

Y14 antibody stained quiescent RPE1 cells treated with indicated siRNAs (a). Permeabilized proliferating RPE1 cells (d, e) and quiescent (g, h) were incubated with RNAse A or not prior to fixation and stained for EDC4 and DDX6 (d, e) or Y14 (g, h). Primary cilia and centriole were stained by poly-glutamylated tubulin (PolyGlu-Tub) antibody (a, g, h). Nuclei were stained by Hoechst. Upper (a) or right (g, h) panels show enlarged images marked by white dashed square in upper (a) or left panels (g, h). Scale bars in upper (a) or left (g, h) and lower (a) or right panels (g, h) are 10 μm and 3 μm, respectively. Scale bars in d and e are 10 μm. Yellow arrows represent EDC4 and DDX6 double positive bodies (d). Quantifications of fluorescence intensities for Y14 in the nucleus (b) were determined in Hoechst stained area. Relative fluorescence intensity of Y14 around centrosomes (c, i) or in speckles (j, k) were determined in 2 μm circle around centrosome (c, i) or 1 μm on speckles (j, k), respectively. One 1 μm circle per cell were manually selected in nuclei. The average fluorescence intensity of Y14 in Ctrl siRNA treated cells (b, c) or Buffer treated condition (i-k) is set to 1.0. Boxes represent values between the 25th lower and 75th higher percentile, and the red lines mark the median. Whiskers above and below correspond to 0.35th lower and 99.65th higher percentile, respectively (b, c, i-k). Number of EDC4 and DDX6 double positive bodies/cell was determined in either buffer or RNaseA treated cells. Columns and bars depict Mean + S.D of three independent experiments (f). n.s $P > 0.05$ and **** $P \leq 0.0001$, two-tailed Mann-Whitney test (b, c, i, j, k) and two-tailed t-test (f). The numbers of cells analyzed from three independent experiments are depicted (b, c, f, i, j, k). Source data are provided as a Source Data file.

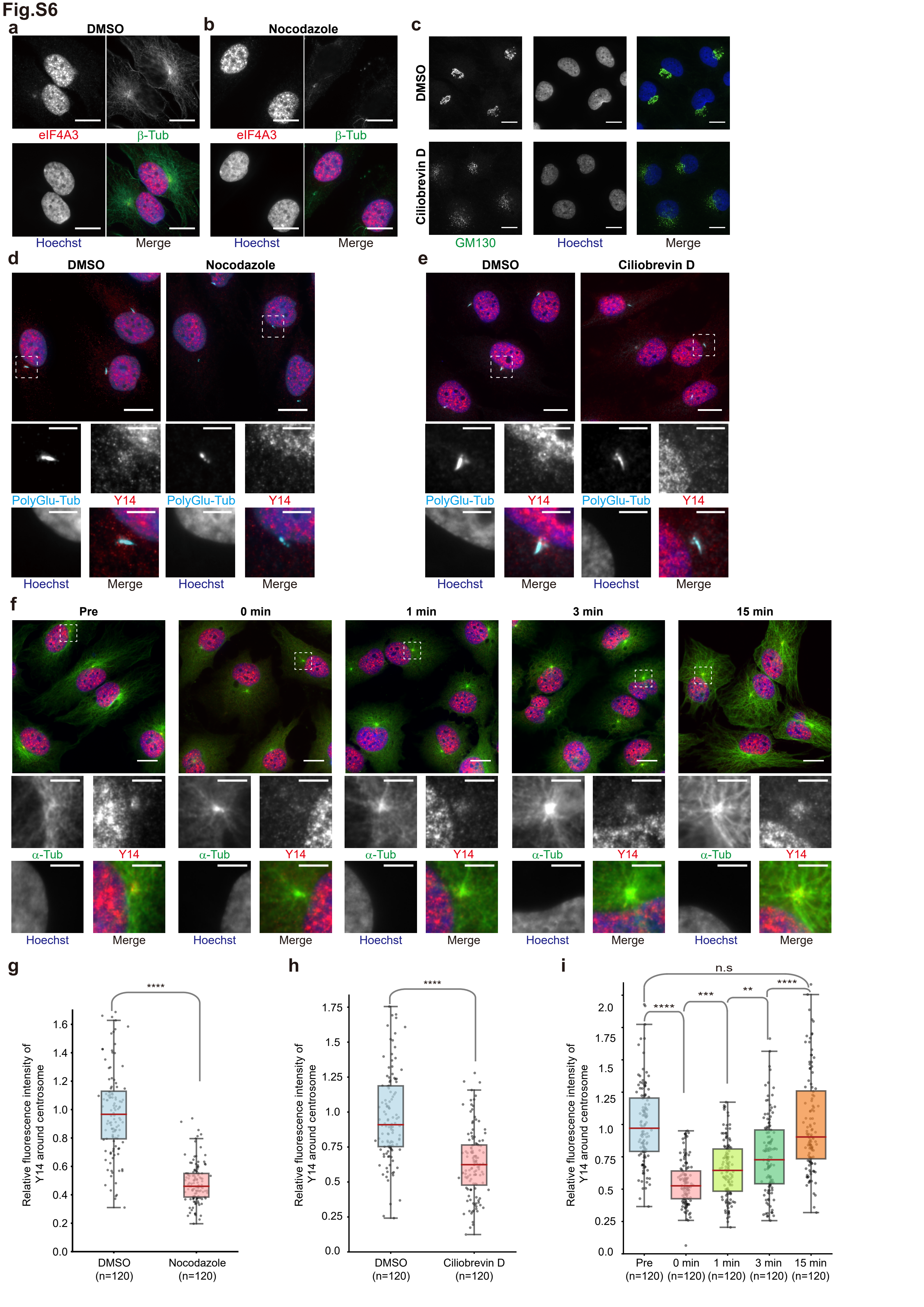
Fig.S6
a
DMSO
eIF4A3
β-Tub
Hoechst
Merge
b
Nocodazole
eIF4A3
β-Tub
Hoechst
Merge
c
DMSO
Ciliobrevin D
GM130
Hoechst
Merge
d
DMSO
Nocodazole
PolyGlu-Tub
Y14
Hoechst
Merge
e
DMSO
Ciliobrevin D
PolyGlu-Tub
Y14
Hoechst
Merge
f
Pre
0 min
1 min
3 min
15 min
α-Tub
Y14
Hoechst
Merge
g
Relative fluorescence intensity of Y14 around centrosome
****
DMSO (n=120)
Nocodazole (n=120)
h
Relative fluorescence intensity of Y14 around centrosome
****
DMSO (n=120)
Ciliobrevin D (n=120)
i
Relative fluorescence intensity of Y14 around centrosome
n.s
****
***
**
****
Pre (n=120)
0 min (n=120)
1 min (n=120)
3 min (n=120)
15 min (n=120)

Figure S6. Dynein dependent transport along microtubule is required for Y14 accumulation around centrosomes.

Proliferating RPE1 cells were treated with either DMSO or Nocodaole (a, b) or either DMSO or ciliobrevinD (c) and stained with eIF4A3 and β-tubulin (a, b) or GM130 (c) antibodies. Scale bars are 10 μm (a-c). Y14 antibody stained quiescent cells treated with either DMSO or Nocodazole (d), either DMSO or CiliobrevinD (e), or chilled quiescent cells subjected to a microtubule regrowth assay (f). Primary cilia and centriole were stained by poly-glutamylated tubulin (PolyGlu-Tub) antibody (d, e) and microtubules were visualized by α-tubulin (α-Tub) antibody. Nuclei were stained by Hoechst. Lower panels show enlarged images marked by white dashed square in the upper panel (d-f). Scale bars in upper and lower panels represent 10 μm and 3 μm, respectively (d-f). Quantification of fluorescence intensities of Y14 (g-i) was performed as described in the legend of figure 1. The average fluorescence intensities for Y14 in DMSO treated cells (g, h) or in pre-incubated quiescent cells (i) are set to 1.0. Boxes represent values between the 25th lower and 75th higher percentile, and the red lines mark the median. Whiskers above and below correspond to 0.35th lower and 99.65th higher percentile, respectively. n.s $P > 0.05$, ** $P \leq 0.01$, *** $P \leq 0.001$, and **** $P \leq 0.0001$, two tailed Mann-Whitney test. The numbers of cells analyzed from three independent experiments are depicted (g-i). Source data are provided as a Source Data file.

Fig.S7

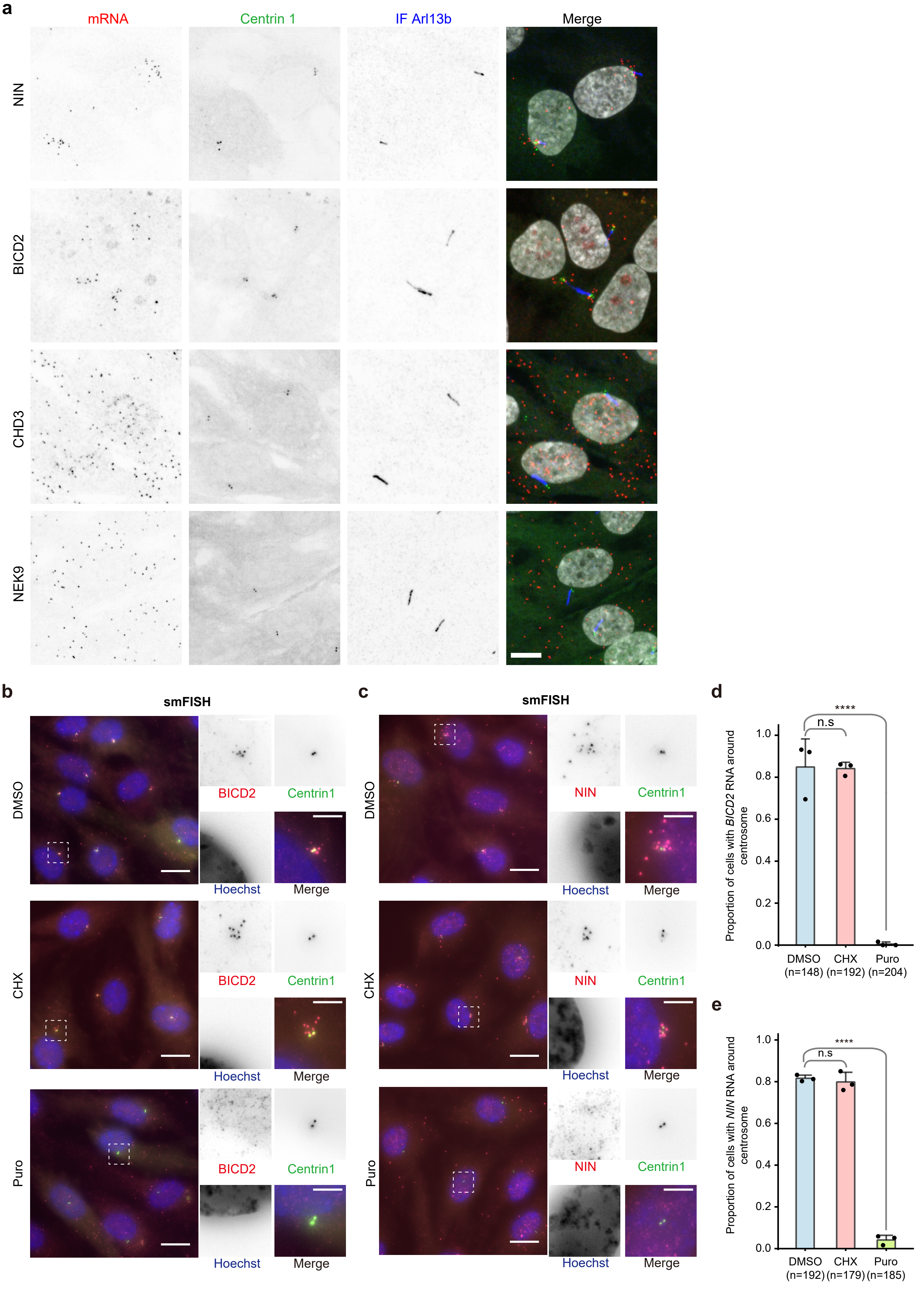
a
mRNA
Centrin 1
IF Arl13b
Merge
NIN
BICD2
CHD3
NEK9
b
smFISH
DMSO
CHX
Puro
BICD2
Centrin1
Hoechst
Merge
c
smFISH
NIN
Centrin1
Hoechst
Merge
d
Proportion of cells with BICD2 RNA around centrosome
****
n.s
1.0
0.8
0.6
0.4
0.2
0.0
DMSO (n=148)
CHX (n=192)
Puro (n=204)
e
Proportion of cells with NIN RNA around centrosome
****
n.s
DMSO (n=192)
CHX (n=179)
Puro (n=185)

Figure S7. A high-throughput smiFISH screen identifies mRNAs localizing to the base of cilia and translating ribosome is required for localization of *NIN* and *BICD2* mRNAs.

In a high-throughput smiFISH screen, quiescent RPE1 cells stably expressing Centrin1-GFP were stained for each indicated RNA and Arl13b. Nuclei were labeled with DAPI. Images were processed by maximum intensity projections of 35 z-stacks acquired at every 0.35 μm. Scale bar represents 10 μm (a). Quiescent RPE1 cells stably expressing centrin1-GFP were stained by probes against *BICD2* mRNA (b) or *NIN* mRNA (c) after 30 min incubation with either DMSO, cyloheximide (CHX), or puromycin (Puro). Right panels display enlarged images of the white dashed square in the left panel. Scale bars in the left panels are 10 μm, and scale bars in right panels are 3 μm. Images result from maximum intensity projections of 14 z-stacks acquired at every 0.5 μm (b, c). Proportion of cells displaying centrosomal *BICD2* (d) or *NIN* (e) RNA pattern is depicted. Columns and bars represent Mean + S.D of three independent experiments. Cell numbers analyzed in three independent experiments are depicted. n.s $P > 0.05$ and **** $P \leq 0.0001$, two-tailed t-test (d, e). Source data are provided as a Source Data file.

Fig.S8

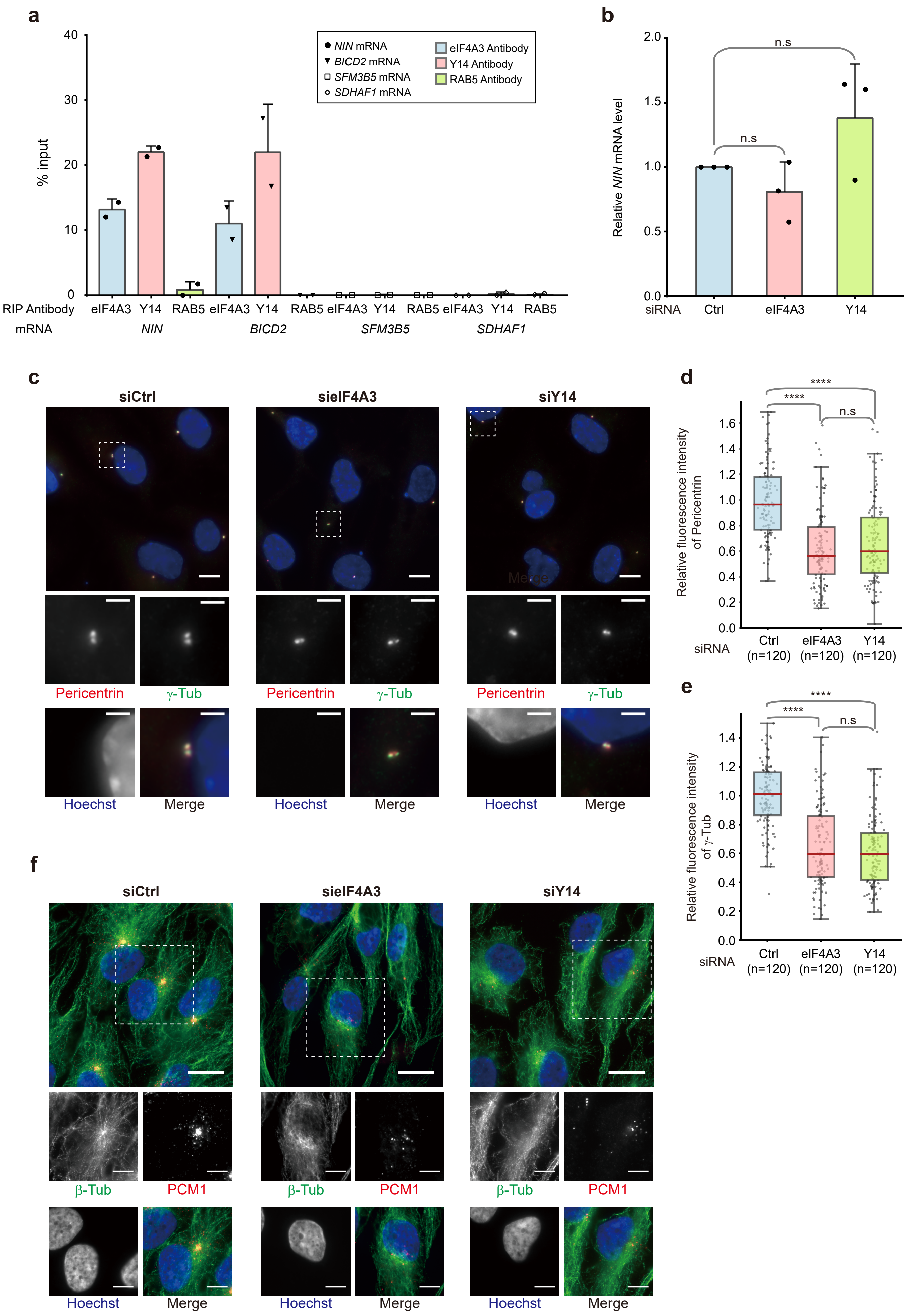
a
% input
40
30
20
10
0
NIN mRNA
BICD2 mRNA
SFM3B5 mRNA
SDHAF1 mRNA
eIF4A3 Antibody
Y14 Antibody
RAB5 Antibody
RIP Antibody
eIF4A3 Y14 RAB5 eIF4A3 Y14 RAB5 eIF4A3 Y14 RAB5 eIF4A3 Y14 RAB5
mRNA
NIN
BICD2
SFM3B5
SDHAF1
b
Relative NIN mRNA level
2.0
1.5
1.0
0.5
0.0
n.s
n.s
siRNA
Ctrl
eIF4A3
Y14
c
siCtrl
sieIF4A3
siY14
Pericentrin
γ-Tub
Hoechst
Merge
d
Relative fluorescence intensity of Pericentrin
****
****
n.s
1.6
1.4
1.2
1.0
0.8
0.6
0.4
0.2
0.0
siRNA
Ctrl (n=120)
eIF4A3 (n=120)
Y14 (n=120)
e
Relative fluorescence intensity of γ-Tub
****
****
n.s
1.4
1.2
1.0
0.8
0.6
0.4
0.2
0.0
siRNA
Ctrl (n=120)
eIF4A3 (n=120)
Y14 (n=120)
f
siCtrl
sieIF4A3
siY14
β-Tub
PCM1
Hoechst
Merge

Figure S8. Both *NIN* and *BICD2* mRNAs are associated with EJC during quiescence and knock-downs of eIF4A3 and Y14 alter pericentriolar material organization. The association of EJC with centrosomal (*NIN* and *BICD2*) and intronless (*SFM3B5* and *SDHAF1*) mRNAs in quiescent RPE1 cells was determined by RNA immunoprecipitation (RIP)-qPCR analysis of eIF4A3, Y14, and Rab5. Enriched mRNA levels in pull down fraction compare to input from are depicted as % input (a). Quiescent RPE1 cells treated with indicated siRNAs were analyzed for *NIN* mRNA levels by RT-qPCR. Average *NIN* mRNA levels normalized by *GAPDH* mRNA levels are depicted. Ctrl siRNA treated condition is set to 1.0 (b). Columns and bars represent Mean + S.D of two (a) or three (b) independent experiments. Quiescent RPE1 cells transfected with Ctrl, eIF4A3 or Y14 siRNA (c, f) were stained for pericentrin and γ-tubulin (c) or for β-tubulin and PCM1 (f). Images were resulted from maximum intensity projections of 6 z-stacks acquired at every 0.5 μm (c). Lower panels are enlarged images marked by white dashed square in the upper panels (c, f). Scale bars in upper panels of c and f are 10 μm and scale bars in the lower panels of c and f are 3 μm and 5 μm respectively. Quantifications of fluorescence intensities for pericentrin (d) and γ-tubulin (e) were performed as described in the legend of figure 1 except that the average fluorescence intensities of pericentrin and γ-tubulin in Ctrl siRNA treated cells are set to 1.0. Boxes represent values between the 25th lower and 75th higher percentile, and the red lines mark the median. Whiskers above and below correspond to 0.35th lower and 99.65th higher percentile, respectively. Cell numbers analyzed in three independent experiments are depicted (d, e). n.s $P > 0.05$, **** $P \leq 0.0001$. two-tailed t-test (a) and two-tailed Mann-Whitney test (d, e). Source data are provided as a Source Data file.

Fig.S9

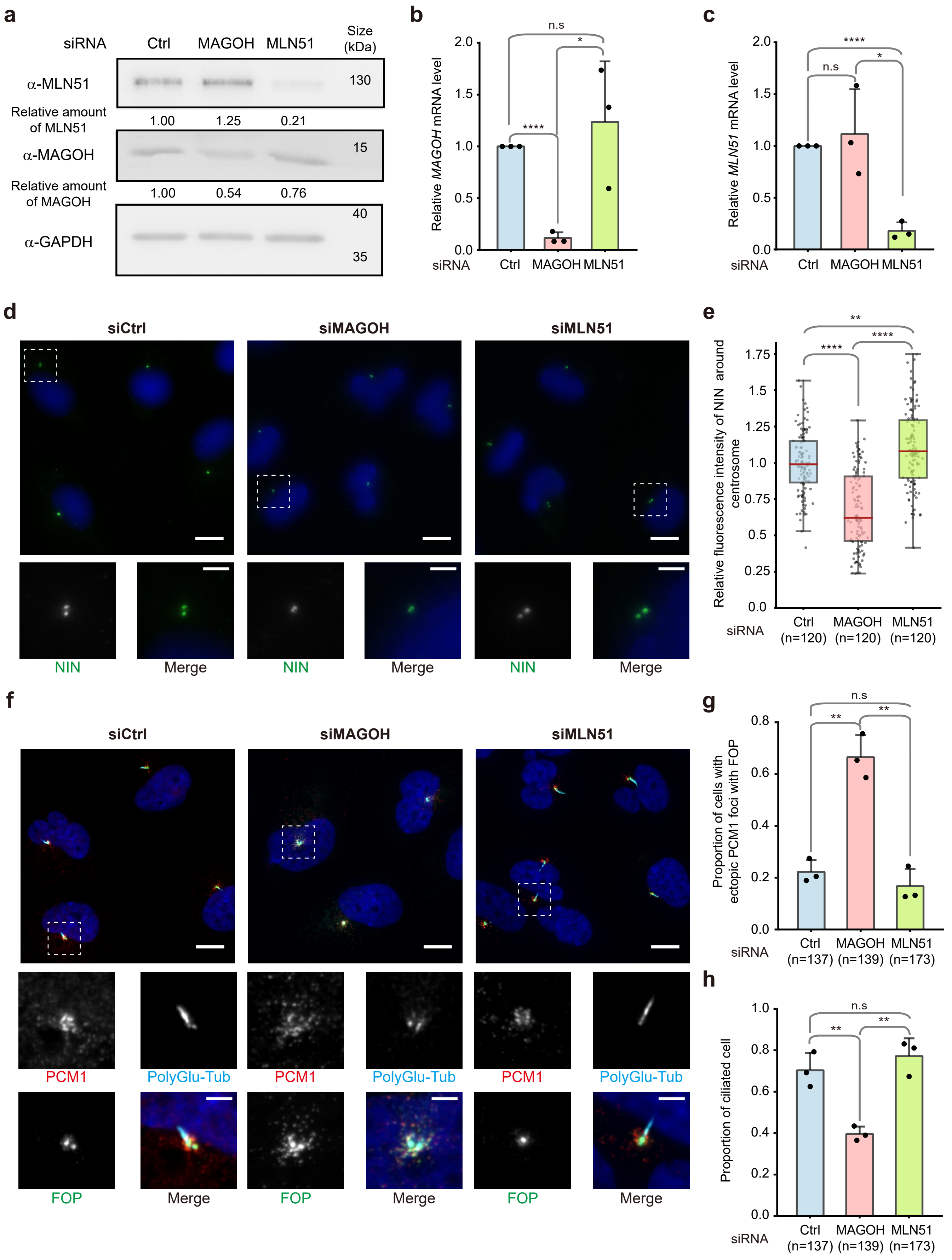

a
siRNA
Ctrl
MAGOH
MLN51
Size (kDa)
α-MLN51
130
Relative amount of MLN51
1.00
1.25
0.21
α-MAGOH
15
Relative amount of MAGOH
1.00
0.54
0.76
α-GAPDH
40
35
b
Relative MAGOH mRNA level
n.s
*
****
2.0
1.5
1.0
0.5
0.0
siRNA
Ctrl
MAGOH
MLN51
c
Relative MLN51 mRNA level
****
n.s
*
siRNA
Ctrl
MAGOH
MLN51
d
siCtrl
siMAGOH
siMLN51
NIN
Merge
e
Relative fluorescence intensity of NIN around centrosome
**
****
****
1.75
1.5
1.25
1.0
0.75
0.5
0.25
0.0
siRNA
Ctrl (n=120)
MAGOH (n=120)
MLN51 (n=120)
f
siCtrl
siMAGOH
siMLN51
PCM1
PolyGlu-Tub
FOP
Merge
g
Proportion of cells with ectopic PCM1 foci with FOP
n.s
**
**
0.8
0.6
0.4
0.2
0.0
siRNA
Ctrl (n=137)
MAGOH (n=139)
MLN51 (n=173)
h
Proportion of ciliated cell
n.s
**
**
1.0
0.8
0.6
0.4
0.2
0.0
siRNA
Ctrl (n=137)
MAGOH (n=139)
MLN51 (n=173)

Figure S9. Depletion of MAGOH but not of MLN51 affects NIN protein level at centrosomes and centrosome organization.

Knock-down efficiency of siRNAs was determined by either Western blotting (a) or RT-qPCR (b, c). Relative protein (a) or RNA level of MAGOH (b) and MLN51 (c) normalized by GAPDH is depicted. Ctrl siRNA treated condition is set to 1.0 (a-c). Quiescent RPE1 cells transfected with indicated siRNAs were stained for NIN (d) or PCM1, FOP, and poly-glutamylated tubulin (f). Nuclei were stained by Hoechst. Lower panels are enlarged images marked by white dashed square in the upper panels. Scale bars in the upper and lower panels are 10 μm and 3 μm, respectively (d, f). Images were processed by maximum intensity projections of 15 z-stacks acquired at every 0.5 μm (d). Quantification of fluorescence intensities of NIN were performed as described in the legend of figure 1. The average fluorescence intensity of NIN in Ctrl siRNA treated cells is set to 1.0. Boxes represent values between the 25th lower and 75th higher percentile, and the red lines mark the median. Whiskers above and below correspond to 0.35th lower and 99.65th higher percentile, respectively (e). Proportion of cells with ectopic centriolar satellite with FOP upon the siRNA treatments is represented (g). Proportion of ciliated cells upon the indicated siRNA treatments (h). Columns and bars represent Mean + S.D of three independent experiments (b, c, g, h). n.s $P > 0.05$, * $P \leq 0.05$, ** $P \leq 0.01$, and **** $P \leq 0.0001$, two-tailed Mann-Whitney test (e) and two-tailed t-test (b, c, g, h). Cell numbers analyzed in three independent experiments are depicted (e, g, h). Source data are provided as a Source Data file.

Supplementary Table. S1

Primer information		
Gene	Primer	Sequence (5' to 3')
NIN	RT	CAGTTACCAGTTGTTCCTGG
	qPCR Forward	GCCAGGAAACAGTGCATTGG
	qPCR Reverse	CTTCAGAGAGCTCCGCCTCC
BICD2	RT	CCAGCTGTGTAATGTACTCG
	qPCR Forward	AGTTGCGCAATGTCCTCACC
	qPCR Reverse	GAGTAGTCCTGCAGCAGACG
eIF4A3	qPCR Forward	ACGAGCAATCAAGCAGATCA
	qPCR Reverse	AGGTGGCTGTTTTTCCTGTG
Y14	qPCR Forward	TGCGTGAGGATTATGACAGC
	qPCR Reverse	CATATTCTGCGAATTTGTCG
MAGOH	qPCR Forward	GAGTTTCGACCGGACGGGAA
	qPCR Reverse	GATTTCAAGCTCCTGCCGGC
MLN51	qPCR Forward	AGCCTG ACACCAAAAGCACT
	qPCR Reverse	TAGGGCCCTTTTTACCCACT
SF3B5	qPCR Forward	ACCGCTACACCATCCATAGC
	qPCR Reverse	AGGCTGAAGCATCTTTTCCA
SDHAF1	qPCR Forward	ACGAATTGGGGATGTCAGAG
	qPCR Reverse	GTCTCCAAGCATCGGAAA AG
GAPDH	qPCR Forward	TTAAAAGCAGCCCTGGTGAC
	qPCR Reverse	CTCTGCTCCTCCTGTTCGAC

Table S1. The information of primers.

List of primer sequences used for RT-qPCR.
