# Supplementary material for: Exon junction complex dependent mRNA localization is linked to centrosome organization during ciliogenesis
Source: Nat Commun. 2021 Mar 1;12:1351. doi: 10.1038/s41467-021-21590-w (PMC7921557; doi:10.1038/s41467-021-21590-w)
Supplement: Supplementary file 2 — Supplementary Data 1 [file 41467_2021_21590_MOESM2_ESM.pdf]

Supplementary DATA 1

ENSG	ENST	GeneName	Cell line	**Localization**
ENSG00000185963	ENST00000375512	BICD2	Diff. RPE1 GFP-Centrin	Cilium base
ENSG00000100503	ENST00000389868	NIN	Diff. RPE1 GFP-Centrin	Cilium base
ENSG00000122545	ENST00000399035	SEPT7	Diff. RPE1 GFP-Centrin	Clusters
ENSG00000138160	ENST00000260731	KIF11	Diff. RPE1 GFP-Centrin	Clusters
ENSG00000197102	ENST00000555062	DYNC1H1	Diff. RPE1 GFP-Centrin	Foci
ENSG00000107581	ENST00000369144	EIF3A	Diff. RPE1 GFP-Centrin	Foci
ENSG00000163346	ENST00000368463	PBXIP1	Diff. RPE1 GFP-Centrin	Foci
ENSG00000273217	ENST00000514667	AC008695.1	Diff. RPE1 GFP-Centrin	no signal
ENSG00000115073	ENST00000289228	ACTR1B	Diff. RPE1 GFP-Centrin	no signal
ENSG00000165923	ENST00000525123	AGBL2	Diff. RPE1 GFP-Centrin	no signal
ENSG00000186094	ENST00000371839	AGBL4	Diff. RPE1 GFP-Centrin	no signal
ENSG00000169126	ENST00000305242	ARMC4	Diff. RPE1 GFP-Centrin	no signal
ENSG00000039987	ENST00000042931	BEST2	Diff. RPE1 GFP-Centrin	no signal
ENSG00000135127	ENST00000397558	BICDL1	Diff. RPE1 GFP-Centrin	no signal
ENSG00000160469	ENST00000309383	BRSK1	Diff. RPE1 GFP-Centrin	no signal
ENSG00000154493	ENST00000284694	C10ORF90	Diff. RPE1 GFP-Centrin	no signal
ENSG00000205129	ENST00000378850	C4ORF47	Diff. RPE1 GFP-Centrin	no signal
ENSG00000181751	ENST00000510890	C5ORF30	Diff. RPE1 GFP-Centrin	no signal
ENSG00000004948	ENST00000360249	CALCR	Diff. RPE1 GFP-Centrin	no signal
ENSG00000076826	ENST00000446248	CAMSAP3	Diff. RPE1 GFP-Centrin	no signal
ENSG00000133962	ENST00000256343	CATSPERB	Diff. RPE1 GFP-Centrin	no signal
ENSG00000174898	ENST00000381624	CATSPERD	Diff. RPE1 GFP-Centrin	no signal
ENSG00000161180	ENST00000292779	CCDC116	Diff. RPE1 GFP-Centrin	no signal
ENSG00000244607	ENST00000310232	CCDC13	Diff. RPE1 GFP-Centrin	no signal
ENSG00000135205	ENST00000285871	CCDC146	Diff. RPE1 GFP-Centrin	no signal
ENSG00000182645	ENST00000333254	CCDC172	Diff. RPE1 GFP-Centrin	no signal
ENSG00000160050	ENST00000373602	CCDC28B	Diff. RPE1 GFP-Centrin	no signal
ENSG00000145075	ENST00000273654	CCDC39	Diff. RPE1 GFP-Centrin	no signal
ENSG00000166510	ENST00000591504	CCDC68	Diff. RPE1 GFP-Centrin	no signal
ENSG00000015133	ENST00000389857	CCDC88C	Diff. RPE1 GFP-Centrin	no signal
ENSG00000079335	ENST00000361544	CDC14A	Diff. RPE1 GFP-Centrin	no signal
ENSG00000170312	ENST00000395284	CDK1	Diff. RPE1 GFP-Centrin	no signal
ENSG00000100629	ENST00000281129	CEP128	Diff. RPE1 GFP-Centrin	no signal
ENSG00000154608	ENST00000502249	CEP170P1	Diff. RPE1 GFP-Centrin	no signal
ENSG00000112877	ENST00000264935	CEP72	Diff. RPE1 GFP-Centrin	no signal
ENSG00000101624	ENST00000262127	CEP76	Diff. RPE1 GFP-Centrin	no signal
ENSG00000111860	ENST00000368488	CEP85L	Diff. RPE1 GFP-Centrin	no signal
ENSG00000121289	ENST00000305768	CEP89	Diff. RPE1 GFP-Centrin	no signal
ENSG00000163885	ENST00000352312	CFAP100	Diff. RPE1 GFP-Centrin	no signal
ENSG00000188931	ENST00000367974	CFAP126	Diff. RPE1 GFP-Centrin	no signal
ENSG00000160401	ENST00000373295	CFAP157	Diff. RPE1 GFP-Centrin	no signal
ENSG00000070761	ENST00000262498	CFAP20	Diff. RPE1 GFP-Centrin	no signal
ENSG00000272514	ENST00000369562	CFAP206	Diff. RPE1 GFP-Centrin	no signal
ENSG00000163075	ENST00000413057	CFAP221	Diff. RPE1 GFP-Centrin	no signal
ENSG00000213085	ENST00000368099	CFAP45	Diff. RPE1 GFP-Centrin	no signal
ENSG00000171811	ENST00000368586	CFAP46	Diff. RPE1 GFP-Centrin	no signal
ENSG00000172361	ENST00000398545	CFAP53	Diff. RPE1 GFP-Centrin	no signal
ENSG00000156042	ENST00000310715	CFAP70	Diff. RPE1 GFP-Centrin	no signal
ENSG00000186710	ENST00000335621	CFAP73	Diff. RPE1 GFP-Centrin	no signal
ENSG00000169607	ENST00000541405	CKAP2L	Diff. RPE1 GFP-Centrin	no signal

ENSG00000126890	ENST00000247306	CTAG2	Diff. RPE1 GFP-Centrin	no signal
ENSG00000165325	ENST00000298050	DEUP1	Diff. RPE1 GFP-Centrin	no signal
ENSG00000166938	ENST00000319194	DIS3L	Diff. RPE1 GFP-Centrin	no signal
ENSG00000166938	ENST00000319194	DIS3L	Diff. RPE1 GFP-Centrin	no signal
ENSG00000105877	ENST00000409508	DNAH11	Diff. RPE1 GFP-Centrin	no signal
ENSG00000174844	ENST00000311202	DNAH12	Diff. RPE1 GFP-Centrin	no signal
ENSG00000158486	ENST00000261383	DNAH3	Diff. RPE1 GFP-Centrin	no signal
ENSG00000039139	ENST00000265104	DNAH5	Diff. RPE1 GFP-Centrin	no signal
ENSG00000115423	ENST00000389394	DNAH6	Diff. RPE1 GFP-Centrin	no signal
ENSG00000118997	ENST00000312428	DNAH7	Diff. RPE1 GFP-Centrin	no signal
ENSG00000124721	ENST00000327475	DNAH8	Diff. RPE1 GFP-Centrin	no signal
ENSG00000197959	ENST00000367731	DNM3	Diff. RPE1 GFP-Centrin	no signal
ENSG00000144635	ENST00000273130	DYNC1LI1	Diff. RPE1 GFP-Centrin	no signal
ENSG00000096093	ENST00000371068	EFHC1	Diff. RPE1 GFP-Centrin	no signal
ENSG00000013016	ENST00000322054	EHD3	Diff. RPE1 GFP-Centrin	no signal
ENSG00000151023	ENST00000376363	ENKUR	Diff. RPE1 GFP-Centrin	no signal
ENSG00000165689	ENST00000371725	ENTR1	Diff. RPE1 GFP-Centrin	no signal
ENSG00000173040	ENST00000344408	EVC2	Diff. RPE1 GFP-Centrin	no signal
ENSG00000182263	ENST00000333129	FIGN	Diff. RPE1 GFP-Centrin	no signal
ENSG00000189139	ENST00000340446	FSCB	Diff. RPE1 GFP-Centrin	no signal
ENSG00000065135	ENST00000369851	GNAI3	Diff. RPE1 GFP-Centrin	no signal
ENSG00000215203	ENST00000399770	GRXCR1	Diff. RPE1 GFP-Centrin	no signal
ENSG00000177602	ENST00000325418	GSG2	Diff. RPE1 GFP-Centrin	no signal
ENSG00000092036	ENST00000342454	HAUS4	Diff. RPE1 GFP-Centrin	no signal
ENSG00000131351	ENST00000253669	HAUS8	Diff. RPE1 GFP-Centrin	no signal
ENSG00000131351	ENST00000253669	HAUS8	Diff. RPE1 GFP-Centrin	no signal
ENSG00000188175	ENST00000394468	HEPACAM2	Diff. RPE1 GFP-Centrin	no signal
ENSG00000188175	ENST00000341723	HEPACAM2	Diff. RPE1 GFP-Centrin	no signal
ENSG00000134709	ENST00000371208	HOOK1	Diff. RPE1 GFP-Centrin	no signal
ENSG00000158748	ENST00000289753	HTR6	Diff. RPE1 GFP-Centrin	no signal
ENSG00000157423	ENST00000393567	HYDIN	Diff. RPE1 GFP-Centrin	no signal
ENSG00000130294	ENST00000648680	KIF1A	Diff. RPE1 GFP-Centrin	no signal
ENSG00000125337	ENST00000351261	KIF25	Diff. RPE1 GFP-Centrin	no signal
ENSG00000182866	ENST00000336890	LCK	Diff. RPE1 GFP-Centrin	no signal
ENSG00000172264	ENST00000402914	MACROD2	Diff. RPE1 GFP-Centrin	no signal
ENSG00000111837	ENST00000313243	MAK	Diff. RPE1 GFP-Centrin	no signal
ENSG00000111837	ENST00000313243	MAK	Diff. RPE1 GFP-Centrin	no signal
ENSG00000212916	ENST00000418460	MAP10	Diff. RPE1 GFP-Centrin	no signal
ENSG00000212916	ENST00000418460	MAP10	Diff. RPE1 GFP-Centrin	no signal
ENSG00000173327	ENST00000309100	MAP3K11	Diff. RPE1 GFP-Centrin	no signal
ENSG00000180834	ENST00000318631	MAP6D1	Diff. RPE1 GFP-Centrin	no signal
ENSG00000163875	ENST00000373075	MEAF6	Diff. RPE1 GFP-Centrin	no signal
ENSG00000163875	ENST00000373075	MEAF6	Diff. RPE1 GFP-Centrin	no signal
ENSG00000243156	ENST00000441493	MICAL3	Diff. RPE1 GFP-Centrin	no signal
ENSG00000158411	ENST00000289359	MITD1	Diff. RPE1 GFP-Centrin	no signal
ENSG00000034971	ENST00000037502	MYOC	Diff. RPE1 GFP-Centrin	no signal
ENSG00000166579	ENST00000402554	NDEL1	Diff. RPE1 GFP-Centrin	no signal
ENSG00000136098	ENST00000610828	NEK3	Diff. RPE1 GFP-Centrin	no signal
ENSG00000101004	ENST00000278886	NINL	Diff. RPE1 GFP-Centrin	no signal
ENSG00000144061	ENST00000445609	NPHP1	Diff. RPE1 GFP-Centrin	no signal
ENSG00000112530	ENST00000366888	PACRG	Diff. RPE1 GFP-Centrin	no signal
ENSG00000075891	ENST00000355243	PAX2	Diff. RPE1 GFP-Centrin	no signal
ENSG00000075891	ENST00000355243	PAX2	Diff. RPE1 GFP-Centrin	no signal

ENSG00000116703	ENST00000340129	PDC	Diff. RPE1 GFP-Centrin	no signal
ENSG00000184588	ENST00000371045	PDE4B	Diff. RPE1 GFP-Centrin	no signal
ENSG00000158683	ENST00000289672	PKD1L1	Diff. RPE1 GFP-Centrin	no signal
ENSG00000170927	ENST00000340994	PKHD1	Diff. RPE1 GFP-Centrin	no signal
ENSG00000170927	ENST00000371117	PKHD1	Diff. RPE1 GFP-Centrin	no signal
ENSG00000170927	ENST00000371117	PKHD1	Diff. RPE1 GFP-Centrin	no signal
ENSG00000205038	ENST00000378402	PKHD1L1	Diff. RPE1 GFP-Centrin	no signal
ENSG00000100078	ENST00000215885	PLA2G3	Diff. RPE1 GFP-Centrin	no signal
ENSG00000178125	ENST00000324682	PPP1R42	Diff. RPE1 GFP-Centrin	no signal
ENSG00000070950	ENST00000264926	RAD18	Diff. RPE1 GFP-Centrin	no signal
ENSG00000164188	ENST00000296604	RANBP3L	Diff. RPE1 GFP-Centrin	no signal
ENSG00000165917	ENST00000298854	RAPSN	Diff. RPE1 GFP-Centrin	no signal
ENSG00000102760	ENST00000379359	RGCC	Diff. RPE1 GFP-Centrin	no signal
ENSG00000169220	ENST00000408923	RGS14	Diff. RPE1 GFP-Centrin	no signal
ENSG00000104237	ENST00000220676	RP1	Diff. RPE1 GFP-Centrin	no signal
ENSG00000102218	ENST00000218340	RP2	Diff. RPE1 GFP-Centrin	no signal
ENSG00000092200	ENST00000400017	RPGRIP1	Diff. RPE1 GFP-Centrin	no signal
ENSG00000025039	ENST00000369415	RRAGD	Diff. RPE1 GFP-Centrin	no signal
ENSG00000160188	ENST00000291536	RSPH1	Diff. RPE1 GFP-Centrin	no signal
ENSG00000111834	ENST00000229554	RSPH4A	Diff. RPE1 GFP-Centrin	no signal
ENSG00000165480	ENST00000462482	SKA3	Diff. RPE1 GFP-Centrin	no signal
ENSG00000188817	ENST00000343837	SNTN	Diff. RPE1 GFP-Centrin	no signal
ENSG00000086300	ENST00000338523	SNX10	Diff. RPE1 GFP-Centrin	no signal
ENSG00000095637	ENST00000306402	SORBS1	Diff. RPE1 GFP-Centrin	no signal
ENSG00000155761	ENST00000336338	SPAG17	Diff. RPE1 GFP-Centrin	no signal
ENSG00000123473	ENST00000371877	STIL	Diff. RPE1 GFP-Centrin	no signal
ENSG00000165730	ENST00000421961	STOX1	Diff. RPE1 GFP-Centrin	no signal
ENSG00000147642	ENST00000276646	SYBU	Diff. RPE1 GFP-Centrin	no signal
ENSG00000111490	ENST00000229088	TBC1D30	Diff. RPE1 GFP-Centrin	no signal
ENSG00000184786	ENST00000366774	TCTE3	Diff. RPE1 GFP-Centrin	no signal
ENSG00000167858	ENST00000338694	TEKT1	Diff. RPE1 GFP-Centrin	no signal
ENSG00000092850	ENST00000207457	TEKT2	Diff. RPE1 GFP-Centrin	no signal
ENSG00000125409	ENST00000395930	TEKT3	Diff. RPE1 GFP-Centrin	no signal
ENSG00000153060	ENST00000283025	TEKT5	Diff. RPE1 GFP-Centrin	no signal
ENSG00000187049	ENST00000334888	TMEM216	Diff. RPE1 GFP-Centrin	no signal
ENSG00000138100	ENST00000380075	TRIM54	Diff. RPE1 GFP-Centrin	no signal
ENSG00000111199	ENST00000261740	TRPV4	Diff. RPE1 GFP-Centrin	no signal
ENSG00000126467	ENST00000246801	TSKS	Diff. RPE1 GFP-Centrin	no signal
ENSG00000131044	ENST00000375921	TTLL9	Diff. RPE1 GFP-Centrin	no signal
ENSG00000178462	ENST00000380419	TUBAL3	Diff. RPE1 GFP-Centrin	no signal
ENSG00000104804	ENST00000221399	TULP2	Diff. RPE1 GFP-Centrin	no signal
ENSG00000162543	ENST00000375099	UBXN10	Diff. RPE1 GFP-Centrin	no signal
ENSG00000075702	ENST00000401500	WDR62	Diff. RPE1 GFP-Centrin	no signal
ENSG00000143156	ENST00000367811	NME7	Diff. RPE1 GFP-Centrin	nuclear
ENSG00000151475	ENST00000281154	SLC25A31	Diff. RPE1 GFP-Centrin	nuclear
ENSG00000214021	ENST00000383827	TTLL3	Diff. RPE1 GFP-Centrin	nuclear
ENSG00000122257	ENST00000319715	RBBP6	Diff. RPE1 GFP-Centrin	perinuclear
ENSG00000117394	ENST00000426263	SLC2A1	Diff. RPE1 GFP-Centrin	Perinuclear
ENSG00000169504	ENST00000374379	CLIC4	Diff. RPE1 GFP-Centrin	Polarized
ENSG00000141367	ENST00000621829	CLTC	Diff. RPE1 GFP-Centrin	Polarized
ENSG00000109861	ENST00000227266	CTSC	Diff. RPE1 GFP-Centrin	Polarized
ENSG00000044574	ENST00000324460	HSPA5	Diff. RPE1 GFP-Centrin	Polarized
ENSG00000111057	ENST00000388835	KRT18	Diff. RPE1 GFP-Centrin	Polarized

ENSG00000168036	ENST00000349496	CTNNB1	Diff. RPE1 GFP-Centrin	Polarized, Foci
ENSG00000170004	ENST00000330494	CHD3	Diff. RPE1 GFP-Centrin	Protrusion
ENSG00000129250	ENST00000320785	KIF1C	Diff. RPE1 GFP-Centrin	Protrusion
ENSG00000170759	ENST00000302418	KIF5B	Diff. RPE1 GFP-Centrin	Protrusion
ENSG00000094914	ENST00000209873	AAAS	Diff. RPE1 GFP-Centrin	Random
ENSG00000275700	ENST00000619387	AATF	Diff. RPE1 GFP-Centrin	Random
ENSG00000165660	ENST00000298492	ABRAXAS2	Diff. RPE1 GFP-Centrin	Random
ENSG00000165660	ENST00000298492	ABRAXAS2	Diff. RPE1 GFP-Centrin	Random
ENSG00000113812	ENST00000335754	ACTR8	Diff. RPE1 GFP-Centrin	Random
ENSG00000113812	ENST00000335754	ACTR8	Diff. RPE1 GFP-Centrin	Random
ENSG00000084693	ENST00000323064	AGBL5	Diff. RPE1 GFP-Centrin	Random
ENSG00000135541	ENST00000367800	AHI1	Diff. RPE1 GFP-Centrin	Random
ENSG00000105127	ENST00000269701	AKAP8	Diff. RPE1 GFP-Centrin	Random
ENSG00000106948	ENST00000374088	AKNA	Diff. RPE1 GFP-Centrin	Random
ENSG00000106524	ENST00000306999	ANKMY2	Diff. RPE1 GFP-Centrin	Random
ENSG00000107890	ENST00000376070	ANKRD26	Diff. RPE1 GFP-Centrin	Random
ENSG00000165138	ENST00000353234	ANKS6	Diff. RPE1 GFP-Centrin	Random
ENSG00000135046	ENST00000257497	ANXA1	Diff. RPE1 GFP-Centrin	Random
ENSG00000122359	ENST00000422982	ANXA11	Diff. RPE1 GFP-Centrin	Random
ENSG00000182718	ENST00000451270	ANXA2	Diff. RPE1 GFP-Centrin	Random
ENSG00000134982	ENST00000257430	APC	Diff. RPE1 GFP-Centrin	Random
ENSG00000134982	ENST00000257430	APC	Diff. RPE1 GFP-Centrin	Random
ENSG00000100823	ENST00000216714	APEX1	Diff. RPE1 GFP-Centrin	Random
ENSG00000062725	ENST00000083182	APPBP2	Diff. RPE1 GFP-Centrin	Random
ENSG00000104728	ENST00000518288	ARHGEF10	Diff. RPE1 GFP-Centrin	Random
ENSG00000169379	ENST00000335438	ARL13B	Diff. RPE1 GFP-Centrin	Random
ENSG00000213465	ENST00000246747	ARL2	Diff. RPE1 GFP-Centrin	Random
ENSG00000102931	ENST00000219204	ARL2BP	Diff. RPE1 GFP-Centrin	Random
ENSG00000138175	ENST00000260746	ARL3	Diff. RPE1 GFP-Centrin	Random
ENSG00000113966	ENST00000463745	ARL6	Diff. RPE1 GFP-Centrin	Random
ENSG00000143862	ENST00000272217	ARL8A	Diff. RPE1 GFP-Centrin	Random
ENSG00000128272	ENST00000396680	ATF4	Diff. RPE1 GFP-Centrin	Random
ENSG00000169136	ENST00000423777	ATF5	Diff. RPE1 GFP-Centrin	Random
ENSG00000169136	ENST00000423777	ATF5	Diff. RPE1 GFP-Centrin	Random
ENSG00000159720	ENST00000290949	ATP6V0D1	Diff. RPE1 GFP-Centrin	Random
ENSG00000100554	ENST00000216442	ATP6V1D	Diff. RPE1 GFP-Centrin	Random
ENSG00000127423	ENST00000374298	AUNIP	Diff. RPE1 GFP-Centrin	Random
ENSG00000178999	ENST00000534871	AURKB	Diff. RPE1 GFP-Centrin	Random
ENSG00000168646	ENST00000307078	AXIN2	Diff. RPE1 GFP-Centrin	Random
ENSG00000108641	ENST00000261499	B9D1	Diff. RPE1 GFP-Centrin	Random
ENSG00000123810	ENST00000243578	B9D2	Diff. RPE1 GFP-Centrin	Random
ENSG00000175866	ENST00000428708	BAIAP2	Diff. RPE1 GFP-Centrin	Random
ENSG00000009954	ENST00000339594	BAZ1B	Diff. RPE1 GFP-Centrin	Random
ENSG00000119636	ENST00000394009	BBOF1	Diff. RPE1 GFP-Centrin	Random
ENSG00000174483	ENST00000318312	BBS1	Diff. RPE1 GFP-Centrin	Random
ENSG00000179941	ENST00000393262	BBS10	Diff. RPE1 GFP-Centrin	Random
ENSG00000181004	ENST00000314218	BBS12	Diff. RPE1 GFP-Centrin	Random
ENSG00000125124	ENST00000245157	BBS2	Diff. RPE1 GFP-Centrin	Random
ENSG00000140463	ENST00000268057	BBS4	Diff. RPE1 GFP-Centrin	Random
ENSG00000140463	ENST00000268057	BBS4	Diff. RPE1 GFP-Centrin	Random
ENSG00000163093	ENST00000295240	BBS5	Diff. RPE1 GFP-Centrin	Random
ENSG00000138686	ENST00000264499	BBS7	Diff. RPE1 GFP-Centrin	Random
ENSG00000122507	ENST00000242067	BBS9	Diff. RPE1 GFP-Centrin	Random

ENSG00000116752	ENST00000369541	BCAS2	Diff. RPE1 GFP-Centrin	Random
ENSG00000116752	ENST00000369541	BCAS2	Diff. RPE1 GFP-Centrin	Random
ENSG00000107949	ENST00000278100	BCCIP	Diff. RPE1 GFP-Centrin	Random
ENSG00000069399	ENST00000164227	BCL3	Diff. RPE1 GFP-Centrin	Random
ENSG00000115760	ENST00000421745	BIRC6	Diff. RPE1 GFP-Centrin	Random
ENSG00000145919	ENST00000311086	BOD1	Diff. RPE1 GFP-Centrin	Random
ENSG00000169679	ENST00000535254	BUB1	Diff. RPE1 GFP-Centrin	Random
ENSG00000156970	ENST00000287598	BUB1B	Diff. RPE1 GFP-Centrin	Random
ENSG00000154473	ENST00000368858	BUB3	Diff. RPE1 GFP-Centrin	Random
ENSG00000265590	ENST00000673807	C21ORF59	Diff. RPE1 GFP-Centrin	Random
ENSG00000198663	ENST00000480824	C6ORF89	Diff. RPE1 GFP-Centrin	Random
ENSG00000153790	ENST00000283905	C7ORF31	Diff. RPE1 GFP-Centrin	Random
ENSG00000183346	ENST00000330194	CABCOCO1	Diff. RPE1 GFP-Centrin	Random
ENSG00000154040	ENST00000327201	CABYR	Diff. RPE1 GFP-Centrin	Random
ENSG00000163888	ENST00000296238	CAMK2N2	Diff. RPE1 GFP-Centrin	Random
ENSG00000130559	ENST00000389532	CAMSAP1	Diff. RPE1 GFP-Centrin	Random
ENSG00000118200	ENST00000358823	CAMSAP2	Diff. RPE1 GFP-Centrin	Random
ENSG00000175294	ENST00000312106	CATSPER1	Diff. RPE1 GFP-Centrin	Random
ENSG00000105974	ENST00000405348	CAV1	Diff. RPE1 GFP-Centrin	Random
ENSG00000007080	ENST00000445755	CCDC124	Diff. RPE1 GFP-Centrin	Random
ENSG00000175455	ENST00000310351	CCDC14	Diff. RPE1 GFP-Centrin	Random
ENSG00000149548	ENST00000344762	CCDC15	Diff. RPE1 GFP-Centrin	Random
ENSG00000119242	ENST00000238156	CCDC92	Diff. RPE1 GFP-Centrin	Random
ENSG00000204536	ENST00000376266	CCHCR1	Diff. RPE1 GFP-Centrin	Random
ENSG00000162063	ENST00000397066	CCNF	Diff. RPE1 GFP-Centrin	Random
ENSG00000103540	ENST00000396208	CCP110	Diff. RPE1 GFP-Centrin	Random
ENSG00000103540	ENST00000396208	CCP110	Diff. RPE1 GFP-Centrin	Random
ENSG00000154429	ENST00000284617	CCSAP	Diff. RPE1 GFP-Centrin	Random
ENSG00000163468	ENST00000295688	CCT3	Diff. RPE1 GFP-Centrin	Random
ENSG00000115484	ENST00000544079	CCT4	Diff. RPE1 GFP-Centrin	Random
ENSG00000150753	ENST00000280326	CCT5	Diff. RPE1 GFP-Centrin	Random
ENSG00000150753	ENST00000503026	CCT5	Diff. RPE1 GFP-Centrin	Random
ENSG00000146731	ENST00000275603	CCT6A	Diff. RPE1 GFP-Centrin	Random
ENSG00000135624	ENST00000258091	CCT7	Diff. RPE1 GFP-Centrin	Random
ENSG00000156261	ENST00000286788	CCT8	Diff. RPE1 GFP-Centrin	Random
ENSG00000079335	ENST00000336454	CDC14A	Diff. RPE1 GFP-Centrin	Random
ENSG00000079335	ENST00000361544	CDC14A	Diff. RPE1 GFP-Centrin	Random
ENSG00000081377	ENST00000412285	CDC14B	Diff. RPE1 GFP-Centrin	Random
ENSG00000130177	ENST00000356221	CDC16	Diff. RPE1 GFP-Centrin	Random
ENSG00000130177	ENST00000360383	CDC16	Diff. RPE1 GFP-Centrin	Random
ENSG00000130177	ENST00000360383	CDC16	Diff. RPE1 GFP-Centrin	Random
ENSG00000117399	ENST00000310955	CDC20	Diff. RPE1 GFP-Centrin	Random
ENSG00000117399	ENST00000310955	CDC20	Diff. RPE1 GFP-Centrin	Random
ENSG00000004897	ENST00000531206	CDC27	Diff. RPE1 GFP-Centrin	Random
ENSG00000070831	ENST00000400259	CDC42	Diff. RPE1 GFP-Centrin	Random
ENSG00000097046	ENST00000234626	CDC7	Diff. RPE1 GFP-Centrin	Random
ENSG00000134690	ENST00000327331	CDCA8	Diff. RPE1 GFP-Centrin	Random
ENSG00000156345	ENST00000375871	CDK20	Diff. RPE1 GFP-Centrin	Random
ENSG00000167797	ENST00000301488	CDK2AP2	Diff. RPE1 GFP-Centrin	Random
ENSG00000164885	ENST00000485972	CDK5	Diff. RPE1 GFP-Centrin	Random
ENSG00000108465	ENST00000338399	CDK5RAP3	Diff. RPE1 GFP-Centrin	Random
ENSG00000105810	ENST00000265734	CDK6	Diff. RPE1 GFP-Centrin	Random
ENSG00000115163	ENST00000335756	CENPA	Diff. RPE1 GFP-Centrin	Random

ENSG00000138778	ENST00000380026	CENPE	Diff. RPE1 GFP-Centrin	Random
ENSG00000151849	ENST00000381884	CENPJ	Diff. RPE1 GFP-Centrin	Random
ENSG00000138092	ENST00000473706	CENPO	Diff. RPE1 GFP-Centrin	Random
ENSG00000175279	ENST00000477755	CENPS	Diff. RPE1 GFP-Centrin	Random
ENSG00000166582	ENST00000299736	CENPV	Diff. RPE1 GFP-Centrin	Random
ENSG00000203760	ENST00000368328	CENPW	Diff. RPE1 GFP-Centrin	Random
ENSG00000169689	ENST00000392359	CENPX	Diff. RPE1 GFP-Centrin	Random
ENSG00000116198	ENST00000378230	CEP104	Diff. RPE1 GFP-Centrin	Random
ENSG00000154240	ENST00000535342	CEP112	Diff. RPE1 GFP-Centrin	Random
ENSG00000168944	ENST00000306481	CEP120	Diff. RPE1 GFP-Centrin	Random
ENSG00000135315	ENST00000403245	CEP162	Diff. RPE1 GFP-Centrin	Random
ENSG00000135315	ENST00000403245	CEP162	Diff. RPE1 GFP-Centrin	Random
ENSG00000143702	ENST00000366542	CEP170	Diff. RPE1 GFP-Centrin	Random
ENSG00000099814	ENST00000556508	CEP170B	Diff. RPE1 GFP-Centrin	Random
ENSG00000174007	ENST00000409690	CEP19	Diff. RPE1 GFP-Centrin	Random
ENSG00000164118	ENST00000296519	CEP44	Diff. RPE1 GFP-Centrin	Random
ENSG00000182923	ENST00000332047	CEP63	Diff. RPE1 GFP-Centrin	Random
ENSG00000011523	ENST00000377990	CEP68	Diff. RPE1 GFP-Centrin	Random
ENSG00000173588	ENST00000339839	CEP83	Diff. RPE1 GFP-Centrin	Random
ENSG00000130695	ENST00000252992	CEP85	Diff. RPE1 GFP-Centrin	Random
ENSG00000121289	ENST00000305768	CEP89	Diff. RPE1 GFP-Centrin	Random
ENSG00000177143	ENST00000327228	CETN1	Diff. RPE1 GFP-Centrin	Random
ENSG00000147400	ENST00000370277	CETN2	Diff. RPE1 GFP-Centrin	Random
ENSG00000188596	ENST00000524981	CFAP54	Diff. RPE1 GFP-Centrin	Random
ENSG00000120051	ENST00000369704	CFAP58	Diff. RPE1 GFP-Centrin	Random
ENSG00000153774	ENST00000283882	CFDP1	Diff. RPE1 GFP-Centrin	Random
ENSG00000254505	ENST00000347519	CHMP4A	Diff. RPE1 GFP-Centrin	Random
ENSG00000101421	ENST00000217402	CHMP4B	Diff. RPE1 GFP-Centrin	Random
ENSG00000110172	ENST00000320585	CHORDC1	Diff. RPE1 GFP-Centrin	Random
ENSG00000110172	ENST00000457199	CHORDC1	Diff. RPE1 GFP-Centrin	Random
ENSG00000138433	ENST00000342016	CIR1	Diff. RPE1 GFP-Centrin	Random
ENSG00000136108	ENST00000378037	CKAP2	Diff. RPE1 GFP-Centrin	Random
ENSG00000130779	ENST00000620786	CLIP1	Diff. RPE1 GFP-Centrin	Random
ENSG00000106665	ENST00000361545	CLIP2	Diff. RPE1 GFP-Centrin	Random
ENSG00000070371	ENST00000442042	CLTCL1	Diff. RPE1 GFP-Centrin	Random
ENSG00000103351	ENST00000417763	CLUAP1	Diff. RPE1 GFP-Centrin	Random
ENSG00000044459	ENST00000380647	CNTLN	Diff. RPE1 GFP-Centrin	Random
ENSG00000170037	ENST00000563694	CNTROB	Diff. RPE1 GFP-Centrin	Random
ENSG00000099942	ENST00000354336	CRKL	Diff. RPE1 GFP-Centrin	Random
ENSG00000113712	ENST00000261798	CSNK1A1	Diff. RPE1 GFP-Centrin	Random
ENSG00000113712	ENST00000377843	CSNK1A1	Diff. RPE1 GFP-Centrin	Random
ENSG00000204435	ENST00000375882	CSNK2B	Diff. RPE1 GFP-Centrin	Random
ENSG00000104218	ENST00000262210	CSPP1	Diff. RPE1 GFP-Centrin	Random
ENSG00000060069	ENST00000613122	CTDP1	Diff. RPE1 GFP-Centrin	Random
ENSG00000198561	ENST00000526772	CTNND1	Diff. RPE1 GFP-Centrin	Random
ENSG00000044090	ENST00000265348	CUL7	Diff. RPE1 GFP-Centrin	Random
ENSG00000044090	ENST00000535468	CUL7	Diff. RPE1 GFP-Centrin	Random
ENSG00000205795	ENST00000381813	CYS1	Diff. RPE1 GFP-Centrin	Random
ENSG00000123977	ENST00000309931	DAW1	Diff. RPE1 GFP-Centrin	Random
ENSG00000198876	ENST00000361264	DCAF12	Diff. RPE1 GFP-Centrin	Random
ENSG00000164934	ENST00000297579	DCAF13	Diff. RPE1 GFP-Centrin	Random
ENSG00000164934	ENST00000297579	DCAF13	Diff. RPE1 GFP-Centrin	Random
ENSG00000118655	ENST00000369563	DCLRE1B	Diff. RPE1 GFP-Centrin	Random
ENSG00000137100	ENST00000259632	DCTN3	Diff. RPE1 GFP-Centrin	Random

ENSG00000132912	ENST00000447998	DCTN4	Diff. RPE1 GFP-Centrin	Random
ENSG00000104671	ENST00000221114	DCTN6	Diff. RPE1 GFP-Centrin	Random
ENSG00000135829	ENST00000367549	DHX9	Diff. RPE1 GFP-Centrin	Random
ENSG00000091140	ENST00000440410	DLD	Diff. RPE1 GFP-Centrin	Random
ENSG00000075711	ENST00000357674	DLG1	Diff. RPE1 GFP-Centrin	Random
ENSG00000256061	ENST00000457155	DNAAF4	Diff. RPE1 GFP-Centrin	Random
ENSG00000100246	ENST00000216068	DNAL4	Diff. RPE1 GFP-Centrin	Random
ENSG00000106976	ENST00000372923	DNM1	Diff. RPE1 GFP-Centrin	Random
ENSG00000087470	ENST00000452533	DNM1L	Diff. RPE1 GFP-Centrin	Random
ENSG00000133884	ENST00000252268	DPF2	Diff. RPE1 GFP-Centrin	Random
ENSG00000092964	ENST00000311151	DPYSL2	Diff. RPE1 GFP-Centrin	Random
ENSG00000185721	ENST00000331457	DRG1	Diff. RPE1 GFP-Centrin	Random
ENSG00000143476	ENST00000366991	DTL	Diff. RPE1 GFP-Centrin	Random
ENSG00000107404	ENST00000378891	DVL1	Diff. RPE1 GFP-Centrin	Random
ENSG00000144635	ENST00000273130	DYNC1LI1	Diff. RPE1 GFP-Centrin	Random
ENSG00000135720	ENST00000258198	DYNC1LI2	Diff. RPE1 GFP-Centrin	Random
ENSG00000138036	ENST00000260605	DYNC2LI1	Diff. RPE1 GFP-Centrin	Random
ENSG00000264364	ENST00000579991	DYNLL2	Diff. RPE1 GFP-Centrin	Random
ENSG00000125971	ENST00000357156	DYNLRB1	Diff. RPE1 GFP-Centrin	Random
ENSG00000165169	ENST00000378578	DYNLT3	Diff. RPE1 GFP-Centrin	Random
ENSG00000134874	ENST00000361396	DZIP1	Diff. RPE1 GFP-Centrin	Random
ENSG00000158163	ENST00000327532	DZIP1L	Diff. RPE1 GFP-Centrin	Random
ENSG00000101412	ENST00000343380	E2F1	Diff. RPE1 GFP-Centrin	Random
ENSG00000136813	ENST00000259335	ECPAS	Diff. RPE1 GFP-Centrin	Random
ENSG00000203965	ENST00000371088	EFCAB7	Diff. RPE1 GFP-Centrin	Random
ENSG00000096093	ENST00000371068	EFHC1	Diff. RPE1 GFP-Centrin	Random
ENSG00000013016	ENST00000322054	EHD3	Diff. RPE1 GFP-Centrin	Random
ENSG00000102119	ENST00000369842	EMD	Diff. RPE1 GFP-Centrin	Random
ENSG00000125746	ENST00000245925	EML2	Diff. RPE1 GFP-Centrin	Random
ENSG00000186871	ENST00000334463	ERCC6L	Diff. RPE1 GFP-Centrin	Random
ENSG00000186871	ENST00000334463	ERCC6L	Diff. RPE1 GFP-Centrin	Random
ENSG00000100632	ENST00000557016	ERH	Diff. RPE1 GFP-Centrin	Random
ENSG00000135476	ENST00000257934	ESPL1	Diff. RPE1 GFP-Centrin	Random
ENSG00000072840	ENST00000382674	EVC	Diff. RPE1 GFP-Centrin	Random
ENSG00000180104	ENST00000512944	EXOC3	Diff. RPE1 GFP-Centrin	Random
ENSG00000158161	ENST00000436342	EYA3	Diff. RPE1 GFP-Centrin	Random
ENSG00000158161	ENST00000436342	EYA3	Diff. RPE1 GFP-Centrin	Random
ENSG00000170264	ENST00000405894	FAM161A	Diff. RPE1 GFP-Centrin	Random
ENSG00000153310	ENST00000517654	FAM49B	Diff. RPE1 GFP-Centrin	Random
ENSG00000143756	ENST00000366862	FBXO28	Diff. RPE1 GFP-Centrin	Random
ENSG00000103264	ENST00000565593	FBXO31	Diff. RPE1 GFP-Centrin	Random
ENSG00000149557	ENST00000278919	FEZ1	Diff. RPE1 GFP-Centrin	Random
ENSG00000004478	ENST00000001008	FKBP4	Diff. RPE1 GFP-Centrin	Random
ENSG00000154803	ENST00000285071	FLCN	Diff. RPE1 GFP-Centrin	Random
ENSG00000154803	ENST00000285071	FLCN	Diff. RPE1 GFP-Centrin	Random
ENSG00000133393	ENST00000255759	FOPNL	Diff. RPE1 GFP-Centrin	Random
ENSG00000163820	ENST00000296137	FYCO1	Diff. RPE1 GFP-Centrin	Random
ENSG00000163820	ENST00000433878	FYCO1	Diff. RPE1 GFP-Centrin	Random
ENSG00000139112	ENST00000266458	GABARAPL1	Diff. RPE1 GFP-Centrin	Random
ENSG00000141013	ENST00000268699	GAS8	Diff. RPE1 GFP-Centrin	Random
ENSG00000178295	ENST00000381254	GEN1	Diff. RPE1 GFP-Centrin	Random
ENSG00000074047	ENST00000361492	GLI2	Diff. RPE1 GFP-Centrin	Random
ENSG00000106571	ENST00000395925	GLI3	Diff. RPE1 GFP-Centrin	Random

ENSG00000127955	ENST00000351004	GNAI1	Diff. RPE1 GFP-Centrin	Random
ENSG00000127955	ENST00000351004	GNAI1	Diff. RPE1 GFP-Centrin	Random
ENSG00000114353	ENST00000266027	GNAI2	Diff. RPE1 GFP-Centrin	Random
ENSG00000167110	ENST00000421699	GOLGA2	Diff. RPE1 GFP-Centrin	Random
ENSG00000143147	ENST00000271357	GPR161	Diff. RPE1 GFP-Centrin	Random
ENSG00000143147	ENST00000271357	GPR161	Diff. RPE1 GFP-Centrin	Random
ENSG00000173020	ENST00000308595	GRK2	Diff. RPE1 GFP-Centrin	Random
ENSG00000082701	ENST00000264235	GSK3B	Diff. RPE1 GFP-Centrin	Random
ENSG00000188486	ENST00000530167	H2AFX	Diff. RPE1 GFP-Centrin	Random
ENSG00000113648	ENST00000312469	H2AFY	Diff. RPE1 GFP-Centrin	Random
ENSG00000177602	ENST00000325418	HASPIN	Diff. RPE1 GFP-Centrin	Random
ENSG00000152240	ENST00000282058	HAUS1	Diff. RPE1 GFP-Centrin	Random
ENSG00000214367	ENST00000443786	HAUS3	Diff. RPE1 GFP-Centrin	Random
ENSG00000214367	ENST00000443786	HAUS3	Diff. RPE1 GFP-Centrin	Random
ENSG00000092036	ENST00000541587	HAUS4	Diff. RPE1 GFP-Centrin	Random
ENSG00000249115	ENST00000203166	HAUS5	Diff. RPE1 GFP-Centrin	Random
ENSG00000147874	ENST00000380502	HAUS6	Diff. RPE1 GFP-Centrin	Random
ENSG00000147874	ENST00000380502	HAUS6	Diff. RPE1 GFP-Centrin	Random
ENSG00000213397	ENST00000370211	HAUS7	Diff. RPE1 GFP-Centrin	Random
ENSG00000213397	ENST00000370211	HAUS7	Diff. RPE1 GFP-Centrin	Random
ENSG00000128731	ENST00000261609	HERC2	Diff. RPE1 GFP-Centrin	Random
ENSG00000156515	ENST00000359426	HK1	Diff. RPE1 GFP-Centrin	Random
ENSG00000095066	ENST00000264827	HOOK2	Diff. RPE1 GFP-Centrin	Random
ENSG00000168172	ENST00000307602	HOOK3	Diff. RPE1 GFP-Centrin	Random
ENSG00000185122	ENST00000528838	HSF1	Diff. RPE1 GFP-Centrin	Random
ENSG00000204389	ENST00000375651	HSPA1A	Diff. RPE1 GFP-Centrin	Random
ENSG00000204388	ENST00000375650	HSPA1B	Diff. RPE1 GFP-Centrin	Random
ENSG00000173110	ENST00000309758	HSPA6	Diff. RPE1 GFP-Centrin	Random
ENSG00000081870	ENST00000194214	HSPB11	Diff. RPE1 GFP-Centrin	Random
ENSG00000197386	ENST00000355072	HTT	Diff. RPE1 GFP-Centrin	Random
ENSG00000112144	ENST00000350082	ICK	Diff. RPE1 GFP-Centrin	Random
ENSG00000187535	ENST00000426508	IFT140	Diff. RPE1 GFP-Centrin	Random
ENSG00000138002	ENST00000260570	IFT172	Diff. RPE1 GFP-Centrin	Random
ENSG00000109083	ENST00000395418	IFT20	Diff. RPE1 GFP-Centrin	Random
ENSG00000128581	ENST00000315322	IFT22	Diff. RPE1 GFP-Centrin	Random
ENSG00000100360	ENST00000433985	IFT27	Diff. RPE1 GFP-Centrin	Random
ENSG00000100360	ENST00000433985	IFT27	Diff. RPE1 GFP-Centrin	Random
ENSG00000118096	ENST00000264021	IFT46	Diff. RPE1 GFP-Centrin	Random
ENSG00000101052	ENST00000373039	IFT52	Diff. RPE1 GFP-Centrin	Random
ENSG00000114446	ENST00000264538	IFT57	Diff. RPE1 GFP-Centrin	Random
ENSG00000096872	ENST00000380062	IFT74	Diff. RPE1 GFP-Centrin	Random
ENSG00000122970	ENST00000552912	IFT81	Diff. RPE1 GFP-Centrin	Random
ENSG00000032742	ENST00000351808	IFT88	Diff. RPE1 GFP-Centrin	Random
ENSG00000128908	ENST00000361937	INO80	Diff. RPE1 GFP-Centrin	Random
ENSG00000148384	ENST00000371712	INPP5E	Diff. RPE1 GFP-Centrin	Random
ENSG00000173226	ENST00000310864	IQCB1	Diff. RPE1 GFP-Centrin	Random
ENSG00000106012	ENST00000402050	IQCE	Diff. RPE1 GFP-Centrin	Random
ENSG00000106012	ENST00000402050	IQCE	Diff. RPE1 GFP-Centrin	Random
ENSG00000114473	ENST00000265239	IQCG	Diff. RPE1 GFP-Centrin	Random
ENSG00000140575	ENST00000268182	IQGAP1	Diff. RPE1 GFP-Centrin	Random
ENSG00000142856	ENST00000271002	ITGB3BP	Diff. RPE1 GFP-Centrin	Random
ENSG00000143543	ENST00000271843	JTB	Diff. RPE1 GFP-Centrin	Random
ENSG00000108773	ENST00000225916	KAT2A	Diff. RPE1 GFP-Centrin	Random

ENSG00000103510	ENST00000219797	KAT8	Diff. RPE1 GFP-Centrin	Random
ENSG00000186625	ENST00000367411	KATNA1	Diff. RPE1 GFP-Centrin	Random
ENSG00000186625	ENST00000367411	KATNA1	Diff. RPE1 GFP-Centrin	Random
ENSG00000102781	ENST00000380615	KATNAL1	Diff. RPE1 GFP-Centrin	Random
ENSG00000140854	ENST00000379661	KATNB1	Diff. RPE1 GFP-Centrin	Random
ENSG00000198920	ENST00000361413	KIAA0753	Diff. RPE1 GFP-Centrin	Random
ENSG00000197892	ENST00000524189	KIF13B	Diff. RPE1 GFP-Centrin	Random
ENSG00000197892	ENST00000524189	KIF13B	Diff. RPE1 GFP-Centrin	Random
ENSG00000163808	ENST00000326047	KIF15	Diff. RPE1 GFP-Centrin	Random
ENSG00000137807	ENST00000260363	KIF23	Diff. RPE1 GFP-Centrin	Random
ENSG00000068796	ENST00000381103	KIF2A	Diff. RPE1 GFP-Centrin	Random
ENSG00000068796	ENST00000401507	KIF2A	Diff. RPE1 GFP-Centrin	Random
ENSG00000101350	ENST00000375712	KIF3B	Diff. RPE1 GFP-Centrin	Random
ENSG00000101350	ENST00000375712	KIF3B	Diff. RPE1 GFP-Centrin	Random
ENSG00000090889	ENST00000374403	KIF4A	Diff. RPE1 GFP-Centrin	Random
ENSG00000166813	ENST00000394412	KIF7	Diff. RPE1 GFP-Centrin	Random
ENSG00000075945	ENST00000361580	KIFAP3	Diff. RPE1 GFP-Centrin	Random
ENSG00000137171	ENST00000347162	KLC4	Diff. RPE1 GFP-Centrin	Random
ENSG00000003096	ENST00000371882	KLHL13	Diff. RPE1 GFP-Centrin	Random
ENSG00000162413	ENST00000377658	KLHL21	Diff. RPE1 GFP-Centrin	Random
ENSG00000162413	ENST00000377658	KLHL21	Diff. RPE1 GFP-Centrin	Random
ENSG00000099910	ENST00000328879	KLHL22	Diff. RPE1 GFP-Centrin	Random
ENSG00000198642	ENST00000359039	KLHL9	Diff. RPE1 GFP-Centrin	Random
ENSG00000128944	ENST00000608100	KNSTRN	Diff. RPE1 GFP-Centrin	Random
ENSG00000184445	ENST00000333479	KNTC1	Diff. RPE1 GFP-Centrin	Random
ENSG00000135338	ENST00000369846	LCA5	Diff. RPE1 GFP-Centrin	Random
ENSG00000166477	ENST00000299601	LEO1	Diff. RPE1 GFP-Centrin	Random
ENSG00000169683	ENST00000306688	LRRC45	Diff. RPE1 GFP-Centrin	Random
ENSG00000133739	ENST00000360375	LRRCC1	Diff. RPE1 GFP-Centrin	Random
ENSG00000133739	ENST00000360375	LRRCC1	Diff. RPE1 GFP-Centrin	Random
ENSG00000107816	ENST00000370223	LZTS2	Diff. RPE1 GFP-Centrin	Random
ENSG00000107816	ENST00000370223	LZTS2	Diff. RPE1 GFP-Centrin	Random
ENSG00000002822	ENST00000399654	MAD1L1	Diff. RPE1 GFP-Centrin	Random
ENSG00000002822	ENST00000399654	MAD1L1	Diff. RPE1 GFP-Centrin	Random
ENSG00000179632	ENST00000322428	MAF1	Diff. RPE1 GFP-Centrin	Random
ENSG00000130479	ENST00000324096	MAP1S	Diff. RPE1 GFP-Centrin	Random
ENSG00000126934	ENST00000262948	MAP2K2	Diff. RPE1 GFP-Centrin	Random
ENSG00000047849	ENST00000429422	MAP4	Diff. RPE1 GFP-Centrin	Random
ENSG00000101367	ENST00000375571	MAPRE1	Diff. RPE1 GFP-Centrin	Random
ENSG00000166974	ENST00000300249	MAPRE2	Diff. RPE1 GFP-Centrin	Random
ENSG00000084764	ENST00000233121	MAPRE3	Diff. RPE1 GFP-Centrin	Random
ENSG00000007047	ENST00000262891	MARK4	Diff. RPE1 GFP-Centrin	Random
ENSG00000152601	ENST00000463374	MBNL1	Diff. RPE1 GFP-Centrin	Random
ENSG00000163875	ENST00000296214	MEAF6	Diff. RPE1 GFP-Centrin	Random
ENSG00000169057	ENST00000303391	MECP2	Diff. RPE1 GFP-Centrin	Random
ENSG00000184634	ENST00000374080	MED12	Diff. RPE1 GFP-Centrin	Random
ENSG00000101752	ENST00000261537	MIB1	Diff. RPE1 GFP-Centrin	Random
ENSG00000101871	ENST00000317552	MID1	Diff. RPE1 GFP-Centrin	Random
ENSG00000125863	ENST00000399054	MKKS	Diff. RPE1 GFP-Centrin	Random
ENSG00000011143	ENST00000393119	MKS1	Diff. RPE1 GFP-Centrin	Random
ENSG00000011143	ENST00000393119	MKS1	Diff. RPE1 GFP-Centrin	Random
ENSG00000076242	ENST00000231790	MLH1	Diff. RPE1 GFP-Centrin	Random
ENSG00000168303	ENST00000306984	MPLKIP	Diff. RPE1 GFP-Centrin	Random

ENSG00000172167	ENST00000305949	MTBP	Diff. RPE1 GFP-Centrin	Random
ENSG00000133026	ENST00000269243	MYH10	Diff. RPE1 GFP-Centrin	Random
ENSG00000204899	ENST00000377818	MZT1	Diff. RPE1 GFP-Centrin	Random
ENSG00000135372	ENST00000257829	NAT10	Diff. RPE1 GFP-Centrin	Random
ENSG00000163382	ENST00000368235	NAXE	Diff. RPE1 GFP-Centrin	Random
ENSG00000010292	ENST00000315579	NCAPD2	Diff. RPE1 GFP-Centrin	Random
ENSG00000109805	ENST00000251496	NCAPG	Diff. RPE1 GFP-Centrin	Random
ENSG00000167566	ENST00000335999	NCKAP5L	Diff. RPE1 GFP-Centrin	Random
ENSG00000166579	ENST00000334527	NDEL1	Diff. RPE1 GFP-Centrin	Random
ENSG00000166579	ENST00000402554	NDEL1	Diff. RPE1 GFP-Centrin	Random
ENSG00000104419	ENST00000323851	NDRG1	Diff. RPE1 GFP-Centrin	Random
ENSG00000137601	ENST00000507142	NEK1	Diff. RPE1 GFP-Centrin	Random
ENSG00000137601	ENST00000511633	NEK1	Diff. RPE1 GFP-Centrin	Random
ENSG00000117650	ENST00000366999	NEK2	Diff. RPE1 GFP-Centrin	Random
ENSG00000136098	ENST00000610828	NEK3	Diff. RPE1 GFP-Centrin	Random
ENSG00000114904	ENST00000233027	NEK4	Diff. RPE1 GFP-Centrin	Random
ENSG00000119408	ENST00000320246	NEK6	Diff. RPE1 GFP-Centrin	Random
ENSG00000151414	ENST00000367385	NEK7	Diff. RPE1 GFP-Centrin	Random
ENSG00000119638	ENST00000238616	NEK9	Diff. RPE1 GFP-Centrin	Random
ENSG00000119638	ENST00000238616	NEK9	Diff. RPE1 GFP-Centrin	Random
ENSG00000196712	ENST00000358273	NF1	Diff. RPE1 GFP-Centrin	Random
ENSG00000116044	ENST00000397063	NFE2L2	Diff. RPE1 GFP-Centrin	Random
ENSG00000100906	ENST00000216797	NFKBIA	Diff. RPE1 GFP-Centrin	Random
ENSG00000145029	ENST00000273598	NICN1	Diff. RPE1 GFP-Centrin	Random
ENSG00000239672	ENST00000393196	NME1	Diff. RPE1 GFP-Centrin	Random
ENSG00000144061	ENST00000445609	NPHP1	Diff. RPE1 GFP-Centrin	Random
ENSG00000113971	ENST00000337331	NPHP3	Diff. RPE1 GFP-Centrin	Random
ENSG00000131697	ENST00000378156	NPHP4	Diff. RPE1 GFP-Centrin	Random
ENSG00000181163	ENST00000296930	NPM1	Diff. RPE1 GFP-Centrin	Random
ENSG00000181163	ENST00000517671	NPM1	Diff. RPE1 GFP-Centrin	Random
ENSG00000117697	ENST00000422588	NSL1	Diff. RPE1 GFP-Centrin	Random
ENSG00000103274	ENST00000283027	NUBP1	Diff. RPE1 GFP-Centrin	Random
ENSG00000095906	ENST00000262302	NUBP2	Diff. RPE1 GFP-Centrin	Random
ENSG00000090273	ENST00000321265	NUDC	Diff. RPE1 GFP-Centrin	Random
ENSG00000167005	ENST00000300291	NUDT21	Diff. RPE1 GFP-Centrin	Random
ENSG00000111581	ENST00000229179	NUP107	Diff. RPE1 GFP-Centrin	Random
ENSG00000069248	ENST00000261396	NUP133	Diff. RPE1 GFP-Centrin	Random
ENSG00000125450	ENST00000245544	NUP85	Diff. RPE1 GFP-Centrin	Random
ENSG00000137804	ENST00000414849	NUSAP1	Diff. RPE1 GFP-Centrin	Random
ENSG00000124006	ENST00000404537	OBSL1	Diff. RPE1 GFP-Centrin	Random
ENSG00000122126	ENST00000357121	OCRL	Diff. RPE1 GFP-Centrin	Random
ENSG00000138430	ENST00000344357	OLA1	Diff. RPE1 GFP-Centrin	Random
ENSG00000115942	ENST00000234296	ORC2	Diff. RPE1 GFP-Centrin	Random
ENSG00000102981	ENST00000458121	PARD6A	Diff. RPE1 GFP-Centrin	Random
ENSG00000102981	ENST00000458121	PARD6A	Diff. RPE1 GFP-Centrin	Random
ENSG00000041880	ENST00000398755	PARP3	Diff. RPE1 GFP-Centrin	Random
ENSG00000132849	ENST00000371158	PATJ	Diff. RPE1 GFP-Centrin	Random
ENSG00000166803	ENST00000559519	PCLAF	Diff. RPE1 GFP-Centrin	Random
ENSG00000078674	ENST00000325083	PCM1	Diff. RPE1 GFP-Centrin	Random
ENSG00000132646	ENST00000379143	PCNA	Diff. RPE1 GFP-Centrin	Random
ENSG00000154678	ENST00000396193	PDE1C	Diff. RPE1 GFP-Centrin	Random
ENSG00000113448	ENST00000340635	PDE4D	Diff. RPE1 GFP-Centrin	Random
ENSG00000156973	ENST00000287600	PDE6D	Diff. RPE1 GFP-Centrin	Random

ENSG00000241360	ENST00000215904	PDXP	Diff. RPE1 GFP-Centrin	Random
ENSG00000100029	ENST00000354694	PES1	Diff. RPE1 GFP-Centrin	Random
ENSG00000170950	ENST00000304801	PGK2	Diff. RPE1 GFP-Centrin	Random
ENSG00000164902	ENST00000297540	PHAX	Diff. RPE1 GFP-Centrin	Random
ENSG00000197724	ENST00000359246	PHF2	Diff. RPE1 GFP-Centrin	Random
ENSG00000083535	ENST00000326291	PIBF1	Diff. RPE1 GFP-Centrin	Random
ENSG00000051382	ENST00000477593	PIK3CB	Diff. RPE1 GFP-Centrin	Random
ENSG00000127445	ENST00000247970	PIN1	Diff. RPE1 GFP-Centrin	Random
ENSG00000254093	ENST00000519088	PINX1	Diff. RPE1 GFP-Centrin	Random
ENSG00000110697	ENST00000534749	PITPNM1	Diff. RPE1 GFP-Centrin	Random
ENSG00000118762	ENST00000237596	PKD2	Diff. RPE1 GFP-Centrin	Random
ENSG00000067225	ENST00000335181	PKM	Diff. RPE1 GFP-Centrin	Random
ENSG00000145632	ENST00000274289	PLK2	Diff. RPE1 GFP-Centrin	Random
ENSG00000173846	ENST00000372201	PLK3	Diff. RPE1 GFP-Centrin	Random
ENSG00000164087	ENST00000296484	POC1A	Diff. RPE1 GFP-Centrin	Random
ENSG00000139323	ENST00000313546	POC1B	Diff. RPE1 GFP-Centrin	Random
ENSG00000152359	ENST00000428202	POC5	Diff. RPE1 GFP-Centrin	Random
ENSG00000070501	ENST00000265421	POLB	Diff. RPE1 GFP-Centrin	Random
ENSG00000100413	ENST00000355209	POLR3H	Diff. RPE1 GFP-Centrin	Random
ENSG00000066027	ENST00000261461	PPP2R5A	Diff. RPE1 GFP-Centrin	Random
ENSG00000163605	ENST00000356692	PPP4R2	Diff. RPE1 GFP-Centrin	Random
ENSG00000198901	ENST00000556972	PRC1	Diff. RPE1 GFP-Centrin	Random
ENSG00000142875	ENST00000370689	PRKACB	Diff. RPE1 GFP-Centrin	Random
ENSG00000114302	ENST00000265563	PRKAR2A	Diff. RPE1 GFP-Centrin	Random
ENSG00000005249	ENST00000265717	PRKAR2B	Diff. RPE1 GFP-Centrin	Random
ENSG00000101000	ENST00000216968	PROCR	Diff. RPE1 GFP-Centrin	Random
ENSG00000080815	ENST00000357710	PSEN1	Diff. RPE1 GFP-Centrin	Random
ENSG00000143801	ENST00000366783	PSEN2	Diff. RPE1 GFP-Centrin	Random
ENSG00000129084	ENST00000396393	PSMA1	Diff. RPE1 GFP-Centrin	Random
ENSG00000100804	ENST00000361611	PSMB5	Diff. RPE1 GFP-Centrin	Random
ENSG00000134222	ENST00000369909	PSRC1	Diff. RPE1 GFP-Centrin	Random
ENSG00000185920	ENST00000546744	PTCH1	Diff. RPE1 GFP-Centrin	Random
ENSG00000076201	ENST00000265562	PTPN23	Diff. RPE1 GFP-Centrin	Random
ENSG00000084733	ENST00000264710	RAB10	Diff. RPE1 GFP-Centrin	Random
ENSG00000103769	ENST00000564910	RAB11A	Diff. RPE1 GFP-Centrin	Random
ENSG00000112210	ENST00000317483	RAB23	Diff. RPE1 GFP-Centrin	Random
ENSG00000222014	ENST00000410061	RAB6C	Diff. RPE1 GFP-Centrin	Random
ENSG00000167461	ENST00000300935	RAB8A	Diff. RPE1 GFP-Centrin	Random
ENSG00000011454	ENST00000373647	RABGAP1	Diff. RPE1 GFP-Centrin	Random
ENSG00000011454	ENST00000373647	RABGAP1	Diff. RPE1 GFP-Centrin	Random
ENSG00000152061	ENST00000251507	RABGAP1L	Diff. RPE1 GFP-Centrin	Random
ENSG00000161800	ENST00000312377	RACGAP1	Diff. RPE1 GFP-Centrin	Random
ENSG00000204628	ENST00000512805	RACK1	Diff. RPE1 GFP-Centrin	Random
ENSG00000051180	ENST00000267868	RAD51	Diff. RPE1 GFP-Centrin	Random
ENSG00000185379	ENST00000335858	RAD51D	Diff. RPE1 GFP-Centrin	Random
ENSG00000132341	ENST00000254675	RAN	Diff. RPE1 GFP-Centrin	Random
ENSG00000099901	ENST00000331821	RANBP1	Diff. RPE1 GFP-Centrin	Random
ENSG00000153201	ENST00000283195	RANBP2	Diff. RPE1 GFP-Centrin	Random
ENSG00000031823	ENST00000439268	RANBP3	Diff. RPE1 GFP-Centrin	Random
ENSG00000100401	ENST00000356244	RANGAP1	Diff. RPE1 GFP-Centrin	Random
ENSG00000125249	ENST00000245304	RAP2A	Diff. RPE1 GFP-Centrin	Random
ENSG00000158987	ENST00000509018	RAPGEF6	Diff. RPE1 GFP-Centrin	Random
ENSG00000179051	ENST00000375436	RCC2	Diff. RPE1 GFP-Centrin	Random

ENSG00000137710	ENST00000343115	RDX	Diff. RPE1 GFP-Centrin	Random
ENSG00000165476	ENST00000373758	REEP3	Diff. RPE1 GFP-Centrin	Random
ENSG00000168476	ENST00000306306	REEP4	Diff. RPE1 GFP-Centrin	Random
ENSG00000104856	ENST00000221452	RELB	Diff. RPE1 GFP-Centrin	Random
ENSG00000196862	ENST00000408999	RGPD4	Diff. RPE1 GFP-Centrin	Random
ENSG00000111785	ENST00000392837	RIC8B	Diff. RPE1 GFP-Centrin	Random
ENSG00000080345	ENST00000243326	RIF1	Diff. RPE1 GFP-Centrin	Random
ENSG00000188026	ENST00000376874	RILPL1	Diff. RPE1 GFP-Centrin	Random
ENSG00000139405	ENST00000548278	RITA1	Diff. RPE1 GFP-Centrin	Random
ENSG00000176623	ENST00000406452	RMDN1	Diff. RPE1 GFP-Centrin	Random
ENSG00000137824	ENST00000338376	RMDN3	Diff. RPE1 GFP-Centrin	Random
ENSG00000112130	ENST00000229866	RNF8	Diff. RPE1 GFP-Centrin	Random
ENSG00000134318	ENST00000315872	ROCK2	Diff. RPE1 GFP-Centrin	Random
ENSG00000101413	ENST00000373433	RPRD1B	Diff. RPE1 GFP-Centrin	Random
ENSG00000149273	ENST00000524851	RPS3	Diff. RPE1 GFP-Centrin	Random
ENSG00000171863	ENST00000304921	RPS7	Diff. RPE1 GFP-Centrin	Random
ENSG00000172426	ENST00000372163	RSPH9	Diff. RPE1 GFP-Centrin	Random
ENSG00000087302	ENST00000261700	RTRAF	Diff. RPE1 GFP-Centrin	Random
ENSG00000183207	ENST00000596247	RUVBL2	Diff. RPE1 GFP-Centrin	Random
ENSG00000156876	ENST00000287482	SASS6	Diff. RPE1 GFP-Centrin	Random
ENSG00000143653	ENST00000366510	SCCPDH	Diff. RPE1 GFP-Centrin	Random
ENSG00000151466	ENST00000281142	SCLT1	Diff. RPE1 GFP-Centrin	Random
ENSG00000111319	ENST00000228916	SCNN1A	Diff. RPE1 GFP-Centrin	Random
ENSG00000157020	ENST00000350697	SEC13	Diff. RPE1 GFP-Centrin	Random
ENSG00000085415	ENST00000262124	SEH1L	Diff. RPE1 GFP-Centrin	Random
ENSG00000129810	ENST00000442720	SGO1	Diff. RPE1 GFP-Centrin	Random
ENSG00000138771	ENST00000296043	SHROOM3	Diff. RPE1 GFP-Centrin	Random
ENSG00000068903	ENST00000249396	SIRT2	Diff. RPE1 GFP-Centrin	Random
ENSG00000182628	ENST00000330137	SKA2	Diff. RPE1 GFP-Centrin	Random
ENSG00000157933	ENST00000378536	SKI	Diff. RPE1 GFP-Centrin	Random
ENSG00000155380	ENST00000369626	SLC16A1	Diff. RPE1 GFP-Centrin	Random
ENSG00000133302	ENST00000265140	SLF1	Diff. RPE1 GFP-Centrin	Random
ENSG00000141646	ENST00000342988	SMAD4	Diff. RPE1 GFP-Centrin	Random
ENSG00000101665	ENST00000262158	SMAD7	Diff. RPE1 GFP-Centrin	Random
ENSG00000153147	ENST00000283131	SMARCA5	Diff. RPE1 GFP-Centrin	Random
ENSG00000128602	ENST00000249373	SMO	Diff. RPE1 GFP-Centrin	Random
ENSG00000099940	ENST00000215730	SNAP29	Diff. RPE1 GFP-Centrin	Random
ENSG00000064199	ENST00000227135	SPA17	Diff. RPE1 GFP-Centrin	Random
ENSG00000144451	ENST00000432529	SPAG16	Diff. RPE1 GFP-Centrin	Random
ENSG00000061656	ENST00000374273	SPAG4	Diff. RPE1 GFP-Centrin	Random
ENSG00000076382	ENST00000321765	SPAG5	Diff. RPE1 GFP-Centrin	Random
ENSG00000133104	ENST00000494062	SPART	Diff. RPE1 GFP-Centrin	Random
ENSG00000021574	ENST00000615843	SPAST	Diff. RPE1 GFP-Centrin	Random
ENSG00000163611	ENST00000295872	SPICE1	Diff. RPE1 GFP-Centrin	Random
ENSG00000198917	ENST00000361256	SPOUT1	Diff. RPE1 GFP-Centrin	Random
ENSG00000084112	ENST00000360239	SSH1	Diff. RPE1 GFP-Centrin	Random
ENSG00000176101	ENST00000322310	SSNA1	Diff. RPE1 GFP-Centrin	Random
ENSG00000159433	ENST00000290607	STARD9	Diff. RPE1 GFP-Centrin	Random
ENSG00000040341	ENST00000355780	STAU2	Diff. RPE1 GFP-Centrin	Random
ENSG00000117632	ENST00000374291	STMN1	Diff. RPE1 GFP-Centrin	Random
ENSG00000159082	ENST00000438952	SYNJ1	Diff. RPE1 GFP-Centrin	Random
ENSG00000147526	ENST00000348567	TACC1	Diff. RPE1 GFP-Centrin	Random
ENSG00000171148	ENST00000301964	TADA3	Diff. RPE1 GFP-Centrin	Random

ENSG00000169762	ENST00000405303	TAPT1	Diff. RPE1 GFP-Centrin	Random
ENSG00000127364	ENST00000247881	TAS2R4	Diff. RPE1 GFP-Centrin	Random
ENSG00000156787	ENST00000518805	TBC1D31	Diff. RPE1 GFP-Centrin	Random
ENSG00000146350	ENST00000398197	TBC1D32	Diff. RPE1 GFP-Centrin	Random
ENSG00000141556	ENST00000355528	TBCD	Diff. RPE1 GFP-Centrin	Random
ENSG00000284770	ENST00000366601	TBCE	Diff. RPE1 GFP-Centrin	Random
ENSG00000139437	ENST00000405876	TCHP	Diff. RPE1 GFP-Centrin	Random
ENSG00000120438	ENST00000321394	TCP1	Diff. RPE1 GFP-Centrin	Random
ENSG00000179029	ENST00000437139	TMEM107	Diff. RPE1 GFP-Centrin	Random
ENSG00000186889	ENST00000335390	TMEM17	Diff. RPE1 GFP-Centrin	Random
ENSG00000150433	ENST00000531262	TMEM218	Diff. RPE1 GFP-Centrin	Random
ENSG00000155755	ENST00000286196	TMEM237	Diff. RPE1 GFP-Centrin	Random
ENSG00000164953	ENST00000453321	TMEM67	Diff. RPE1 GFP-Centrin	Random
ENSG00000173273	ENST00000310430	TNKS	Diff. RPE1 GFP-Centrin	Random
ENSG00000107854	ENST00000371627	TNKS2	Diff. RPE1 GFP-Centrin	Random
ENSG00000083312	ENST00000454282	TNPO1	Diff. RPE1 GFP-Centrin	Random
ENSG00000198718	ENST00000361462	TOGARAM1	Diff. RPE1 GFP-Centrin	Random
ENSG00000197579	ENST00000360538	TOPORS	Diff. RPE1 GFP-Centrin	Random
ENSG00000134779	ENST00000383056	TPGS2	Diff. RPE1 GFP-Centrin	Random
ENSG00000204104	ENST00000391993	TRAF3IP1	Diff. RPE1 GFP-Centrin	Random
ENSG00000213186	ENST00000309784	TRIM59	Diff. RPE1 GFP-Centrin	Random
ENSG00000100106	ENST00000403663	TRIOBP	Diff. RPE1 GFP-Centrin	Random
ENSG00000100815	ENST00000267622	TRIP11	Diff. RPE1 GFP-Centrin	Random
ENSG00000103671	ENST00000261884	TRIP4	Diff. RPE1 GFP-Centrin	Random
ENSG00000128881	ENST00000267890	TTBK2	Diff. RPE1 GFP-Centrin	Random
ENSG00000149292	ENST00000529221	TTC12	Diff. RPE1 GFP-Centrin	Random
ENSG00000011295	ENST00000475723	TTC19	Diff. RPE1 GFP-Centrin	Random
ENSG00000123607	ENST00000243344	TTC21B	Diff. RPE1 GFP-Centrin	Random
ENSG00000105948	ENST00000464848	TTC26	Diff. RPE1 GFP-Centrin	Random
ENSG00000100154	ENST00000397906	TTC28	Diff. RPE1 GFP-Centrin	Random
ENSG00000197557	ENST00000355689	TTC30A	Diff. RPE1 GFP-Centrin	Random
ENSG00000165533	ENST00000345383	TTC8	Diff. RPE1 GFP-Centrin	Random
ENSG00000135912	ENST00000392102	TTLL4	Diff. RPE1 GFP-Centrin	Random
ENSG00000119685	ENST00000298832	TTLL5	Diff. RPE1 GFP-Centrin	Random
ENSG00000123416	ENST00000336023	TUBA1B	Diff. RPE1 GFP-Centrin	Random
ENSG00000127824	ENST00000248437	TUBA4A	Diff. RPE1 GFP-Centrin	Random
ENSG00000137267	ENST00000333628	TUBB2A	Diff. RPE1 GFP-Centrin	Random
ENSG00000258947	ENST00000315491	TUBB3	Diff. RPE1 GFP-Centrin	Random
ENSG00000188229	ENST00000340384	TUBB4B	Diff. RPE1 GFP-Centrin	Random
ENSG00000176014	ENST00000317702	TUBB6	Diff. RPE1 GFP-Centrin	Random
ENSG00000074935	ENST00000368662	TUBE1	Diff. RPE1 GFP-Centrin	Random
ENSG00000137822	ENST00000564079	TUBGCP4	Diff. RPE1 GFP-Centrin	Random
ENSG00000112041	ENST00000322263	TULP1	Diff. RPE1 GFP-Centrin	Random
ENSG00000078246	ENST00000448120	TULP3	Diff. RPE1 GFP-Centrin	Random
ENSG00000130338	ENST00000367097	TULP4	Diff. RPE1 GFP-Centrin	Random
ENSG00000115514	ENST00000264255	TXNDC9	Diff. RPE1 GFP-Centrin	Random
ENSG00000127481	ENST00000375254	UBR4	Diff. RPE1 GFP-Centrin	Random
ENSG00000109103	ENST00000335765	UNC119	Diff. RPE1 GFP-Centrin	Random
ENSG00000175970	ENST00000344651	UNC119B	Diff. RPE1 GFP-Centrin	Random
ENSG00000135763	ENST00000258243	URB2	Diff. RPE1 GFP-Centrin	Random
ENSG00000124486	ENST00000324545	USP9X	Diff. RPE1 GFP-Centrin	Random
ENSG00000198382	ENST00000356136	UVRAG	Diff. RPE1 GFP-Centrin	Random
ENSG00000126756	ENST00000333119	UXT	Diff. RPE1 GFP-Centrin	Random

ENSG00000139722	ENST00000267202	VPS37B	Diff. RPE1 GFP-Centrin	Random
ENSG00000132612	ENST00000254950	VPS4A	Diff. RPE1 GFP-Centrin	Random
ENSG00000119541	ENST00000238497	VPS4B	Diff. RPE1 GFP-Centrin	Random
ENSG00000143951	ENST00000409354	WDPCP	Diff. RPE1 GFP-Centrin	Random
ENSG00000119333	ENST00000372715	WDR34	Diff. RPE1 GFP-Centrin	Random
ENSG00000118965	ENST00000281405	WDR35	Diff. RPE1 GFP-Centrin	Random
ENSG00000085433	ENST00000369962	WDR47	Diff. RPE1 GFP-Centrin	Random
ENSG00000126870	ENST00000407559	WDR60	Diff. RPE1 GFP-Centrin	Random
ENSG00000075702	ENST00000401500	WDR62	Diff. RPE1 GFP-Centrin	Random
ENSG00000095397	ENST00000265134	WHRN	Diff. RPE1 GFP-Centrin	Random
ENSG00000011451	ENST00000599686	WIZ	Diff. RPE1 GFP-Centrin	Random
ENSG00000116213	ENST00000270708	WRAP73	Diff. RPE1 GFP-Centrin	Random
ENSG00000165392	ENST00000298139	WRN	Diff. RPE1 GFP-Centrin	Random
ENSG00000196584	ENST00000359321	XRCC2	Diff. RPE1 GFP-Centrin	Random
ENSG00000164924	ENST00000395958	YWHAZ	Diff. RPE1 GFP-Centrin	Random
ENSG00000214717	ENST00000381223	ZBED1	Diff. RPE1 GFP-Centrin	Random
ENSG00000166140	ENST00000561768	ZFYVE19	Diff. RPE1 GFP-Centrin	Random
ENSG00000072121	ENST00000347230	ZFYVE26	Diff. RPE1 GFP-Centrin	Random
ENSG00000164631	ENST00000405858	ZNF12	Diff. RPE1 GFP-Centrin	Random
ENSG00000010244	ENST00000394673	ZNF207	Diff. RPE1 GFP-Centrin	Random
ENSG00000181315	ENST00000415922	ZNF322	Diff. RPE1 GFP-Centrin	Random
ENSG00000109445	ENST00000262990	ZNF330	Diff. RPE1 GFP-Centrin	Random
ENSG00000138311	ENST00000395254	ZNF365	Diff. RPE1 GFP-Centrin	Random
