# Supplementary material for: Exon junction complex dependent mRNA localization is linked to centrosome organization during ciliogenesis
Source: Nat Commun. 2021 Mar 1;12:1351. doi: 10.1038/s41467-021-21590-w (PMC7921557; doi:10.1038/s41467-021-21590-w)
Supplement: Supplementary file 3 — Supplementary Data 2 [file 41467_2021_21590_MOESM3_ESM.pdf]

Supplementary DATA 2

Oligonucleotide sequence (5' end on the left, 3' end on the right ; concatenation of first barcode (25 nucleotides), FLAP Y sequence : TTACACTCGGACCTCGTCGACATGCATT, target-specific hybridization sequence, FLAP X sequence : CCTCCTAAGTTTCGAGCTGGACTCAGTG, second barcode (25 nucleotides))

BICD2

CGAGGGGTACGATCTCCCTGTTCCATTACACTCGGACCTCGTCGACATGCATTACAATGACTGTTTTCTTTCCCTCAATGAAGATGCCTCCTAAGTTTCGAGCTGGACTCAGTGGGGGTTGGTTTACTCCTGGTTACC
CGAGGGGTACGATCTCCCTGTTCCATTACACTCGGACCTCGTCGACATGCATTGTGTAAAGGTTCCTTGGCAGGAGAACATGCCCTCCTAAGTTTCGAGCTGGACTCAGTGGGGGTTGGTTTACTCCTGGTTACC
CGAGGGGTACGATCTCCCTGTTCCATTACACTCGGACCTCGTCGACATGCATTGTCCTCCTGGCAGCTACTCTACAGGCTCCTCCTAAGTTTCGAGCTGGACTCAGTGGGGGTTGGTTTACTCCTGGTTACC
CGAGGGGTACGATCTCCCTGTTCCATTACACTCGGACCTCGTCGACATGCATTCGTTGATGTCCACCTCGTAGTAGTCCCCCCTCCTAAGTTTCGAGCTGGACTCAGTGGGGGTTGGTTTACTCCTGGTTACC
CGAGGGGTACGATCTCCCTGTTCCATTACACTCGGACCTCGTCGACATGCATTGAGCAAAGGTCAAGTTTTCTTCTTGTTACATTGAACCCTCCTAAGTTTCGAGCTGGACTCAGTGGGGGTTGGTTTACTCCTGGTTACC
CGAGGGGTACGATCTCCCTGTTCCATTACACTCGGACCTCGTCGACATGCATTTAGACCACGGCACCTGAGACCGTCTCTCCTCCTAAGTTTCGAGCTGGACTCAGTGGGGGTTGGTTTACTCCTGGTTACC
CGAGGGGTACGATCTCCCTGTTCCATTACACTCGGACCTCGTCGACATGCATTGAACTTGAGGCCATCCAGCGAGACATGCCCTCCTAAGTTTCGAGCTGGACTCAGTGGGGGTTGGTTTACTCCTGGTTACC
CGAGGGGTACGATCTCCCTGTTCCATTACACTCGGACCTCGTCGACATGCATTTGTAACAAGGAGCCCCCGTGGTGGGCCTCCTAAGTTTCGAGCTGGACTCAGTGGGGGTTGGTTTACTCCTGGTTACC
CGAGGGGTACGATCTCCCTGTTCCATTACACTCGGACCTCGTCGACATGCATTCAAACATAGCACGCAGCGAGGAGAAGGTGCCTCCTAAGTTTCGAGCTGGACTCAGTGGGGGTTGGTTTACTCCTGGTTACC
CGAGGGGTACGATCTCCCTGTTCCATTACACTCGGACCTCGTCGACATGCATTTTTTTCCCGCTCCATCTGCATCAGCTGCTCCTCCTAAGTTTCGAGCTGGACTCAGTGGGGGTTGGTTTACTCCTGGTTACC
CGAGGGGTACGATCTCCCTGTTCCATTACACTCGGACCTCGTCGACATGCATTTTGATCTGGTCACGGATGATAGCGATCAGGCCTCCTAAGTTTCGAGCTGGACTCAGTGGGGGTTGGTTTACTCCTGGTTACC
CGAGGGGTACGATCTCCCTGTTCCATTACACTCGGACCTCGTCGACATGCATTCCATACAGAGGCCTTTCCACGCAGCAGCCTCCTAAGTTTCGAGCTGGACTCAGTGGGGGTTGGTTTACTCCTGGTTACC
CGAGGGGTACGATCTCCCTGTTCCATTACACTCGGACCTCGTCGACATGCATTCACTGAGTAGGTCGGAGACGAGGCTGGCCTCCTAAGTTTCGAGCTGGACTCAGTGGGGGTTGGTTTACTCCTGGTTACC
CGAGGGGTACGATCTCCCTGTTCCATTACACTCGGACCTCGTCGACATGCATTGTCACTCAGGGGTGATGGCAGTGAGGCCTCCTAAGTTTCGAGCTGGACTCAGTGGGGGTTGGTTTACTCCTGGTTACC
CGAGGGGTACGATCTCCCTGTTCCATTACACTCGGACCTCGTCGACATGCATTTGTAGATGTTCATGGGCTCCCGGCGTGCCTCCTAAGTTTCGAGCTGGACTCAGTGGGGGTTGGTTTACTCCTGGTTACC
CGAGGGGTACGATCTCCCTGTTCCATTACACTCGGACCTCGTCGACATGCATTTCCTGATACCCTCAGCTCTTCACAAACGTGCCTCCTAAGTTTCGAGCTGGACTCAGTGGGGGTTGGTTTACTCCTGGTTACC
CGAGGGGTACGATCTCCCTGTTCCATTACACTCGGACCTCGTCGACATGCATTATCCTCCAGCTGGCTGTTGAGGTACTCGCCTCCTAAGTTTCGAGCTGGACTCAGTGGGGGTTGGTTTACTCCTGGTTACC
CGAGGGGTACGATCTCCCTGTTCCATTACACTCGGACCTCGTCGACATGCATTCTCAGATTCTCTGTGAGGCGGGTCACCCCTCCTAAGTTTCGAGCTGGACTCAGTGGGGGTTGGTTTACTCCTGGTTACC
CGAGGGGTACGATCTCCCTGTTCCATTACACTCGGACCTCGTCGACATGCATTTCGCAGCATGCTCAACATTAAAGCTTTTTTCCTCCTCCTAAGTTTCGAGCTGGACTCAGTGGGGGTTGGTTTACTCCTGGTTACC
CGAGGGGTACGATCTCCCTGTTCCATTACACTCGGACCTCGTCGACATGCATTTAGTCCAGCATGACACGGTTGGGTGTCTCCTCCTAAGTTTCGAGCTGGACTCAGTGGGGGTTGGTTTACTCCTGGTTACC
CGAGGGGTACGATCTCCCTGTTCCATTACACTCGGACCTCGTCGACATGCATTTTGAGGCCCTCAAACTCCACCTGGTTCTCCTCCTAAGTTTCGAGCTGGACTCAGTGGGGGTTGGTTTACTCCTGGTTACC
CGAGGGGTACGATCTCCCTGTTCCATTACACTCGGACCTCGTCGACATGCATTATTGTTGCACATGCACACGTGGTGGTAGAGCCTCCTAAGTTTCGAGCTGGACTCAGTGGGGGTTGGTTTACTCCTGGTTACC
CGAGGGGTACGATCTCCCTGTTCCATTACACTCGGACCTCGTCGACATGCATTATGGTACTTGCAGGCCAAGATCTCAGGCCCTCCTAAGTTTCGAGCTGGACTCAGTGGGGGTTGGTTTACTCCTGGTTACC
CGAGGGGTACGATCTCCCTGTTCCATTACACTCGGACCTCGTCGACATGCATTTCTCCTCCTCCAGACGCTTGATCTCATGCCTCCTAAGTTTCGAGCTGGACTCAGTGGGGGTTGGTTTACTCCTGGTTACC
CGAGGGGTACGATCTCCCTGTTCCATTACACTCGGACCTCGTCGACATGCATTGTTTTTCTTCCTCCAAGAGTACCAATTTGACCACCTCCTAAGTTTCGAGCTGGACTCAGTGGGGGTTGGTTTACTCCTGGTTACC
CGAGGGGTACGATCTCCCTGTTCCATTACACTCGGACCTCGTCGACATGCATTTATGGAGCACACAGCTCTGCCTCGTGCCCTCCTAAGTTTCGAGCTGGACTCAGTGGGGGTTGGTTTACTCCTGGTTACC
CGAGGGGTACGATCTCCCTGTTCCATTACACTCGGACCTCGTCGACATGCATTTGCTTCACAACGTCCTAAAACCCAGAAAATTCTCCTCCTAAGTTTCGAGCTGGACTCAGTGGGGGTTGGTTTACTCCTGGTTACC
CGAGGGGTACGATCTCCCTGTTCCATTACACTCGGACCTCGTCGACATGCATTCTTCAGCTTCTGGATCTCAGAGATGTTGAGCCCTCCTAAGTTTCGAGCTGGACTCAGTGGGGGTTGGTTTACTCCTGGTTACC
CGAGGGGTACGATCTCCCTGTTCCATTACACTCGGACCTCGTCGACATGCATTCAAAGCCATTGACCAGGGCCTCGGCACCTCCTAAGTTTCGAGCTGGACTCAGTGGGGGTTGGTTTACTCCTGGTTACC
CGAGGGGTACGATCTCCCTGTTCCATTACACTCGGACCTCGTCGACATGCATTCTGGCCTTCTCTAGCAGGGAGACCTTCCCTCCTAAGTTTCGAGCTGGACTCAGTGGGGGTTGGTTTACTCCTGGTTACC
CGAGGGGTACGATCTCCCTGTTCCATTACACTCGGACCTCGTCGACATGCATTTCCTCATGGCTGTCACGGTCCTTCTCGCCTCCTAAGTTTCGAGCTGGACTCAGTGGGGGTTGGTTTACTCCTGGTTACC
CGAGGGGTACGATCTCCCTGTTCCATTACACTCGGACCTCGTCGACATGCATTTGTCCAGGGCTGTCTGCCGCTCCTTCCTCCTAAGTTTCGAGCTGGACTCAGTGGGGGTTGGTTTACTCCTGGTTACC
CGAGGGGTACGATCTCCCTGTTCCATTACACTCGGACCTCGTCGACATGCATTTTGGCCAGCTCCTCACTGAAGGTCACCCCTCCTAAGTTTCGAGCTGGACTCAGTGGGGGTTGGTTTACTCCTGGTTACC
CGAGGGGTACGATCTCCCTGTTCCATTACACTCGGACCTCGTCGACATGCATTTCCTATTCCTTTTCCATGCTCTGCCTCATTTTCCTCCTAAGTTTCGAGCTGGACTCAGTGGGGGTTGGTTTACTCCTGGTTACC
CGAGGGGTACGATCTCCCTGTTCCATTACACTCGGACCTCGTCGACATGCATTTCTCCTGCTGTTCTGACAGGGAGCCCCCTCCTAAGTTTCGAGCTGGACTCAGTGGGGGTTGGTTTACTCCTGGTTACC
CGAGGGGTACGATCTCCCTGTTCCATTACACTCGGACCTCGTCGACATGCATTCACCTCGAGCTCCTCGAACTGCAGCTTCCTCCTAAGTTTCGAGCTGGACTCAGTGGGGGTTGGTTTACTCCTGGTTACC
CGAGGGGTACGATCTCCCTGTTCCATTACACTCGGACCTCGTCGACATGCATTATGTAGACCTGGTTTGCTAAGGAGTTTTAATGAGCCTCCTAAGTTTCGAGCTGGACTCAGTGGGGGTTGGTTTACTCCTGGTTACC
CGAGGGGTACGATCTCCCTGTTCCATTACACTCGGACCTCGTCGACATGCATTAAAGAATACTATGGCTAACCCTCATCCCCTACCCTCCTAAGTTTCGAGCTGGACTCAGTGGGGGTTGGTTTACTCCTGGTTACC
CGAGGGGTACGATCTCCCTGTTCCATTACACTCGGACCTCGTCGACATGCATTGACACCAGACAAACACGGTGGGAGCTGCCTCCTAAGTTTCGAGCTGGACTCAGTGGGGGTTGGTTTACTCCTGGTTACC
CGAGGGGTACGATCTCCCTGTTCCATTACACTCGGACCTCGTCGACATGCATTTACTCCTTGATGTCATCCCGCAGGCGGCCTCCTAAGTTTCGAGCTGGACTCAGTGGGGGTTGGTTTACTCCTGGTTACC
CGAGGGGTACGATCTCCCTGTTCCATTACACTCGGACCTCGTCGACATGCATTTCCATAAGCGCTTCCTTGTCCTTGTCCACCCTCCTAAGTTTCGAGCTGGACTCAGTGGGGGTTGGTTTACTCCTGGTTACC
CGAGGGGTACGATCTCCCTGTTCCATTACACTCGGACCTCGTCGACATGCATTGCTGTGTAATGTACTCGTCACACCTGGTGCCTCCTAAGTTTCGAGCTGGACTCAGTGGGGGTTGGTTTACTCCTGGTTACC
CGAGGGGTACGATCTCCCTGTTCCATTACACTCGGACCTCGTCGACATGCATTCGTTGTTGGGCTCGGCAGCATCGTCACCCTCCTAAGTTTCGAGCTGGACTCAGTGGGGGTTGGTTTACTCCTGGTTACC
CGAGGGGTACGATCTCCCTGTTCCATTACACTCGGACCTCGTCGACATGCATTTCACCTTCTTTAGCTCCTTCTCCAGCCGCCTCCTAAGTTTCGAGCTGGACTCAGTGGGGGTTGGTTTACTCCTGGTTACC
CGAGGGGTACGATCTCCCTGTTCCATTACACTCGGACCTCGTCGACATGCATTGCTCATCCTGGGCCACACTCAGGCTCCTCCTAAGTTTCGAGCTGGACTCAGTGGGGGTTGGTTTACTCCTGGTTACC
CGAGGGGTACGATCTCCCTGTTCCATTACACTCGGACCTCGTCGACATGCATTAAAATAGGAGTCCCTACAACTAGGAATAAGAAATGCCCTCCTAAGTTTCGAGCTGGACTCAGTGGGGGTTGGTTTACTCCTGGTTACC
CGAGGGGTACGATCTCCCTGTTCCATTACACTCGGACCTCGTCGACATGCATTTGCTTGTCCAAAGGCCTCCTTGAGCTGCCTCCTAAGTTTCGAGCTGGACTCAGTGGGGGTTGGTTTACTCCTGGTTACC
CGAGGGGTACGATCTCCCTGTTCCATTACACTCGGACCTCGTCGACATGCATTCGTTCATACAGCTTGCTCAGCTTGTCCCACCTCCTAAGTTTCGAGCTGGACTCAGTGGGGGTTGGTTTACTCCTGGTTACC
CGAGGGGTACGATCTCCCTGTTCCATTACACTCGGACCTCGTCGACATGCATTGGTTTTGTGTTTGCTTTCATTTTCTAAGCCCCTCCTCCTAAGTTTCGAGCTGGACTCAGTGGGGGTTGGTTTACTCCTGGTTACC
CGAGGGGTACGATCTCCCTGTTCCATTACACTCGGACCTCGTCGACATGCATTTGAGCAGCGACTTCAGCTTGAGGATCTCCCTCCTAAGTTTCGAGCTGGACTCAGTGGGGGTTGGTTTACTCCTGGTTACC
CGAGGGGTACGATCTCCCTGTTCCATTACACTCGGACCTCGTCGACATGCATTAAAGCACTCATCTCCGAGTCTAAAGCTACACGCCTCCTAAGTTTCGAGCTGGACTCAGTGGGGGTTGGTTTACTCCTGGTTACC
CGAGGGGTACGATCTCCCTGTTCCATTACACTCGGACCTCGTCGACATGCATTGCGTGTGCTCCAGCTGCTTCTGTGTGTCCTCCTAAGTTTCGAGCTGGACTCAGTGGGGGTTGGTTTACTCCTGGTTACC
CGAGGGGTACGATCTCCCTGTTCCATTACACTCGGACCTCGTCGACATGCATTATCTGAATACCCTGGATTTCTTAATGAACATGGCCCTCCTAAGTTTCGAGCTGGACTCAGTGGGGGTTGGTTTACTCCTGGTTACC
CGAGGGGTACGATCTCCCTGTTCCATTACACTCGGACCTCGTCGACATGCATTATGTGACTCTTTACCCAGGCCCACTGTTCCCTCCTAAGTTTCGAGCTGGACTCAGTGGGGGTTGGTTTACTCCTGGTTACC
CGAGGGGTACGATCTCCCTGTTCCATTACACTCGGACCTCGTCGACATGCATTTATGCGCACTGGCTTTGTAGGCATTCACATCCTCCTAAGTTTCGAGCTGGACTCAGTGGGGGTTGGTTTACTCCTGGTTACC
CGAGGGGTACGATCTCCCTGTTCCATTACACTCGGACCTCGTCGACATGCATTTCCCACTAACCTCACTCAGCAAACAAAACAGCCTCCTAAGTTTCGAGCTGGACTCAGTGGGGGTTGGTTTACTCCTGGTTACC
CGAGGGGTACGATCTCCCTGTTCCATTACACTCGGACCTCGTCGACATGCATTGGTGGCTGGTGTAGAAGGAGTCATTGATGCCTCCTAAGTTTCGAGCTGGACTCAGTGGGGGTTGGTTTACTCCTGGTTACC
CGAGGGGTACGATCTCCCTGTTCCATTACACTCGGACCTCGTCGACATGCATTCATTGCGCAGCTTCATCATGGTCTCGGTAACCTCCTAAGTTTCGAGCTGGACTCAGTGGGGGTTGGTTTACTCCTGGTTACC
CGAGGGGTACGATCTCCCTGTTCCATTACACTCGGACCTCGTCGACATGCATTGCAAGAGAAGATATAATGTCTCGCTTTCACATTTGCCTCCTAAGTTTCGAGCTGGACTCAGTGGGGGTTGGTTTACTCCTGGTTACC
CGAGGGGTACGATCTCCCTGTTCCATTACACTCGGACCTCGTCGACATGCATTCATGCCCACTAGCTCTTCCCTGCTCGCCCTCCTAAGTTTCGAGCTGGACTCAGTGGGGGTTGGTTTACTCCTGGTTACC
CGAGGGGTACGATCTCCCTGTTCCATTACACTCGGACCTCGTCGACATGCATTTCCATCTCGCTGCGGATAGCCTCATAGTCCTCCTAAGTTTCGAGCTGGACTCAGTGGGGGTTGGTTTACTCCTGGTTACC
CGAGGGGTACGATCTCCCTGTTCCATTACACTCGGACCTCGTCGACATGCATTCCTGCAGCAGACGAGCTTCCCGGAATTTCCTCCTAAGTTTCGAGCTGGACTCAGTGGGGGTTGGTTTACTCCTGGTTACC
CGAGGGGTACGATCTCCCTGTTCCATTACACTCGGACCTCGTCGACATGCATTGAGGACATTGCGCAACTGCTTCAGCTCCCCTCCTAAGTTTCGAGCTGGACTCAGTGGGGGTTGGTTTACTCCTGGTTACC
CGAGGGGTACGATCTCCCTGTTCCATTACACTCGGACCTCGTCGACATGCATTGCAGTGGCATATCCAAAAAGTTTCTAAGTGGGCCTCCTAAGTTTCGAGCTGGACTCAGTGGGGGTTGGTTTACTCCTGGTTACC
CGAGGGGTACGATCTCCCTGTTCCATTACACTCGGACCTCGTCGACATGCATTATGATCTAAAGGCTGCTGAACCACAGCGTGCCTCCTAAGTTTCGAGCTGGACTCAGTGGGGGTTGGTTTACTCCTGGTTACC
CGAGGGGTACGATCTCCCTGTTCCATTACACTCGGACCTCGTCGACATGCATTTTTTAGGTTGCCCTTCCTTCCTCCTTGTGGCCTCCTAAGTTTCGAGCTGGACTCAGTGGGGGTTGGTTTACTCCTGGTTACC
CGAGGGGTACGATCTCCCTGTTCCATTACACTCGGACCTCGTCGACATGCATTTCTGAGCACAGACACTTGCTTCTGCAGGCCTCCTAAGTTTCGAGCTGGACTCAGTGGGGGTTGGTTTACTCCTGGTTACC
CGAGGGGTACGATCTCCCTGTTCCATTACACTCGGACCTCGTCGACATGCATTTCACCTGCTGTCCACGCTGGCCTTCACCTCCTAAGTTTCGAGCTGGACTCAGTGGGGGTTGGTTTACTCCTGGTTACC
CGAGGGGTACGATCTCCCTGTTCCATTACACTCGGACCTCGTCGACATGCATTGTTCAGCGTCTTCTTCTCGTCCTCAGCAGCCTCCTAAGTTTCGAGCTGGACTCAGTGGGGGTTGGTTTACTCCTGGTTACC
CGAGGGGTACGATCTCCCTGTTCCATTACACTCGGACCTCGTCGACATGCATTGCTCTGAGATCTCCTTGAGGCGGATGGCCTCCTAAGTTTCGAGCTGGACTCAGTGGGGGTTGGTTTACTCCTGGTTACC
CGAGGGGTACGATCTCCCTGTTCCATTACACTCGGACCTCGTCGACATGCATTACGACTGTAGAATTTTCTAAGTGTACCACAATTTGGCCTCCTAAGTTTCGAGCTGGACTCAGTGGGGGTTGGTTTACTCCTGGTTACC
CGAGGGGTACGATCTCCCTGTTCCATTACACTCGGACCTCGTCGACATGCATTCACGCTGGATCTCCACATTCTGGTTGATCTCCTCCTAAGTTTCGAGCTGGACTCAGTGGGGGTTGGTTTACTCCTGGTTACC
CGAGGGGTACGATCTCCCTGTTCCATTACACTCGGACCTCGTCGACATGCATTTTTGAGAATGATCTGTGCTGGGACACCTCCCTCCTAAGTTTCGAGCTGGACTCAGTGGGGGTTGGTTTACTCCTGGTTACC
CGAGGGGTACGATCTCCCTGTTCCATTACACTCGGACCTCGTCGACATGCATTCTTAACTGATGAAGGCACTACTGGCAATGATTACCTCCTAAGTTTCGAGCTGGACTCAGTGGGGGTTGGTTTACTCCTGGTTACC
CGAGGGGTACGATCTCCCTGTTCCATTACACTCGGACCTCGTCGACATGCATTCTTGGGCGTGGAGGTCTTGTTGTCCAGTCCTCCTAAGTTTCGAGCTGGACTCAGTGGGGGTTGGTTTACTCCTGGTTACC
CGAGGGGTACGATCTCCCTGTTCCATTACACTCGGACCTCGTCGACATGCATTGTACTGCTCCTTGGAGGCCGACTCCTCCTCCTAAGTTTCGAGCTGGACTCAGTGGGGGTTGGTTTACTCCTGGTTACC
CGAGGGGTACGATCTCCCTGTTCCATTACACTCGGACCTCGTCGACATGCATTCTGCTCATGGTCCAGCTCGAGCAGCTCCTCCTAAGTTTCGAGCTGGACTCAGTGGGGGTTGGTTTACTCCTGGTTACC
CGAGGGGTACGATCTCCCTGTTCCATTACACTCGGACCTCGTCGACATGCATTTCATGTAGTGTGACAGCTCCTTGCGCAGCCTCCTAAGTTTCGAGCTGGACTCAGTGGGGGTTGGTTTACTCCTGGTTACC
CGAGGGGTACGATCTCCCTGTTCCATTACACTCGGACCTCGTCGACATGCATTGTCAGCAGCCACCTTCTTGTGGTTTGTGTCCTCCTAAGTTTCGAGCTGGACTCAGTGGGGGTTGGTTTACTCCTGGTTACC
CGAGGGGTACGATCTCCCTGTTCCATTACACTCGGACCTCGTCGACATGCATTTGCTTGTTGGCCTTGAGCACAGTGCGCCCTCCTAAGTTTCGAGCTGGACTCAGTGGGGGTTGGTTTACTCCTGGTTACC
CGAGGGGTACGATCTCCCTGTTCCATTACACTCGGACCTCGTCGACATGCATTAAGGTCCATTTGGTTCCCAAAGCACACTCACCTCCTAAGTTTCGAGCTGGACTCAGTGGGGGTTGGTTTACTCCTGGTTACC
CGAGGGGTACGATCTCCCTGTTCCATTACACTCGGACCTCGTCGACATGCATTGGCGCTCATTCTCCGACTGCGTGTTGGCCTCCTAAGTTTCGAGCTGGACTCAGTGGGGGTTGGTTTACTCCTGGTTACC
CGAGGGGTACGATCTCCCTGTTCCATTACACTCGGACCTCGTCGACATGCATTATACACTTAGCTGAGCTCCTCGCTGGGTCCTCCTAAGTTTCGAGCTGGACTCAGTGGGGGTTGGTTTACTCCTGGTTACC
CGAGGGGTACGATCTCCCTGTTCCATTACACTCGGACCTCGTCGACATGCATTCTGTTCTTCTGTTCGCGCTCCGTCTTCAGCCTCCTAAGTTTCGAGCTGGACTCAGTGGGGGTTGGTTTACTCCTGGTTACC
CGAGGGGTACGATCTCCCTGTTCCATTACACTCGGACCTCGTCGACATGCATTTGATGTTCTCCTCCTCCAGTTCCGAGTAGCCTCCTAAGTTTCGAGCTGGACTCAGTGGGGGTTGGTTTACTCCTGGTTACC
CGAGGGGTACGATCTCCCTGTTCCATTACACTCGGACCTCGTCGACATGCATTCATGGCCTTCTCATTCTCATACTTGCTCTTCACCTCCTAAGTTTCGAGCTGGACTCAGTGGGGGTTGGTTTACTCCTGGTTACC
CGAGGGGTACGATCTCCCTGTTCCATTACACTCGGACCTCGTCGACATGCATTTCTGCAGCTCTAGCACCTTCCGCACGTCCTCCTAAGTTTCGAGCTGGACTCAGTGGGGGTTGGTTTACTCCTGGTTACC

NIN

ATCCTGGGTTCTGCCTACAGTCAGATTACACTCGGACCTCGTCGACATGCATTACCTCAGGTGCTGCATCTTCACACACTATCCTCCTAAGTTTCGAGCTGGACTCAGTGGGCGTTGAGTTGGTCCCTTCGTTAG
ATCCTGGGTTCTGCCTACAGTCAGATTACACTCGGACCTCGTCGACATGCATTTCATTTTCCAGACGACTGTTTTCCTTTAAAGAGAGCCTCCTAAGTTTCGAGCTGGACTCAGTGGGCGTTGAGTTGGTCCCTTCGTTAG

ATCCTGGGTTCTGCCTACAGTCAGATTACACTCGGACCTCGTCGACATGCATTGCGCTACTCTGAAGGCCTTCCCTCTCCCTCCTAAGTTTCGAGCTGGACTCAGTGGGCGTTGAGTTGGTCCCTTCGTTAG
ATCCTGGGTTCTGCCTACAGTCAGATTACACTCGGACCTCGTCGACATGCATTCGCTCAAAGCTTGCTTGAGCCTGTGTCAGCCTCCTAAGTTTCGAGCTGGACTCAGTGGGCGTTGAGTTGGTCCCTTCGTTAG
ATCCTGGGTTCTGCCTACAGTCAGATTACACTCGGACCTCGTCGACATGCATTCTAGCCTTGAGCTCCATCTCATGTTGCAGCCTCCTAAGTTTCGAGCTGGACTCAGTGGGCGTTGAGTTGGTCCCTTCGTTAG
ATCCTGGGTTCTGCCTACAGTCAGATTACACTCGGACCTCGTCGACATGCATTCATATTCTGCCAACTTCTCTGCATTTTCTAGAAGCCTCCTAAGTTTCGAGCTGGACTCAGTGGGCGTTGAGTTGGTCCCTTCGTTAG
ATCCTGGGTTCTGCCTACAGTCAGATTACACTCGGACCTCGTCGACATGCATTTCTCTTCTTGCAGGAAGAACTCAGCACTGCCCTCCTAAGTTTCGAGCTGGACTCAGTGGGCGTTGAGTTGGTCCCTTCGTTAG
ATCCTGGGTTCTGCCTACAGTCAGATTACACTCGGACCTCGTCGACATGCATTTCTGTTAGACGTTGATTAAGTTCTTGCAGTTTTTCCCCTCCTAAGTTTCGAGCTGGACTCAGTGGGCGTTGAGTTGGTCCCTTCGTTAG
ATCCTGGGTTCTGCCTACAGTCAGATTACACTCGGACCTCGTCGACATGCATTTTCCAAGAGCTCTTTTCTTAACTCCTCTTTCTCACCTCCTAAGTTTCGAGCTGGACTCAGTGGGCGTTGAGTTGGTCCCTTCGTTAG
ATCCTGGGTTCTGCCTACAGTCAGATTACACTCGGACCTCGTCGACATGCATTGTCAAGCCTCTCACCTTCTCTTCTGTCCACCTCCTAAGTTTCGAGCTGGACTCAGTGGGCGTTGAGTTGGTCCCTTCGTTAG
ATCCTGGGTTCTGCCTACAGTCAGATTACACTCGGACCTCGTCGACATGCATTGTTTTTCCTTCACAAACCAATGTTCCCTTAAGTCCCTCCTAAGTTTCGAGCTGGACTCAGTGGGCGTTGAGTTGGTCCCTTCGTTAG
ATCCTGGGTTCTGCCTACAGTCAGATTACACTCGGACCTCGTCGACATGCATTATTAAAGACTTGAGCTTCTCGGCCTTGTCCACCTCCTAAGTTTCGAGCTGGACTCAGTGGGCGTTGAGTTGGTCCCTTCGTTAG
ATCCTGGGTTCTGCCTACAGTCAGATTACACTCGGACCTCGTCGACATGCATTAGCCTTAAAGCTGGCCAGAGCCGCCTCCTCCTAAGTTTCGAGCTGGACTCAGTGGGCGTTGAGTTGGTCCCTTCGTTAG
ATCCTGGGTTCTGCCTACAGTCAGATTACACTCGGACCTCGTCGACATGCATTTGCTCCTGGTGAAACTGCTCTAGTTCCTGCCTCCTAAGTTTCGAGCTGGACTCAGTGGGCGTTGAGTTGGTCCCTTCGTTAG
ATCCTGGGTTCTGCCTACAGTCAGATTACACTCGGACCTCGTCGACATGCATTTCCGTAACGCTTCCCACCTCTAACATATTTGCCTCCTAAGTTTCGAGCTGGACTCAGTGGGCGTTGAGTTGGTCCCTTCGTTAG
ATCCTGGGTTCTGCCTACAGTCAGATTACACTCGGACCTCGTCGACATGCATTGTGCACGATATTCTTCCAGCTCAGACTGGACCTCCTAAGTTTCGAGCTGGACTCAGTGGGCGTTGAGTTGGTCCCTTCGTTAG
ATCCTGGGTTCTGCCTACAGTCAGATTACACTCGGACCTCGTCGACATGCATTAGCTGGCGTTGAAGCTGCAGAAACTGCTCCTCCTAAGTTTCGAGCTGGACTCAGTGGGCGTTGAGTTGGTCCCTTCGTTAG
ATCCTGGGTTCTGCCTACAGTCAGATTACACTCGGACCTCGTCGACATGCATTAGGGCCAGTGTTAATTCTGTCAAATTGATGTTTCCCTCCTAAGTTTCGAGCTGGACTCAGTGGGCGTTGAGTTGGTCCCTTCGTTAG
ATCCTGGGTTCTGCCTACAGTCAGATTACACTCGGACCTCGTCGACATGCATTGAGTGTGTTTCTCCACCAGGCTTGTCAGCCTCCTAAGTTTCGAGCTGGACTCAGTGGGCGTTGAGTTGGTCCCTTCGTTAG
ATCCTGGGTTCTGCCTACAGTCAGATTACACTCGGACCTCGTCGACATGCATTTCCTGGCTCTTTTTCCTTCTGGCATAGCATCCTCCTAAGTTTCGAGCTGGACTCAGTGGGCGTTGAGTTGGTCCCTTCGTTAG
ATCCTGGGTTCTGCCTACAGTCAGATTACACTCGGACCTCGTCGACATGCATTTTAGCCTTGCCTTGACTGGAAATACCTTTAAGACCTCCTAAGTTTCGAGCTGGACTCAGTGGGCGTTGAGTTGGTCCCTTCGTTAG
ATCCTGGGTTCTGCCTACAGTCAGATTACACTCGGACCTCGTCGACATGCATTATTGCTGATGAGGTTGTGGTACGTCGTCCCCTCCTAAGTTTCGAGCTGGACTCAGTGGGCGTTGAGTTGGTCCCTTCGTTAG
ATCCTGGGTTCTGCCTACAGTCAGATTACACTCGGACCTCGTCGACATGCATTAAAGACATCACTTCTTGTTTTAAAGCCTCCTTTTCCCCTCCTAAGTTTCGAGCTGGACTCAGTGGGCGTTGAGTTGGTCCCTTCGTTAG
ATCCTGGGTTCTGCCTACAGTCAGATTACACTCGGACCTCGTCGACATGCATTCTTCTAAATTCTGAACCTTTTCCTGAGAAGCCACCCTCCTAAGTTTCGAGCTGGACTCAGTGGGCGTTGAGTTGGTCCCTTCGTTAG
ATCCTGGGTTCTGCCTACAGTCAGATTACACTCGGACCTCGTCGACATGCATTCTCAGCTTCTCCTGCAGGTGAGTCTTTTCCCTCCTAAGTTTCGAGCTGGACTCAGTGGGCGTTGAGTTGGTCCCTTCGTTAG
ATCCTGGGTTCTGCCTACAGTCAGATTACACTCGGACCTCGTCGACATGCATTTCCACTCTGTGAAGCATTCAAGTCATCTGGCCTCCTAAGTTTCGAGCTGGACTCAGTGGGCGTTGAGTTGGTCCCTTCGTTAG
ATCCTGGGTTCTGCCTACAGTCAGATTACACTCGGACCTCGTCGACATGCATTTCATCTGTTCAATGACCAGCTCTGCCTCAACCTCCTAAGTTTCGAGCTGGACTCAGTGGGCGTTGAGTTGGTCCCTTCGTTAG
ATCCTGGGTTCTGCCTACAGTCAGATTACACTCGGACCTCGTCGACATGCATTGCTCATATTCAATGGATTGCATTCTTCAGAACCGCCTCCTAAGTTTCGAGCTGGACTCAGTGGGCGTTGAGTTGGTCCCTTCGTTAG
ATCCTGGGTTCTGCCTACAGTCAGATTACACTCGGACCTCGTCGACATGCATTAGAAGCCTTTTCACTAGTTTCAGTTTCTGTTCAACCCTCCTAAGTTTCGAGCTGGACTCAGTGGGCGTTGAGTTGGTCCCTTCGTTAG
ATCCTGGGTTCTGCCTACAGTCAGATTACACTCGGACCTCGTCGACATGCATTATCTTTGAGAAGGTGGTTTTCCTGTTGCACAATCCTCCTAAGTTTCGAGCTGGACTCAGTGGGCGTTGAGTTGGTCCCTTCGTTAG
ATCCTGGGTTCTGCCTACAGTCAGATTACACTCGGACCTCGTCGACATGCATTTGGGTCTGTATTTTAACTTCAGAGAGCTCCGCCTCCTAAGTTTCGAGCTGGACTCAGTGGGCGTTGAGTTGGTCCCTTCGTTAG
ATCCTGGGTTCTGCCTACAGTCAGATTACACTCGGACCTCGTCGACATGCATTCTCTTGTTCCCGTTCCTCCAATGCACTGTCCTCCTAAGTTTCGAGCTGGACTCAGTGGGCGTTGAGTTGGTCCCTTCGTTAG
ATCCTGGGTTCTGCCTACAGTCAGATTACACTCGGACCTCGTCGACATGCATTCTGTTAGCCTCAACTTCTTCTGACGGTGAGTCCTCCTAAGTTTCGAGCTGGACTCAGTGGGCGTTGAGTTGGTCCCTTCGTTAG
ATCCTGGGTTCTGCCTACAGTCAGATTACACTCGGACCTCGTCGACATGCATTCTTCAACGGAAGCCTGAGCACTCTGCCTCCTCCTAAGTTTCGAGCTGGACTCAGTGGGCGTTGAGTTGGTCCCTTCGTTAG
ATCCTGGGTTCTGCCTACAGTCAGATTACACTCGGACCTCGTCGACATGCATTTTCATCTACTTGGTCTTGTAGTACCCTGCACCCTCCTAAGTTTCGAGCTGGACTCAGTGGGCGTTGAGTTGGTCCCTTCGTTAG
ATCCTGGGTTCTGCCTACAGTCAGATTACACTCGGACCTCGTCGACATGCATTGCTGACAATAGCAACAATTTCTCCTTTCCTTCACCTCCTAAGTTTCGAGCTGGACTCAGTGGGCGTTGAGTTGGTCCCTTCGTTAG
ATCCTGGGTTCTGCCTACAGTCAGATTACACTCGGACCTCGTCGACATGCATTCTTCTACTCTCCCTTCACAAATCTAAGCTTAAGTCCTCCTAAGTTTCGAGCTGGACTCAGTGGGCGTTGAGTTGGTCCCTTCGTTAG
ATCCTGGGTTCTGCCTACAGTCAGATTACACTCGGACCTCGTCGACATGCATTGACACAGCTATTTAATTCCTCACTCAGAGCTTCCCTCCTAAGTTTCGAGCTGGACTCAGTGGGCGTTGAGTTGGTCCCTTCGTTAG
ATCCTGGGTTCTGCCTACAGTCAGATTACACTCGGACCTCGTCGACATGCATTTAGAGATTTTTGCTGGAAGTCAGAGAGATCGGCCTCCTAAGTTTCGAGCTGGACTCAGTGGGCGTTGAGTTGGTCCCTTCGTTAG
ATCCTGGGTTCTGCCTACAGTCAGATTACACTCGGACCTCGTCGACATGCATTTCCTGAGATCCATTTAATGTCCCTAATTTCAGGCCTCCTAAGTTTCGAGCTGGACTCAGTGGGCGTTGAGTTGGTCCCTTCGTTAG
ATCCTGGGTTCTGCCTACAGTCAGATTACACTCGGACCTCGTCGACATGCATTCCAGGATTTCTGTTCCTGCTTCATCGTCGCCCTCCTAAGTTTCGAGCTGGACTCAGTGGGCGTTGAGTTGGTCCCTTCGTTAG
ATCCTGGGTTCTGCCTACAGTCAGATTACACTCGGACCTCGTCGACATGCATTACATCTGTGCAGCTGTTTCATTTTCTCCAGTTCCTCCTAAGTTTCGAGCTGGACTCAGTGGGCGTTGAGTTGGTCCCTTCGTTAG
ATCCTGGGTTCTGCCTACAGTCAGATTACACTCGGACCTCGTCGACATGCATTCTGACCACGGTTTCGTCCAGCTGCTTTTCCCTCCTAAGTTTCGAGCTGGACTCAGTGGGCGTTGAGTTGGTCCCTTCGTTAG
ATCCTGGGTTCTGCCTACAGTCAGATTACACTCGGACCTCGTCGACATGCATTTAATGGCGCACTTTATCTTCGAGCTCCAGTCCCTCCTAAGTTTCGAGCTGGACTCAGTGGGCGTTGAGTTGGTCCCTTCGTTAG
ATCCTGGGTTCTGCCTACAGTCAGATTACACTCGGACCTCGTCGACATGCATTGAGGCAACATATGTCCCTGTGATGTTGTTCTCCTCCTAAGTTTCGAGCTGGACTCAGTGGGCGTTGAGTTGGTCCCTTCGTTAG
ATCCTGGGTTCTGCCTACAGTCAGATTACACTCGGACCTCGTCGACATGCATTCCTCGAGCATCTCTCCATCCACATTCTGTCCTCCTAAGTTTCGAGCTGGACTCAGTGGGCGTTGAGTTGGTCCCTTCGTTAG
ATCCTGGGTTCTGCCTACAGTCAGATTACACTCGGACCTCGTCGACATGCATTTCTTCCGGTTCAGGTGACCATCACGGGCCTCCTAAGTTTCGAGCTGGACTCAGTGGGCGTTGAGTTGGTCCCTTCGTTAG
ATCCTGGGTTCTGCCTACAGTCAGATTACACTCGGACCTCGTCGACATGCATTTCTTCTATCCAGTCTTGGGGAGGGGAAGCCTCCTAAGTTTCGAGCTGGACTCAGTGGGCGTTGAGTTGGTCCCTTCGTTAG
ATCCTGGGTTCTGCCTACAGTCAGATTACACTCGGACCTCGTCGACATGCATTTTCATCAGATGATCCAACTTGTCCCAGGACCCTCCTAAGTTTCGAGCTGGACTCAGTGGGCGTTGAGTTGGTCCCTTCGTTAG
ATCCTGGGTTCTGCCTACAGTCAGATTACACTCGGACCTCGTCGACATGCATTTTCTCTTGCCAAAGCAGCTGCTGTTCCTCCCTCCTAAGTTTCGAGCTGGACTCAGTGGGCGTTGAGTTGGTCCCTTCGTTAG
ATCCTGGGTTCTGCCTACAGTCAGATTACACTCGGACCTCGTCGACATGCATTGCCTTTTCTGCTGGTTATGGAGCCCTGACCTCCTAAGTTTCGAGCTGGACTCAGTGGGCGTTGAGTTGGTCCCTTCGTTAG
ATCCTGGGTTCTGCCTACAGTCAGATTACACTCGGACCTCGTCGACATGCATTCCAAGCTCAACATGTGGCAAAGGTCGGTGCCTCCTAAGTTTCGAGCTGGACTCAGTGGGCGTTGAGTTGGTCCCTTCGTTAG
ATCCTGGGTTCTGCCTACAGTCAGATTACACTCGGACCTCGTCGACATGCATTTCCTCAAGCTTCACTTGCAGTTGTTTTTCTCCCCTCCTAAGTTTCGAGCTGGACTCAGTGGGCGTTGAGTTGGTCCCTTCGTTAG
ATCCTGGGTTCTGCCTACAGTCAGATTACACTCGGACCTCGTCGACATGCATTTTTTTTCTAAGGTGTGCGTTTCGTTCTCATGCCCCTCCTAAGTTTCGAGCTGGACTCAGTGGGCGTTGAGTTGGTCCCTTCGTTAG
ATCCTGGGTTCTGCCTACAGTCAGATTACACTCGGACCTCGTCGACATGCATTTTGCTTCATGTTCTCCTGTGCCTTCTTGCACCTCCTAAGTTTCGAGCTGGACTCAGTGGGCGTTGAGTTGGTCCCTTCGTTAG
ATCCTGGGTTCTGCCTACAGTCAGATTACACTCGGACCTCGTCGACATGCATTGTGAATGCTGTTCTTGGTAACCAAAAGTTCATTTTCCCTCCTAAGTTTCGAGCTGGACTCAGTGGGCGTTGAGTTGGTCCCTTCGTTAG
ATCCTGGGTTCTGCCTACAGTCAGATTACACTCGGACCTCGTCGACATGCATTCTCCTGGCTGTTCTCAATGCCCTCTTCCCCTCCTAAGTTTCGAGCTGGACTCAGTGGGCGTTGAGTTGGTCCCTTCGTTAG
ATCCTGGGTTCTGCCTACAGTCAGATTACACTCGGACCTCGTCGACATGCATTGCCAGGTGTCCAGTATTCTCTCCACAGACCTCCTAAGTTTCGAGCTGGACTCAGTGGGCGTTGAGTTGGTCCCTTCGTTAG
ATCCTGGGTTCTGCCTACAGTCAGATTACACTCGGACCTCGTCGACATGCATTTATTTTTGTCCTTAGCCTAGGCCAATCCACAGCCTCCTAAGTTTCGAGCTGGACTCAGTGGGCGTTGAGTTGGTCCCTTCGTTAG
ATCCTGGGTTCTGCCTACAGTCAGATTACACTCGGACCTCGTCGACATGCATTTTTCTCTTGTGTTCCTGCCCATAAAGCAGTGCCTCCTAAGTTTCGAGCTGGACTCAGTGGGCGTTGAGTTGGTCCCTTCGTTAG
ATCCTGGGTTCTGCCTACAGTCAGATTACACTCGGACCTCGTCGACATGCATTCTCCTTGACCTCAGGTGGCATGCCAATGCCTCCTAAGTTTCGAGCTGGACTCAGTGGGCGTTGAGTTGGTCCCTTCGTTAG
ATCCTGGGTTCTGCCTACAGTCAGATTACACTCGGACCTCGTCGACATGCATTGTCTGCTTATCCCTCTTTTCTAAGAGTAGCTATTCCTCCTAAGTTTCGAGCTGGACTCAGTGGGCGTTGAGTTGGTCCCTTCGTTAG
ATCCTGGGTTCTGCCTACAGTCAGATTACACTCGGACCTCGTCGACATGCATTACTCCAAAGTGGTTTGTTCAGTGCTTTCCCTCCTCCTAAGTTTCGAGCTGGACTCAGTGGGCGTTGAGTTGGTCCCTTCGTTAG
ATCCTGGGTTCTGCCTACAGTCAGATTACACTCGGACCTCGTCGACATGCATTGCAAGGCGGTCCCGGATATAGTTCTCTTCCCTCCTAAGTTTCGAGCTGGACTCAGTGGGCGTTGAGTTGGTCCCTTCGTTAG
ATCCTGGGTTCTGCCTACAGTCAGATTACACTCGGACCTCGTCGACATGCATTGGATGAGTAGCTATCTCTGGGGCCCAGCCTCCTAAGTTTCGAGCTGGACTCAGTGGGCGTTGAGTTGGTCCCTTCGTTAG
ATCCTGGGTTCTGCCTACAGTCAGATTACACTCGGACCTCGTCGACATGCATTGCTGAGTCACTCGTAGGTCAGATTTCATCCCCTCCTAAGTTTCGAGCTGGACTCAGTGGGCGTTGAGTTGGTCCCTTCGTTAG
ATCCTGGGTTCTGCCTACAGTCAGATTACACTCGGACCTCGTCGACATGCATTGGACATTTGCAGGTTTACATTCTGCACGGTGCCTCCTAAGTTTCGAGCTGGACTCAGTGGGCGTTGAGTTGGTCCCTTCGTTAG
ATCCTGGGTTCTGCCTACAGTCAGATTACACTCGGACCTCGTCGACATGCATTAGCTGTGACTTTAAGCTCGCACTCTGATGTTCCTCCTAAGTTTCGAGCTGGACTCAGTGGGCGTTGAGTTGGTCCCTTCGTTAG
ATCCTGGGTTCTGCCTACAGTCAGATTACACTCGGACCTCGTCGACATGCATTGCAAGATCTCTTCCTATGCCTTGGAAGCCCCTCCTAAGTTTCGAGCTGGACTCAGTGGGCGTTGAGTTGGTCCCTTCGTTAG
ATCCTGGGTTCTGCCTACAGTCAGATTACACTCGGACCTCGTCGACATGCATTTGAGAGAGGTGGGTTTTACTATCCACATTTTCCCTCCTAAGTTTCGAGCTGGACTCAGTGGGCGTTGAGTTGGTCCCTTCGTTAG
ATCCTGGGTTCTGCCTACAGTCAGATTACACTCGGACCTCGTCGACATGCATTTTTCCCTTTTCTTGAAACGAAGATCCCATCTTCTCCTCCTAAGTTTCGAGCTGGACTCAGTGGGCGTTGAGTTGGTCCCTTCGTTAG
ATCCTGGGTTCTGCCTACAGTCAGATTACACTCGGACCTCGTCGACATGCATTGCTCAATTTTTCTTGCTCTGTGGGATTCATGGCCTCCTAAGTTTCGAGCTGGACTCAGTGGGCGTTGAGTTGGTCCCTTCGTTAG
ATCCTGGGTTCTGCCTACAGTCAGATTACACTCGGACCTCGTCGACATGCATTGCATGGCCCATCCCATCATCCAGGCCCTCCTAAGTTTCGAGCTGGACTCAGTGGGCGTTGAGTTGGTCCCTTCGTTAG
ATCCTGGGTTCTGCCTACAGTCAGATTACACTCGGACCTCGTCGACATGCATTTCAGGCAGAGAACTGCACCCTTCTCTGCCTCCTAAGTTTCGAGCTGGACTCAGTGGGCGTTGAGTTGGTCCCTTCGTTAG
ATCCTGGGTTCTGCCTACAGTCAGATTACACTCGGACCTCGTCGACATGCATTATTCATCTGACTGACCTTCCTGTTAGCAGGACCTCCTAAGTTTCGAGCTGGACTCAGTGGGCGTTGAGTTGGTCCCTTCGTTAG
ATCCTGGGTTCTGCCTACAGTCAGATTACACTCGGACCTCGTCGACATGCATTTGTTCTGTACCATGGTCTGGAGCCTCTCCCTCCTAAGTTTCGAGCTGGACTCAGTGGGCGTTGAGTTGGTCCCTTCGTTAG
ATCCTGGGTTCTGCCTACAGTCAGATTACACTCGGACCTCGTCGACATGCATTGTTTCTGACATGATGACTTCTTGGGCAGTTTGCCTCCTAAGTTTCGAGCTGGACTCAGTGGGCGTTGAGTTGGTCCCTTCGTTAG
ATCCTGGGTTCTGCCTACAGTCAGATTACACTCGGACCTCGTCGACATGCATTGGAACTCAGGGCTTTCTGACAAGAGCTGTCCTCCTAAGTTTCGAGCTGGACTCAGTGGGCGTTGAGTTGGTCCCTTCGTTAG
ATCCTGGGTTCTGCCTACAGTCAGATTACACTCGGACCTCGTCGACATGCATTTGTGATGAACTTGGAAAGTGCCCTGCACCCCTCCTAAGTTTCGAGCTGGACTCAGTGGGCGTTGAGTTGGTCCCTTCGTTAG
ATCCTGGGTTCTGCCTACAGTCAGATTACACTCGGACCTCGTCGACATGCATTAGTAAGTCTTCCACCAACTGCTGGCGGACCTCCTAAGTTTCGAGCTGGACTCAGTGGGCGTTGAGTTGGTCCCTTCGTTAG
ATCCTGGGTTCTGCCTACAGTCAGATTACACTCGGACCTCGTCGACATGCATTCCTCATGCCTGCAAGTGGCCTCATGATGCCTCCTAAGTTTCGAGCTGGACTCAGTGGGCGTTGAGTTGGTCCCTTCGTTAG
ATCCTGGGTTCTGCCTACAGTCAGATTACACTCGGACCTCGTCGACATGCATTGTCAGATTCCTAACTATATTACTGAGGCTGGCCCTCCTAAGTTTCGAGCTGGACTCAGTGGGCGTTGAGTTGGTCCCTTCGTTAG
ATCCTGGGTTCTGCCTACAGTCAGATTACACTCGGACCTCGTCGACATGCATTTACTTCCAACCACTGAGTTCTCCATGAGCTCCTCCTAAGTTTCGAGCTGGACTCAGTGGGCGTTGAGTTGGTCCCTTCGTTAG
ATCCTGGGTTCTGCCTACAGTCAGATTACACTCGGACCTCGTCGACATGCATTCTCATCCAGTTTTACTTGTTTCTTTTTCAGGCCTTCCTCCTAAGTTTCGAGCTGGACTCAGTGGGCGTTGAGTTGGTCCCTTCGTTAG
ATCCTGGGTTCTGCCTACAGTCAGATTACACTCGGACCTCGTCGACATGCATTTTCGGAGGTCCTGTTTTCAAGTTCCTCCTGCCTCCTAAGTTTCGAGCTGGACTCAGTGGGCGTTGAGTTGGTCCCTTCGTTAG
ATCCTGGGTTCTGCCTACAGTCAGATTACACTCGGACCTCGTCGACATGCATTACCTGATGGGTTTAGATGTTTTTGGTGCTTGGCCTCCTAAGTTTCGAGCTGGACTCAGTGGGCGTTGAGTTGGTCCCTTCGTTAG
ATCCTGGGTTCTGCCTACAGTCAGATTACACTCGGACCTCGTCGACATGCATTTATGAGTGTACCCTTTGGTTTGGCTATGACCCCTCCTAAGTTTCGAGCTGGACTCAGTGGGCGTTGAGTTGGTCCCTTCGTTAG
ATCCTGGGTTCTGCCTACAGTCAGATTACACTCGGACCTCGTCGACATGCATTCTGCTTGCCTGCCTGCTGCAGGATCTCCTCCTAAGTTTCGAGCTGGACTCAGTGGGCGTTGAGTTGGTCCCTTCGTTAG
ATCCTGGGTTCTGCCTACAGTCAGATTACACTCGGACCTCGTCGACATGCATTTCTTGTAGTGAGGCCTCCTGGGTTGACCCTCCTAAGTTTCGAGCTGGACTCAGTGGGCGTTGAGTTGGTCCCTTCGTTAG
ATCCTGGGTTCTGCCTACAGTCAGATTACACTCGGACCTCGTCGACATGCATTTCTTGAGCATGAGTGGAAAGTCCTATTTGGCCCTCCTAAGTTTCGAGCTGGACTCAGTGGGCGTTGAGTTGGTCCCTTCGTTAG
ATCCTGGGTTCTGCCTACAGTCAGATTACACTCGGACCTCGTCGACATGCATTCGCATGGTGATCATCCACCTCCGAGGCCCTCCTAAGTTTCGAGCTGGACTCAGTGGGCGTTGAGTTGGTCCCTTCGTTAG
ATCCTGGGTTCTGCCTACAGTCAGATTACACTCGGACCTCGTCGACATGCATTATCTGACCGTAGCTTCTCTTTTTCTCTGACCACCTCCTAAGTTTCGAGCTGGACTCAGTGGGCGTTGAGTTGGTCCCTTCGTTAG
ATCCTGGGTTCTGCCTACAGTCAGATTACACTCGGACCTCGTCGACATGCATTCTCTCTCTCTTTTCGGAGTTCATTTTTAAGGCTGCCTCCTAAGTTTCGAGCTGGACTCAGTGGGCGTTGAGTTGGTCCCTTCGTTAG
ATCCTGGGTTCTGCCTACAGTCAGATTACACTCGGACCTCGTCGACATGCATTTGTTCCTGGTTTCCCTGTGGTGTGTTGGCCTCCTAAGTTTCGAGCTGGACTCAGTGGGCGTTGAGTTGGTCCCTTCGTTAG
ATCCTGGGTTCTGCCTACAGTCAGATTACACTCGGACCTCGTCGACATGCATTGAGGTTGTACTCATTCCGCCGCTCTATGGCCTCCTAAGTTTCGAGCTGGACTCAGTGGGCGTTGAGTTGGTCCCTTCGTTAG
ATCCTGGGTTCTGCCTACAGTCAGATTACACTCGGACCTCGTCGACATGCATTCTTCACCATTGCGTCTGCCTTAGTGTTCTTGCCTCCTAAGTTTCGAGCTGGACTCAGTGGGCGTTGAGTTGGTCCCTTCGTTAG
ATCCTGGGTTCTGCCTACAGTCAGATTACACTCGGACCTCGTCGACATGCATTAGGTGCTGGTTTATCCTTTCTGCCTGCAGCCTCCTAAGTTTCGAGCTGGACTCAGTGGGCGTTGAGTTGGTCCCTTCGTTAG

ATCCTGGGTTCTGCCTACAGTCAGATTACACTCGGACCTCGTCGACATGCATTTCTATCATTCGTTCCTCCATGACAGTTACCAGCCTCCTAAGTTTCGAGCTGGACTCAGTGGGCGTTGAGTTGGTCCCTTCGTTAG
ATCCTGGGTTCTGCCTACAGTCAGATTACACTCGGACCTCGTCGACATGCATTCTGTTTTCTGTCGCTGTTCAGTCACTTCCAGCCTCCTAAGTTTCGAGCTGGACTCAGTGGGCGTTGAGTTGGTCCCTTCGTTAG
ATCCTGGGTTCTGCCTACAGTCAGATTACACTCGGACCTCGTCGACATGCATTAGAGTTGTTCTTTGAGCTGATTCACTTTCTCTTGCCTCCTAAGTTTCGAGCTGGACTCAGTGGGCGTTGAGTTGGTCCCTTCGTTAG

NEK9
GTCCCTAATTGCCTCTCGGATTCCT TTACACTCGGACCTCGTCGACATGGTTACTGCAGGATTCAGCCGAGGTGACGACCTCCTAAGTTTCGAGCTGGACTCAGTGGATCTGGCCTGTGAGTGTTGGTGTG
GTCCCTAATTGCCTCTCGGATTCCT TTACACTCGGACCTCGTCGACATGAGACCCTGATCACTTGCTTCCCACCAAGGCCTCCTAAGTTTCGAGCTGGACTCAGTGGATCTGGCCTGTGAGTGTTGGTGTG
GTCCCTAATTGCCTCTCGGATTCCT TTACACTCGGACCTCGTCGACATGTTGGCCAAGGTAAAGGACGTTGTGTAGGGACCTCCTAAGTTTCGAGCTGGACTCAGTGGATCTGGCCTGTGAGTGTTGGTGTG
GTCCCTAATTGCCTCTCGGATTCCT TTACACTCGGACCTCGTCGACATGATTCATTGAGTCCACAGGCCAGCACTTTGCCCTCCTAAGTTTCGAGCTGGACTCAGTGGATCTGGCCTGTGAGTGTTGGTGTG
GTCCCTAATTGCCTCTCGGATTCCT TTACACTCGGACCTCGTCGACATGTCCTCTTCTGAATCCAAACCCAGTCGTCCACCTCCTAAGTTTCGAGCTGGACTCAGTGGATCTGGCCTGTGAGTGTTGGTGTG
GTCCCTAATTGCCTCTCGGATTCCT TTACACTCGGACCTCGTCGACATGTCCACTGGATTGCTGAGGAAGAAGTTCAGCCCTCCTAAGTTTCGAGCTGGACTCAGTGGATCTGGCCTGTGAGTGTTGGTGTG
GTCCCTAATTGCCTCTCGGATTCCT TTACACTCGGACCTCGTCGACATGCATGGGTTCTAGCACTTCAGGGCCAGCAACCTCCTAAGTTTCGAGCTGGACTCAGTGGATCTGGCCTGTGAGTGTTGGTGTG
GTCCCTAATTGCCTCTCGGATTCCT TTACACTCGGACCTCGTCGACATGCTACAGCAATGGGTGCTTCAGTCACAGTGCCCTCCTAAGTTTCGAGCTGGACTCAGTGGATCTGGCCTGTGAGTGTTGGTGTG
GTCCCTAATTGCCTCTCGGATTCCT TTACACTCGGACCTCGTCGACATGAGAGTACTGGCTAGAGTCAACTTCCATGGCCCTCCTAAGTTTCGAGCTGGACTCAGTGGATCTGGCCTGTGAGTGTTGGTGTG
GTCCCTAATTGCCTCTCGGATTCCT TTACACTCGGACCTCGTCGACATGAGCTCTGGAGACATGTAATATGGGGTTCCCCCTCCTAAGTTTCGAGCTGGACTCAGTGGATCTGGCCTGTGAGTGTTGGTGTG
GTCCCTAATTGCCTCTCGGATTCCT TTACACTCGGACCTCGTCGACATGTCAAGGCATCACGACGTTCCTTCTCAGACACCTCCTAAGTTTCGAGCTGGACTCAGTGGATCTGGCCTGTGAGTGTTGGTGTG
GTCCCTAATTGCCTCTCGGATTCCT TTACACTCGGACCTCGTCGACATGTCGACTTCCTTCCACACAACCAGTGAGTCACCTCCTAAGTTTCGAGCTGGACTCAGTGGATCTGGCCTGTGAGTGTTGGTGTG
GTCCCTAATTGCCTCTCGGATTCCT TTACACTCGGACCTCGTCGACATGCTAGAGGCTGGGTCTACAGGAGTCTGTTCCCTCCTAAGTTTCGAGCTGGACTCAGTGGATCTGGCCTGTGAGTGTTGGTGTG
GTCCCTAATTGCCTCTCGGATTCCT TTACACTCGGACCTCGTCGACATGTGCTGTCTGAGTTCCTTTGGAATGCATCCCCCTCCTAAGTTTCGAGCTGGACTCAGTGGATCTGGCCTGTGAGTGTTGGTGTG
GTCCCTAATTGCCTCTCGGATTCCT TTACACTCGGACCTCGTCGACATGTGCTGTAGCTTCTGTTGTTCAGCCAGACACCCTCCTAAGTTTCGAGCTGGACTCAGTGGATCTGGCCTGTGAGTGTTGGTGTG
GTCCCTAATTGCCTCTCGGATTCCT TTACACTCGGACCTCGTCGACATGGGGGTTTGTGTTCCAAAGGAGCTTCAGAGGCCTCCTAAGTTTCGAGCTGGACTCAGTGGATCTGGCCTGTGAGTGTTGGTGTG
GTCCCTAATTGCCTCTCGGATTCCT TTACACTCGGACCTCGTCGACATGACTTTGAGTCCTTGCAGCTCTTCATAGGGCCCTCCTAAGTTTCGAGCTGGACTCAGTGGATCTGGCCTGTGAGTGTTGGTGTG
GTCCCTAATTGCCTCTCGGATTCCT TTACACTCGGACCTCGTCGACATGCTGCATTTTCCAGCTCCTTTCGAAGCCAGCCCTCCTAAGTTTCGAGCTGGACTCAGTGGATCTGGCCTGTGAGTGTTGGTGTG
GTCCCTAATTGCCTCTCGGATTCCT TTACACTCGGACCTCGTCGACATGTGGCCTCTGTGGGACTGATTAAACCTTCCACCTCCTAAGTTTCGAGCTGGACTCAGTGGATCTGGCCTGTGAGTGTTGGTGTG
GTCCCTAATTGCCTCTCGGATTCCT TTACACTCGGACCTCGTCGACATGCCTCGGTCTGCTTCCATTGTTCCTCGGAAGCCTCCTAAGTTTCGAGCTGGACTCAGTGGATCTGGCCTGTGAGTGTTGGTGTG
GTCCCTAATTGCCTCTCGGATTCCT TTACACTCGGACCTCGTCGACATGCCACTTGGGTCAGGAGTTTCAGATTCCTGCCCTCCTAAGTTTCGAGCTGGACTCAGTGGATCTGGCCTGTGAGTGTTGGTGTG
GTCCCTAATTGCCTCTCGGATTCCT TTACACTCGGACCTCGTCGACATGGCCATGTGGTCTCTCTGTGGGGGTCATTCCTCCTAAGTTTCGAGCTGGACTCAGTGGATCTGGCCTGTGAGTGTTGGTGTG
GTCCCTAATTGCCTCTCGGATTCCT TTACACTCGGACCTCGTCGACATGTCAATAGCAGCTGTGTGAGTCTTGCCTGGGCCTCCTAAGTTTCGAGCTGGACTCAGTGGATCTGGCCTGTGAGTGTTGGTGTG
GTCCCTAATTGCCTCTCGGATTCCT TTACACTCGGACCTCGTCGACATGATTCCCGACATGCACTGATTCAGACCCAGCCCTCCTAAGTTTCGAGCTGGACTCAGTGGATCTGGCCTGTGAGTGTTGGTGTG
GTCCCTAATTGCCTCTCGGATTCCT TTACACTCGGACCTCGTCGACATGAAGGCCTTGGGAACATCCACCTTTTGTGGTCCTCCTAAGTTTCGAGCTGGACTCAGTGGATCTGGCCTGTGAGTGTTGGTGTG
GTCCCTAATTGCCTCTCGGATTCCT TTACACTCGGACCTCGTCGACATGTTCCACATGCTTTGGCTGTCGATAGGAGGCCCTCCTAAGTTTCGAGCTGGACTCAGTGGATCTGGCCTGTGAGTGTTGGTGTG
GTCCCTAATTGCCTCTCGGATTCCT TTACACTCGGACCTCGTCGACATGTGTCTCCATGGCCCAGCTGACCATGGACCTCCTAAGTTTCGAGCTGGACTCAGTGGATCTGGCCTGTGAGTGTTGGTGTG
GTCCCTAATTGCCTCTCGGATTCCT TTACACTCGGACCTCGTCGACATGTCCTTCTCCACTGTGACCACAGCAAAGTGGCCTCCTAAGTTTCGAGCTGGACTCAGTGGATCTGGCCTGTGAGTGTTGGTGTG
GTCCCTAATTGCCTCTCGGATTCCT TTACACTCGGACCTCGTCGACATGACATCCAGTTTCTGGGGGGTGGATTTTCCACCTCCTAAGTTTCGAGCTGGACTCAGTGGATCTGGCCTGTGAGTGTTGGTGTG
GTCCCTAATTGCCTCTCGGATTCCT TTACACTCGGACCTCGTCGACATGAGTTCATCTGCAGTAGGTCTCTGCTCAGGACCTCCTAAGTTTCGAGCTGGACTCAGTGGATCTGGCCTGTGAGTGTTGGTGTG
GTCCCTAATTGCCTCTCGGATTCCT TTACACTCGGACCTCGTCGACATGTCCAGCTTTATGGATGCAGCTCACTGCTGACCTCCTAAGTTTCGAGCTGGACTCAGTGGATCTGGCCTGTGAGTGTTGGTGTG
GTCCCTAATTGCCTCTCGGATTCCT TTACACTCGGACCTCGTCGACATGTCCAGCTCAATCAGCAGCGTGGTATTGTCCCCTCCTAAGTTTCGAGCTGGACTCAGTGGATCTGGCCTGTGAGTGTTGGTGTG
GTCCCTAATTGCCTCTCGGATTCCT TTACACTCGGACCTCGTCGACATGGGCAATAATGTTGTCGTGCTGCAGCAGTGCCCTCCTAAGTTTCGAGCTGGACTCAGTGGATCTGGCCTGTGAGTGTTGGTGTG
GTCCCTAATTGCCTCTCGGATTCCT TTACACTCGGACCTCGTCGACATGCGAGTTGATGGAATCGCAGTGTCGCTCGTACCTCCTAAGTTTCGAGCTGGACTCAGTGGATCTGGCCTGTGAGTGTTGGTGTG
GTCCCTAATTGCCTCTCGGATTCCT TTACACTCGGACCTCGTCGACATGAGGCACCAGGAATCTGAATCTAAGTCAGGCCCTCCTAAGTTTCGAGCTGGACTCAGTGGATCTGGCCTGTGAGTGTTGGTGTG
GTCCCTAATTGCCTCTCGGATTCCT TTACACTCGGACCTCGTCGACATGTAACACCAGACCCTGCAATCTCTCAACCTCCCTCCTAAGTTTCGAGCTGGACTCAGTGGATCTGGCCTGTGAGTGTTGGTGTG
GTCCCTAATTGCCTCTCGGATTCCT TTACACTCGGACCTCGTCGACATGGACAGGAGCTGCTGGCCCCATTACTGTTCCTCCTAAGTTTCGAGCTGGACTCAGTGGATCTGGCCTGTGAGTGTTGGTGTG
GTCCCTAATTGCCTCTCGGATTCCT TTACACTCGGACCTCGTCGACATGCAACGATGAGAATGGTATGCCATCCACGGCCCTCCTAAGTTTCGAGCTGGACTCAGTGGATCTGGCCTGTGAGTGTTGGTGTG
GTCCCTAATTGCCTCTCGGATTCCT TTACACTCGGACCTCGTCGACATGACAGGTCCGGGACATGATGCAGAGATCCACCTCCTAAGTTTCGAGCTGGACTCAGTGGATCTGGCCTGTGAGTGTTGGTGTG
GTCCCTAATTGCCTCTCGGATTCCT TTACACTCGGACCTCGTCGACATGATAGGCCGAGGCCATGAGGTACAGATATCACCTCCTAAGTTTCGAGCTGGACTCAGTGGATCTGGCCTGTGAGTGTTGGTGTG
GTCCCTAATTGCCTCTCGGATTCCT TTACACTCGGACCTCGTCGACATGCAGTGGCAGCAATGGTAAACTCATCACCGCCCTCCTAAGTTTCGAGCTGGACTCAGTGGATCTGGCCTGTGAGTGTTGGTGTG
GTCCCTAATTGCCTCTCGGATTCCT TTACACTCGGACCTCGTCGACATGACTGGGTCAACAGAAATGTCCCATCACAGCCCTCCTAAGTTTCGAGCTGGACTCAGTGGATCTGGCCTGTGAGTGTTGGTGTG
GTCCCTAATTGCCTCTCGGATTCCT TTACACTCGGACCTCGTCGACATGTTCGCCACAGCCCCAAGAATAGACTTCCTTCCTCCTAAGTTTCGAGCTGGACTCAGTGGATCTGGCCTGTGAGTGTTGGTGTG
GTCCCTAATTGCCTCTCGGATTCCT TTACACTCGGACCTCGTCGACATGCGAAGGCATAGAGCTGACCCTCATCAGTCACCTCCTAAGTTTCGAGCTGGACTCAGTGGATCTGGCCTGTGAGTGTTGGTGTG
GTCCCTAATTGCCTCTCGGATTCCT TTACACTCGGACCTCGTCGACATGTAGTGCCTCCTTGCATGTTCACCCAAGTGTCCTCCTAAGTTTCGAGCTGGACTCAGTGGATCTGGCCTGTGAGTGTTGGTGTG
GTCCCTAATTGCCTCTCGGATTCCT TTACACTCGGACCTCGTCGACATGTTTCCTCCATCTCTCTCCTGCGTTTCCTGACCTCCTAAGTTTCGAGCTGGACTCAGTGGATCTGGCCTGTGAGTGTTGGTGTG
GTCCCTAATTGCCTCTCGGATTCCT TTACACTCGGACCTCGTCGACATGTCTGAAAACGCTGCACTGAGAGGAGATGGGCCTCCTAAGTTTCGAGCTGGACTCAGTGGATCTGGCCTGTGAGTGTTGGTGTG
GTCCCTAATTGCCTCTCGGATTCCT TTACACTCGGACCTCGTCGACATGACCACCACATGATTATCTCCACAGGAGACCCCTCCTAAGTTTCGAGCTGGACTCAGTGGATCTGGCCTGTGAGTGTTGGTGTG
GTCCCTAATTGCCTCTCGGATTCCT TTACACTCGGACCTCGTCGACATGTCATCACCACATGACACCTGACGGATAGCTCCTCCTAAGTTTCGAGCTGGACTCAGTGGATCTGGCCTGTGAGTGTTGGTGTG
GTCCCTAATTGCCTCTCGGATTCCT TTACACTCGGACCTCGTCGACATGGCAGTTCAAAAATGACGCAGCCAACTGCCCCCTCCTAAGTTTCGAGCTGGACTCAGTGGATCTGGCCTGTGAGTGTTGGTGTG
GTCCCTAATTGCCTCTCGGATTCCT TTACACTCGGACCTCGTCGACATGGGTACCACACCACCATCTCTTCCTCAAACACCTCCTAAGTTTCGAGCTGGACTCAGTGGATCTGGCCTGTGAGTGTTGGTGTG
GTCCCTAATTGCCTCTCGGATTCCT TTACACTCGGACCTCGTCGACATGGTCCACCCCCATGCAGCCATAATAATCTGACCTCCTAAGTTTCGAGCTGGACTCAGTGGATCTGGCCTGTGAGTGTTGGTGTG

CHD3
GTCTCCCGCCGCACAGTCTCTAATG TTACACTCGGACCTCGTCGACATGGTGAAGCTCTGTGAAGCCACCATCGGCGACCTCCTAAGTTTCGAGCTGGACTCAGTGCAGAGACTAGCCTCCAGTACCCACC
GTCTCCCGCCGCACAGTCTCTAATG TTACACTCGGACCTCGTCGACATGCAGAAGCCTCAGGAGTGGTGGGAGACGTTCCTCCTAAGTTTCGAGCTGGACTCAGTGCAGAGACTAGCCTCCAGTACCCACC
GTCTCCCGCCGCACAGTCTCTAATG TTACACTCGGACCTCGTCGACATGTTGACGAGAGACATGACTCCAATGCGGGTCCCTCCTAAGTTTCGAGCTGGACTCAGTGCAGAGACTAGCCTCCAGTACCCACC
GTCTCCCGCCGCACAGTCTCTAATG TTACACTCGGACCTCGTCGACATGACAGCATTGAGGAAAGCCTTCCGCTGACGGCCTCCTAAGTTTCGAGCTGGACTCAGTGCAGAGACTAGCCTCCAGTACCCACC
GTCTCCCGCCGCACAGTCTCTAATG TTACACTCGGACCTCGTCGACATGTTGGTCATCTGCGAGAAGATGAGCACTCGGCCTCCTAAGTTTCGAGCTGGACTCAGTGCAGAGACTAGCCTCCAGTACCCACC
GTCTCCCGCCGCACAGTCTCTAATG TTACACTCGGACCTCGTCGACATGTATTAAGCAGCGACACCTGGTTCCCACCACCCTCCTAAGTTTCGAGCTGGACTCAGTGCAGAGACTAGCCTCCAGTACCCACC
GTCTCCCGCCGCACAGTCTCTAATG TTACACTCGGACCTCGTCGACATGAAGACATCTGCCTTGAGTCTCCGCAGCATGCCTCCTAAGTTTCGAGCTGGACTCAGTGCAGAGACTAGCCTCCAGTACCCACC
GTCTCCCGCCGCACAGTCTCTAATG TTACACTCGGACCTCGTCGACATGAATCTCTCTGGGGTGAGGAAGTTCAGGAGACCTCCTAAGTTTCGAGCTGGACTCAGTGCAGAGACTAGCCTCCAGTACCCACC
GTCTCCCGCCGCACAGTCTCTAATG TTACACTCGGACCTCGTCGACATGCCAAGTGCTGCCTGATCAATGGTGATCAGCCCTCCTAAGTTTCGAGCTGGACTCAGTGCAGAGACTAGCCTCCAGTACCCACC
GTCTCCCGCCGCACAGTCTCTAATG TTACACTCGGACCTCGTCGACATGGTCAGGAGAACATGGAACTTCACCTGTGCCCCTCCTAAGTTTCGAGCTGGACTCAGTGCAGAGACTAGCCTCCAGTACCCACC
GTCTCCCGCCGCACAGTCTCTAATG TTACACTCGGACCTCGTCGACATGAGAATTTGGGTGCCCACATCTGGAACTCCCCCTCCTAAGTTTCGAGCTGGACTCAGTGCAGAGACTAGCCTCCAGTACCCACC
GTCTCCCGCCGCACAGTCTCTAATG TTACACTCGGACCTCGTCGACATGTCCCAGTTAATGATGGTAGAGAGTGGGGCACCTCCTAAGTTTCGAGCTGGACTCAGTGCAGAGACTAGCCTCCAGTACCCACC
GTCTCCCGCCGCACAGTCTCTAATG TTACACTCGGACCTCGTCGACATGCCAGGAGAAGCGTAGCCAGTTCAGCCCTTCCTCCTAAGTTTCGAGCTGGACTCAGTGCAGAGACTAGCCTCCAGTACCCACC
GTCTCCCGCCGCACAGTCTCTAATG TTACACTCGGACCTCGTCGACATGAACTGATACATGTGCAGGGTGCCTCCAGTGCCTCCTAAGTTTCGAGCTGGACTCAGTGCAGAGACTAGCCTCCAGTACCCACC
GTCTCCCGCCGCACAGTCTCTAATG TTACACTCGGACCTCGTCGACATGGTAGGATCATTAGTGGGAGAACTGGGAGGCCCTCCTAAGTTTCGAGCTGGACTCAGTGCAGAGACTAGCCTCCAGTACCCACC
GTCTCCCGCCGCACAGTCTCTAATG TTACACTCGGACCTCGTCGACATGGGAGCAGTGCCAGTAGGATAGTCCTACCCCCTCCTAAGTTTCGAGCTGGACTCAGTGCAGAGACTAGCCTCCAGTACCCACC
GTCTCCCGCCGCACAGTCTCTAATG TTACACTCGGACCTCGTCGACATGAGCCATTCACCATTGGGAATGTCAGGCAGGCCTCCTAAGTTTCGAGCTGGACTCAGTGCAGAGACTAGCCTCCAGTACCCACC
GTCTCCCGCCGCACAGTCTCTAATG TTACACTCGGACCTCGTCGACATGATGAATGTGGTAGGAGGAGATGCACGCGTCCCTCCTAAGTTTCGAGCTGGACTCAGTGCAGAGACTAGCCTCCAGTACCCACC
GTCTCCCGCCGCACAGTCTCTAATG TTACACTCGGACCTCGTCGACATGAGCAGCTGACACCTGCTCAGCTACAGCTCCTCCTAAGTTTCGAGCTGGACTCAGTGCAGAGACTAGCCTCCAGTACCCACC
GTCTCCCGCCGCACAGTCTCTAATG TTACACTCGGACCTCGTCGACATGTAGCAATTAGGGGCCTCATGAACTGGCTGACCTCCTAAGTTTCGAGCTGGACTCAGTGCAGAGACTAGCCTCCAGTACCCACC
GTCTCCCGCCGCACAGTCTCTAATG TTACACTCGGACCTCGTCGACATGCTGTGATGAAGGACCCTGCAGGCATCTGGCCTCCTAAGTTTCGAGCTGGACTCAGTGCAGAGACTAGCCTCCAGTACCCACC
GTCTCCCGCCGCACAGTCTCTAATG TTACACTCGGACCTCGTCGACATGAGCAACTCCTCCAGCTGGTTCAGAACCTTGCCTCCTAAGTTTCGAGCTGGACTCAGTGCAGAGACTAGCCTCCAGTACCCACC
GTCTCCCGCCGCACAGTCTCTAATG TTACACTCGGACCTCGTCGACATGTTGAGCATCATTCTGGATGTCCTGCCACCGCCTCCTAAGTTTCGAGCTGGACTCAGTGCAGAGACTAGCCTCCAGTACCCACC
GTCTCCCGCCGCACAGTCTCTAATG TTACACTCGGACCTCGTCGACATGTGAACCGGGGCTTCTCTGTCTTTTCCTCTCCCTCCTAAGTTTCGAGCTGGACTCAGTGCAGAGACTAGCCTCCAGTACCCACC
GTCTCCCGCCGCACAGTCTCTAATG TTACACTCGGACCTCGTCGACATGCCTGGTCGAAGCTCCCGGTCACCTTTTACACCTCCTAAGTTTCGAGCTGGACTCAGTGCAGAGACTAGCCTCCAGTACCCACC
GTCTCCCGCCGCACAGTCTCTAATG TTACACTCGGACCTCGTCGACATGTCGACTCTGTGGCTGACTTCTCTCTGTCCCCTCCTAAGTTTCGAGCTGGACTCAGTGCAGAGACTAGCCTCCAGTACCCACC
GTCTCCCGCCGCACAGTCTCTAATG TTACACTCGGACCTCGTCGACATGACGGTACCCAGGTTCAGGCTCCATCTCTCCCTCCTAAGTTTCGAGCTGGACTCAGTGCAGAGACTAGCCTCCAGTACCCACC
GTCTCCCGCCGCACAGTCTCTAATG TTACACTCGGACCTCGTCGACATGATGCCTTCTCCTTTCTCACTTGGAGCTGGACCTCCTAAGTTTCGAGCTGGACTCAGTGCAGAGACTAGCCTCCAGTACCCACC
GTCTCCCGCCGCACAGTCTCTAATG TTACACTCGGACCTCGTCGACATGAGCAGGTTTAGAGGTGCAGGGACTGTTGGTCCTCCTAAGTTTCGAGCTGGACTCAGTGCAGAGACTAGCCTCCAGTACCCACC
GTCTCCCGCCGCACAGTCTCTAATG TTACACTCGGACCTCGTCGACATGCGGCATTGACCAACGCCCATTGATGTGCTCCCTCCTAAGTTTCGAGCTGGACTCAGTGCAGAGACTAGCCTCCAGTACCCACC
GTCTCCCGCCGCACAGTCTCTAATG TTACACTCGGACCTCGTCGACATGTCTCATTCCGGAGCTGCCTCTTTGACTGTCCCTCCTAAGTTTCGAGCTGGACTCAGTGCAGAGACTAGCCTCCAGTACCCACC
GTCTCCCGCCGCACAGTCTCTAATG TTACACTCGGACCTCGTCGACATGTCGAAGTCTTCATCCTCCTCCTCTGAACCCCCTCCTAAGTTTCGAGCTGGACTCAGTGCAGAGACTAGCCTCCAGTACCCACC
GTCTCCCGCCGCACAGTCTCTAATG TTACACTCGGACCTCGTCGACATGCTCTGACTGGTTGTCTTGGTCTTCCTGAGCCCTCCTAAGTTTCGAGCTGGACTCAGTGCAGAGACTAGCCTCCAGTACCCACC
GTCTCCCGCCGCACAGTCTCTAATG TTACACTCGGACCTCGTCGACATGAGTTAACTTGCTTGCGAACCCGCTTGCCCTCCTCCTAAGTTTCGAGCTGGACTCAGTGCAGAGACTAGCCTCCAGTACCCACC
GTCTCCCGCCGCACAGTCTCTAATG TTACACTCGGACCTCGTCGACATGCCTAGATTCCGGGCTAGGTCTTCCTGCTGCCTCCTAAGTTTCGAGCTGGACTCAGTGCAGAGACTAGCCTCCAGTACCCACC
GTCTCCCGCCGCACAGTCTCTAATG TTACACTCGGACCTCGTCGACATGCTCATAGTGATGCCTCAGCAGCTTCTCCCACCTCCTAAGTTTCGAGCTGGACTCAGTGCAGAGACTAGCCTCCAGTACCCACC

GTCTCCCGCCGCACAGTCTCTAATG TTACACTCGGACCTCGTCGACATGGTCAGGGTCCACATTCTCCTCCTGCTTGATCCTCCTAAGTTTCGAGCTGGACTCAGTGCAGAGACTAGCCTCCAGTACCCACC
GTCTCCCGCCGCACAGTCTCTAATG TTACACTCGGACCTCGTCGACATGCTTGTCTTCTTCCCGCACGACGTACTGTGCCCTCCTAAGTTTCGAGCTGGACTCAGTGCAGAGACTAGCCTCCAGTACCCACC
GTCTCCCGCCGCACAGTCTCTAATG TTACACTCGGACCTCGTCGACATGGTTCTGCACGTCAGTGTCCTCAGTTGCATCCCTCCTAAGTTTCGAGCTGGACTCAGTGCAGAGACTAGCCTCCAGTACCCACC
GTCTCCCGCCGCACAGTCTCTAATG TTACACTCGGACCTCGTCGACATGATCACACTGCTGTCCTCCTCCTTGTTCTCCCCTCCTAAGTTTCGAGCTGGACTCAGTGCAGAGACTAGCCTCCAGTACCCACC
GTCTCCCGCCGCACAGTCTCTAATG TTACACTCGGACCTCGTCGACATGATGTCGTCAAGCTCCTGCTTGGACATGGAGCCTCCTAAGTTTCGAGCTGGACTCAGTGCAGAGACTAGCCTCCAGTACCCACC
GTCTCCCGCCGCACAGTCTCTAATG TTACACTCGGACCTCGTCGACATGTGGCCACTTGTGTGATTCGCTCTTCCACTGCCTCCTAAGTTTCGAGCTGGACTCAGTGCAGAGACTAGCCTCCAGTACCCACC
GTCTCCCGCCGCACAGTCTCTAATG TTACACTCGGACCTCGTCGACATGTCACTTTGTTGGCCTGGCCAATCCGATGAGCCTCCTAAGTTTCGAGCTGGACTCAGTGCAGAGACTAGCCTCCAGTACCCACC
GTCTCCCGCCGCACAGTCTCTAATG TTACACTCGGACCTCGTCGACATGCGGCTAAAGGCCTGGATGTCATTATGGGGGCCTCCTAAGTTTCGAGCTGGACTCAGTGCAGAGACTAGCCTCCAGTACCCACC
GTCTCCCGCCGCACAGTCTCTAATG TTACACTCGGACCTCGTCGACATGTTGTTGGGCCCCAGGAGCATTAAACCGATCCCTCCTAAGTTTCGAGCTGGACTCAGTGCAGAGACTAGCCTCCAGTACCCACC
GTCTCCCGCCGCACAGTCTCTAATG TTACACTCGGACCTCGTCGACATGTGCTCCTTCAGCTTTCGCAGCATCTTCTGGCCTCCTAAGTTTCGAGCTGGACTCAGTGCAGAGACTAGCCTCCAGTACCCACC
GTCTCCCGCCGCACAGTCTCTAATG TTACACTCGGACCTCGTCGACATGTTCCCAGACGACTTAATAAGTGCCCCACCCCCTCCTAAGTTTCGAGCTGGACTCAGTGCAGAGACTAGCCTCCAGTACCCACC
GTCTCCCGCCGCACAGTCTCTAATG TTACACTCGGACCTCGTCGACATGATTTCTTCTGCATGGGGCTTAGCTCCACCCCCTCCTAAGTTTCGAGCTGGACTCAGTGCAGAGACTAGCCTCCAGTACCCACC
GTCTCCCGCCGCACAGTCTCTAATG TTACACTCGGACCTCGTCGACATGACGATGAGCTCTGTCTTGGCTGGCATGTTCCCTCCTAAGTTTCGAGCTGGACTCAGTGCAGAGACTAGCCTCCAGTACCCACC
GTCTCCCGCCGCACAGTCTCTAATG TTACACTCGGACCTCGTCGACATGTATTCTGCAATGGGGTTCCTGTCAGCAGCACCTCCTAAGTTTCGAGCTGGACTCAGTGCAGAGACTAGCCTCCAGTACCCACC
GTCTCCCGCCGCACAGTCTCTAATG TTACACTCGGACCTCGTCGACATGTTGGACTGGTTGTTCTTGAGTCGATGGGCCCCTCCTAAGTTTCGAGCTGGACTCAGTGCAGAGACTAGCCTCCAGTACCCACC
GTCTCCCGCCGCACAGTCTCTAATG TTACACTCGGACCTCGTCGACATGTTCATTCTCACGAATGATGGCCCGGCTGTCCCTCCTAAGTTTCGAGCTGGACTCAGTGCAGAGACTAGCCTCCAGTACCCACC
GTCTCCCGCCGCACAGTCTCTAATG TTACACTCGGACCTCGTCGACATGCACCAGGAAGGGACCTTTTGTGTGGCCCTCCTCCTAAGTTTCGAGCTGGACTCAGTGCAGAGACTAGCCTCCAGTACCCACC
GTCTCCCGCCGCACAGTCTCTAATG TTACACTCGGACCTCGTCGACATGTCATCAGCTAGAATGGTGTCAGTGCCCTGGCCTCCTAAGTTTCGAGCTGGACTCAGTGCAGAGACTAGCCTCCAGTACCCACC
GTCTCCCGCCGCACAGTCTCTAATG TTACACTCGGACCTCGTCGACATGTCTTCTTCTTATACTTGCGGGGCTGGGCAGCCTCCTAAGTTTCGAGCTGGACTCAGTGCAGAGACTAGCCTCCAGTACCCACC
GTCTCCCGCCGCACAGTCTCTAATG TTACACTCGGACCTCGTCGACATGTACGCTTGTCGCTCTTCCCATCATCCTCGCCCTCCTAAGTTTCGAGCTGGACTCAGTGCAGAGACTAGCCTCCAGTACCCACC
GTCTCCCGCCGCACAGTCTCTAATG TTACACTCGGACCTCGTCGACATGTGGAAGATTTCCAGCTGAAGCTCCTTGGCCCCTCCTAAGTTTCGAGCTGGACTCAGTGCAGAGACTAGCCTCCAGTACCCACC
GTCTCCCGCCGCACAGTCTCTAATG TTACACTCGGACCTCGTCGACATGACTCTCGCTCTGATCTGCCTTGAAGAGGACCCTCCTAAGTTTCGAGCTGGACTCAGTGCAGAGACTAGCCTCCAGTACCCACC
GTCTCCCGCCGCACAGTCTCTAATG TTACACTCGGACCTCGTCGACATGGGGGGTGGGACATCTGGATTTCCATCTGCCCTCCTAAGTTTCGAGCTGGACTCAGTGCAGAGACTAGCCTCCAGTACCCACC
GTCTCCCGCCGCACAGTCTCTAATG TTACACTCGGACCTCGTCGACATGCGGCAGTACTCCATGTGATCATCCTCCTCCCCTCCTAAGTTTCGAGCTGGACTCAGTGCAGAGACTAGCCTCCAGTACCCACC
GTCTCCCGCCGCACAGTCTCTAATG TTACACTCGGACCTCGTCGACATGTTCTTCTTCTTCCTCCTTGGCCTCCCACTGCCTCCTAAGTTTCGAGCTGGACTCAGTGCAGAGACTAGCCTCCAGTACCCACC
GTCTCCCGCCGCACAGTCTCTAATG TTACACTCGGACCTCGTCGACATGAGAATAATTTCCCCACCCTGCTGGCACACCCCTCCTAAGTTTCGAGCTGGACTCAGTGCAGAGACTAGCCTCCAGTACCCACC
GTCTCCCGCCGCACAGTCTCTAATG TTACACTCGGACCTCGTCGACATGACAGTAATCCTGGTGATCCGTCTCGTAGCCCCTCCTAAGTTTCGAGCTGGACTCAGTGCAGAGACTAGCCTCCAGTACCCACC
GTCTCCCGCCGCACAGTCTCTAATG TTACACTCGGACCTCGTCGACATGCTGCCACTGTCCAGGTCTGATTCCTCAGCCCTCCTAAGTTTCGAGCTGGACTCAGTGCAGAGACTAGCCTCCAGTACCCACC
GTCTCCCGCCGCACAGTCTCTAATG TTACACTCGGACCTCGTCGACATGATACGAGCCTCCTTTCTTCCTCTTGCCACCCCTCCTAAGTTTCGAGCTGGACTCAGTGCAGAGACTAGCCTCCAGTACCCACC
GTCTCCCGCCGCACAGTCTCTAATG TTACACTCGGACCTCGTCGACATGTGTTGGCACTGAACTCTCTCCATTTGGCCCCCTCCTAAGTTTCGAGCTGGACTCAGTGCAGAGACTAGCCTCCAGTACCCACC
GTCTCCCGCCGCACAGTCTCTAATG TTACACTCGGACCTCGTCGACATGAGTTGGTGAGCGTGTGGTAATCCTCCTCAGCCTCCTAAGTTTCGAGCTGGACTCAGTGCAGAGACTAGCCTCCAGTACCCACC
GTCTCCCGCCGCACAGTCTCTAATG TTACACTCGGACCTCGTCGACATGTCTCGGTGCTTCCTTCTTCGTTTCCGACCCCCTCCTAAGTTTCGAGCTGGACTCAGTGCAGAGACTAGCCTCCAGTACCCACC
GTCTCCCGCCGCACAGTCTCTAATG TTACACTCGGACCTCGTCGACATGTCACTGTCACGCTTCTTGCGTTTTCGGGGTCCTCCTAAGTTTCGAGCTGGACTCAGTGCAGAGACTAGCCTCCAGTACCCACC
GTCTCCCGCCGCACAGTCTCTAATG TTACACTCGGACCTCGTCGACATGTCCTGGCTTGTTCTCCTTCTGCTTCTTGGGCCTCCTAAGTTTCGAGCTGGACTCAGTGCAGAGACTAGCCTCCAGTACCCACC
GTCTCCCGCCGCACAGTCTCTAATG TTACACTCGGACCTCGTCGACATGTCTCTTCTTCACACCCAATGCTGACGGCAGCCTCCTAAGTTTCGAGCTGGACTCAGTGCAGAGACTAGCCTCCAGTACCCACC
GTCTCCCGCCGCACAGTCTCTAATG TTACACTCGGACCTCGTCGACATGACTTCTTGCCCACAGGATCACAGTGTCTGCCCTCCTAAGTTTCGAGCTGGACTCAGTGCAGAGACTAGCCTCCAGTACCCACC
GTCTCCCGCCGCACAGTCTCTAATG TTACACTCGGACCTCGTCGACATGCAGTCGTCTATACAGATCACCTCCCCGGCCCTCCTAAGTTTCGAGCTGGACTCAGTGCAGAGACTAGCCTCCAGTACCCACC
GTCTCCCGCCGCACAGTCTCTAATG TTACACTCGGACCTCGTCGACATGCATAGCCATGGAGGACAATCCCAGCCAGAACCTCCTAAGTTTCGAGCTGGACTCAGTGCAGAGACTAGCCTCCAGTACCCACC
GTCTCCCGCCGCACAGTCTCTAATG TTACACTCGGACCTCGTCGACATGTGTGCCAGATCTCATTGAGTTTCCCCGAGGCCTCCTAAGTTTCGAGCTGGACTCAGTGCAGAGACTAGCCTCCAGTACCCACC
GTCTCCCGCCGCACAGTCTCTAATG TTACACTCGGACCTCGTCGACATGATAGCTGCCCGTTCCTCATTCTGCCACAGTCCTCCTAAGTTTCGAGCTGGACTCAGTGCAGAGACTAGCCTCCAGTACCCACC
GTCTCCCGCCGCACAGTCTCTAATG TTACACTCGGACCTCGTCGACATGTTCTCTCTTGCCCAAATCCCCTGTTTCCCCCCTCCTAAGTTTCGAGCTGGACTCAGTGCAGAGACTAGCCTCCAGTACCCACC
GTCTCCCGCCGCACAGTCTCTAATG TTACACTCGGACCTCGTCGACATGTCCTCCTGGTTTTCAGCTTCCTCCTTCTCACCTCCTAAGTTTCGAGCTGGACTCAGTGCAGAGACTAGCCTCCAGTACCCACC
GTCTCCCGCCGCACAGTCTCTAATG TTACACTCGGACCTCGTCGACATGAGGCCTTAAACTCCTTCTCAGTCTTGCCCCCCTCCTAAGTTTCGAGCTGGACTCAGTGCAGAGACTAGCCTCCAGTACCCACC
GTCTCCCGCCGCACAGTCTCTAATG TTACACTCGGACCTCGTCGACATGGTTGAAGCCCAGCACCTCAATGTTGCCCCCCTCCTAAGTTTCGAGCTGGACTCAGTGCAGAGACTAGCCTCCAGTACCCACC
GTCTCCCGCCGCACAGTCTCTAATG TTACACTCGGACCTCGTCGACATGAAGATGATGACAGTGTCAGCAGTGGCCAGACCTCCTAAGTTTCGAGCTGGACTCAGTGCAGAGACTAGCCTCCAGTACCCACC
GTCTCCCGCCGCACAGTCTCTAATG TTACACTCGGACCTCGTCGACATGGGATATGTCAGCAAACTCCTCCAGGAAGCCCCTCCTAAGTTTCGAGCTGGACTCAGTGCAGAGACTAGCCTCCAGTACCCACC
GTCTCCCGCCGCACAGTCTCTAATG TTACACTCGGACCTCGTCGACATGATCTACCACAAGACAGGCCCAGCGGATGGCCTCCTAAGTTTCGAGCTGGACTCAGTGCAGAGACTAGCCTCCAGTACCCACC
GTCTCCCGCCGCACAGTCTCTAATG TTACACTCGGACCTCGTCGACATGGCTTTCTTGCCCCCTTTGATGGCATTGTCCCCTCCTAAGTTTCGAGCTGGACTCAGTGCAGAGACTAGCCTCCAGTACCCACC
GTCTCCCGCCGCACAGTCTCTAATG TTACACTCGGACCTCGTCGACATGACGATGGTTTGTATGGTCTTGCCTAGCCCCCCTCCTAAGTTTCGAGCTGGACTCAGTGCAGAGACTAGCCTCCAGTACCCACC
GTCTCCCGCCGCACAGTCTCTAATG TTACACTCGGACCTCGTCGACATGATCTTCCTCCCACGTGGACTGGTCATATGGCCTCCTAAGTTTCGAGCTGGACTCAGTGCAGAGACTAGCCTCCAGTACCCACC
GTCTCCCGCCGCACAGTCTCTAATG TTACACTCGGACCTCGTCGACATGATCCACACTGTGGTTGATGATGCGGTGGACCCTCCTAAGTTTCGAGCTGGACTCAGTGCAGAGACTAGCCTCCAGTACCCACC
GTCTCCCGCCGCACAGTCTCTAATG TTACACTCGGACCTCGTCGACATGTACTTCTCCTCCATCTCAGCATAGTGCGGGCCTCCTAAGTTTCGAGCTGGACTCAGTGCAGAGACTAGCCTCCAGTACCCACC
GTCTCCCGCCGCACAGTCTCTAATG TTACACTCGGACCTCGTCGACATGCAGCCCAGGACCTTCTTCTTCTTCCTTCCTCCTCCTAAGTTTCGAGCTGGACTCAGTGCAGAGACTAGCCTCCAGTACCCACC
GTCTCCCGCCGCACAGTCTCTAATG TTACACTCGGACCTCGTCGACATGCCGGCCTCTCTTTAGTTTCTTGGTGCGGACCCTCCTAAGTTTCGAGCTGGACTCAGTGCAGAGACTAGCCTCCAGTACCCACC
GTCTCCCGCCGCACAGTCTCTAATG TTACACTCGGACCTCGTCGACATGGCCCTCTTTGGTTTTGGCTCTTCGGATGGGCCTCCTAAGTTTCGAGCTGGACTCAGTGCAGAGACTAGCCTCCAGTACCCACC
GTCTCCCGCCGCACAGTCTCTAATG TTACACTCGGACCTCGTCGACATGTGACTTCTGTTCCACTTGCTTTTGCCCCCCCCTCCTAAGTTTCGAGCTGGACTCAGTGCAGAGACTAGCCTCCAGTACCCACC
GTCTCCCGCCGCACAGTCTCTAATG TTACACTCGGACCTCGTCGACATGTCCGGTTCCATATTCACTGCCCCCACTCTCCTCCTAAGTTTCGAGCTGGACTCAGTGCAGAGACTAGCCTCCAGTACCCACC
GTCTCCCGCCGCACAGTCTCTAATG TTACACTCGGACCTCGTCGACATGGACTTCTCCCGGTACTCATCTCGCTCAGAACCTCCTAAGTTTCGAGCTGGACTCAGTGCAGAGACTAGCCTCCAGTACCCACC
GTCTCCCGCCGCACAGTCTCTAATG TTACACTCGGACCTCGTCGACATGTGTCATCCTTATCAGGCATCCTGTCACCCCCCTCCTAAGTTTCGAGCTGGACTCAGTGCAGAGACTAGCCTCCAGTACCCACC
GTCTCCCGCCGCACAGTCTCTAATG TTACACTCGGACCTCGTCGACATGACCAATGCCCCCACCATTTCCTTCTCCTTCCCTCCTAAGTTTCGAGCTGGACTCAGTGCAGAGACTAGCCTCCAGTACCCACC
GTCTCCCGCCGCACAGTCTCTAATG TTACACTCGGACCTCGTCGACATGTCTCCCCAATTCTGCTGTTCTTCTCTGGCTCCTCCTAAGTTTCGAGCTGGACTCAGTGCAGAGACTAGCCTCCAGTACCCACC
GTCTCCCGCCGCACAGTCTCTAATG TTACACTCGGACCTCGTCGACATGCCCCCACCGCCAATGTAGGATCTTCTGCACCTCCTAAGTTTCGAGCTGGACTCAGTGCAGAGACTAGCCTCCAGTACCCACC
GTCTCCCGCCGCACAGTCTCTAATG TTACACTCGGACCTCGTCGACATGTTCTCCCCTTCTTCCTCTCCCTCCTCTTCCCTCCTAAGTTTCGAGCTGGACTCAGTGCAGAGACTAGCCTCCAGTACCCACC
GTCTCCCGCCGCACAGTCTCTAATG TTACACTCGGACCTCGTCGACATGCTTTCCCCGAAGCTTCTTGCGTCCATCAGGCCTCCTAAGTTTCGAGCTGGACTCAGTGCAGAGACTAGCCTCCAGTACCCACC
