# Supplementary material for: Exon junction complex dependent mRNA localization is linked to centrosome organization during ciliogenesis
Source: Nat Commun. 2021 Mar 1;12:1351. doi: 10.1038/s41467-021-21590-w (PMC7921557; doi:10.1038/s41467-021-21590-w)
Supplement: Supplementary file 4 — Description of Additional Supplementary Files [file 41467_2021_21590_MOESM4_ESM.docx]

Description of Supplementary Information

Title: Supplementary DATA 1.

Description: High-throughput smFISH screen identifies transcripts displaying specific localization in quiescent RPE1 cell. Sequence reference ensemble access code and gene name of analyzed mRNAs are depicted with subcellular localization patterns.

Title: Supplementary DATA 2.

Description: Sequence of smFISH probes in high-throughput smFISH screen Sequences of smFISH probes for each transcript are depicted
