# Supplementary material for: Exon junction complex dependent mRNA localization is linked to centrosome organization during ciliogenesis
Source: Nat Commun. 2021 Mar 1;12:1351. doi: 10.1038/s41467-021-21590-w (PMC7921557; doi:10.1038/s41467-021-21590-w)
Supplement: Supplementary file 5 — Reporting Summary [file 41467_2021_21590_MOESM5_ESM.pdf]

nature research

Corresponding author(s): Hervé le Hir

Last updated by author(s): YYYY-MM-DD

Reporting Summary

Nature Research wishes to improve the reproducibility of the work that we publish. This form provides structure for consistency and transparency in reporting. For further information on Nature Research policies, see our Editorial Policies and the Editorial Policy Checklist.

Statistics

For all statistical analyses, confirm that the following items are present in the figure legend, table legend, main text, or Methods section.

n/a	Confirmed	
☐	☒	The exact sample size (*n*) for each experimental group/condition, given as a discrete number and unit of measurement
☐	☒	A statement on whether measurements were taken from distinct samples or whether the same sample was measured repeatedly
☐	☒	The statistical test(s) used AND whether they are one- or two-sided<br>*Only common tests should be described solely by name; describe more complex techniques in the Methods section.*
☒	☐	A description of all covariates tested
☒	☐	A description of any assumptions or corrections, such as tests of normality and adjustment for multiple comparisons
☐	☒	A full description of the statistical parameters including central tendency (e.g. means) or other basic estimates (e.g. regression coefficient) AND variation (e.g. standard deviation) or associated estimates of uncertainty (e.g. confidence intervals)
☐	☒	For null hypothesis testing, the test statistic (e.g. *F*, *t*, *r*) with confidence intervals, effect sizes, degrees of freedom and *P* value noted<br>*Give P values as exact values whenever suitable.*
☒	☐	For Bayesian analysis, information on the choice of priors and Markov chain Monte Carlo settings
☒	☐	For hierarchical and complex designs, identification of the appropriate level for tests and full reporting of outcomes
☐	☒	Estimates of effect sizes (e.g. Cohen's *d*, Pearson's *r*), indicating how they were calculated

Our web collection on statistics for biologists contains articles on many of the points above.

Software and code

Policy information about availability of computer code

Data collection	Images were acquired with an epifluorescence microscope (Nikon, ECLIPSE Ti) equipped with a plan APO VC 60 X objective (N.A 1.4, Nikon), CCD camera (Hamamatsu, ORCA Flash 4.0) and operated by Micro-Manager (MM studio, version 1.4.22). High throughput-smFISH-Immuno fluorescence images were imaged on Opera Pheonix High-content Screening system (PerkinElmer). Flow cytometry data were acquired by a ZE5 cell analyzer (Bio-Rad) using Everest acquisition software (Bio-Rad, Version 2.5). Quantitative RT-PCR data were acquired by CFX384 Real time system (Bio-Rad). Western blot images were acquired by ImageQuant LAS 4000 (Ge healthcare, version 1.3). Whole membrane stain was scanned by EPSON perfection V700 photo.
Data analysis	Fluorescence intensity and co-localization were analyzed by ImageJ (National Institute of Health, Bethesda, version 1.52d). Proportion of ciliated cell, detectable centrosomal eIF4A3 and Y14, and dispersed centrosome are analyzed base on the poly-glutamylated tubulin, eIF4A3, Y14, PCM1, and FOP staining and caluated by Excel 2016 (Microsoft). Cell cycle analysis was performed by acquiring HOECHST staining width and area by ZE5 cell analyzer (Bio-Rad) using Everest acquisition software (Version 2.5), and acquired data were analyzed using FlowJo software (FlowJo, LLC, version 10.6.0). Western blot band intensity was measured by ImageJ (National Institute of Health, Bethesda, version 1.51j8). Quantitative RT-PCR data were analyzed in Excel 2016 (Microsoft).

For manuscripts utilizing custom algorithms or software that are central to the research but not yet described in published literature, software must be made available to editors and reviewers. We strongly encourage code deposition in a community repository (e.g. GitHub). See the Nature Research guidelines for submitting code & software for further information.

Data

Policy information about availability of data

All manuscripts must include a data availability statement. This statement should provide the following information, where applicable:

- Accession codes, unique identifiers, or web links for publicly available datasets
- A list of figures that have associated raw data
- A description of any restrictions on data availability

The authors confirm that all relevant data are contained in this published article and supplementary files. Additional raw data are available upon request.

Field-specific reporting

Please select the one below that is the best fit for your research. If you are not sure, read the appropriate sections before making your selection.

☒ Life sciences ☐ Behavioural & social sciences ☐ Ecological, evolutionary & environmental sciences

For a reference copy of the document with all sections, see nature.com/documents/nr-reporting-summary-flat.pdf

Life sciences study design

All studies must disclose on these points even when the disclosure is negative.

Sample size	All the fluorescence analyses were performed with more than 60 cells from three independent experiments and flow cytometry was performed with more than 3X10000 cells per experiment in four biological replicates to ensure reproducibility and to get statistically significant results.
Data exclusions	No data were excluded from the analysis.
Replication	Each data presented in this work was repeated in multiple times and/or across multiple cells and/or animals. The precise number of repeats are given in the figure legend.
Randomization	Because, all experiments are based on the cell cultivation system, groups are determined by transfection and chemical treatment from the same batches of RPE1 and RPE1 centrin1-GFP cells. Therefore, no further randomization strategy was applicable to our research.
Blinding	Since our study is based on cell culture system, blinding was not relevent to our study. Data are not subjective but rather based on quantitative analysis.

Reporting for specific materials, systems and methods

We require information from authors about some types of materials, experimental systems and methods used in many studies. Here, indicate whether each material, system or method listed is relevant to your study. If you are not sure if a list item applies to your research, read the appropriate section before selecting a response.

Materials & experimental systems

n/a	Involved in the study	
☐	☒	Antibodies
☐	☒	Eukaryotic cell lines
☒	☐	Palaeontology and archaeology
☐	☒	Animals and other organisms
☒	☐	Human research participants
☒	☐	Clinical data
☒	☐	Dual use research of concern

Methods

n/a	Involved in the study	
☒	☐	ChIP-seq
☐	☒	Flow cytometry
☒	☐	MRI-based neuroimaging

Antibodies

Antibodies used	Provider, clone number, catalog number, lot number, and host are depicted in brackets as applicable. For Western blot and RIP: anti-Puromycin (Merck, 12D10, MABE343, 2923223, mouse), anti-GAPDH (cell signaling, 14C10, 2118S, 14, Rabbit), anti-eIF4A3 (affinity purified from rabbit serum), anti-Y14 (affinity purified from rabbit serum), anti-MAGOH (Santacruz, 21B12, sc-56724, C2013, mouse), anti-MLN51 (affinity purified from rabbit serum), anti-Rab5 (affinity purified from rabbit serum), anti-mouse antibody conjugated with HRP (Bethyl, A90-116P, S90-116P-34), anti-Rabbit antibody conjugated with HRP (Promega, W401B, 0000390794, Goat). For immunofluorescence: anti-FOP (Abnova, 2B1, H00011116-M01, I1291-2B1, Mouse), anti-Polyglutamylated tubulin (ADIPOGEN, GT-335, AG-20B-0020, A27791601, Mouse), anti-Y14 (Santacruz, 4C4, sc-32312, H2416, Mouse), anti-eIF4A3 (affinity purified from rabbit serum), anti-alpha-tubulin (Sigma, DM1A, T6199, 11M4849, Mouse), anti-beta tubulin (Biolegend, TU27, 903401, B203934, Mouse), anti-gamma-tubulin (Sigma, GTU-88, T6557, 034M4794V, Mouse), anti-EDC4 (Santacruz, SC-8416, Mouse), anti-DDX6

(Bethyl, BL2142, A300-461A, Rabbit), anti-TIA-1 (Santacruz, C-20, SC-1751, Goat), anti-eIF4E (Santacruz, FL217, SC-13963, D1112, Rabbit), anti-GM130 (BD bioscience, 35GM130, BD610822, 6217559 Mouse), anti-Pericentrin (Covance, PRB-432C, E11HF01670, Rabbit), anti-PCM1(Cell signaling, G2000, 5213S, 1, Rabbit), anti-9G8 (Received from James stevenin's lab in IGBMC, Rabbit), anti-SC35 (Received from James stevenin's lab in IGBMC, Mouse), anti-NIN (Institut curie, 2G5, Human), anti-Arl13b (proteintech, 17711-1-AP, Rabbit), anti-Goat DyLight650 (Bethyl, A50-201D5, Donkey), anti-Human Alexa 488 (Invitrogen, A11013, 1173476, Goat), anti-Rabbit Alexa594 (ThermoFisher, A21207, 1107500, Donkey), anti-Mouse IgG1 Alexa594 (Invitrogen, A21203, 645165, Donkey), anti-Mouse IgG2b Alexa488 (ThermoFisher, A21141, 2128994, Goat), anti-Mouse IgG1 Alexa647 (ThermoFisher, A21240, 2092265, Goat), anti-Mouse Alexa488 (Thermofischer, A-11001,1939600, Goat), anti-Rabbit Cy5 (Jackson ImmunoResearch, 111-176-047, 57833, Goat).

Validation

1. Anti-Puromycin (Merck, 12D10, MABE343)
Species and application: All species, applied for FACS, immunofluorescence, western blot, IP, immuno histochemistry.
Evaluated by Western Blotting in HEK293 cell lysates treated with Puromycin and Cyclohexamide, or with Puromycin only.
Western Blotting Analysis: A 1:25,000 dilution of this antibody detected Puromycin-incorporated neosynthesized proteins in HEK293 cell lysates treated with Puromycin only. This antibody also detected small amounts of Puromycin-incorporated neosynthesized proteins in HEK293 cells treated with Puromycin and Cyclohexamide.
References
Reineke, L. C., et al. (2012). Mol Biol Cell. 23(18):3499-3510.
Trinh, M. A. et al. (2012). Cell Rep. 1(6):678-688.
Fortin, D. A., et al. (2012). J Neurosci. 32(24):8127-8137
Reineke, L. C., et al. (2012). Mol Biol Cell. 23(18):3499-3510.
Trinh, M. A. et al. (2012). Cell Rep. 1(6):678-688

2. Anti-GAPDH (cell signaling, 14C10, 2118S)
Species and application: Human, mouse, rat, monkey, bovine, pig, immunofluorescence, western blot, immuno histochemistry, FACS.
Monoclonal antibody is produced by immunizing animals with a synthetic peptide near the carboxy terminus of human GAPDH.
Western blot analysis of extracts from various cell lines using GAPDH (14C10) Rabbit mAb detected endogenous GAPDH. Western Blotting 1:1000, Immunohistochemistry (Paraffin) 1:800, Immunofluorescence (Immunocytochemistry) 1:100, Flow Cytometry 1:200
References
Western Blotting Analysis: Boteva et al. Cell Rep. 2020 Sep 22;32(12):108177
Chiba et al. Redox Biol. 2020 Sep 14;37:101720.

3. Anti -MAGOH (Santacruz, 21B12, sc-56724)

Species and application: mouse, rat and human, immunofluorescence, western blot, IP, immuno histochemistry.
Anti-MAGOH Antibody (21B12) is a mouse monoclonal IgG2a κ MAGOH antibody and raised against full length MAGOH of human origin. MAGOH (21B12) is recommended for detection of MAGOH of mouse, rat and human origin by Western Blotting (starting dilution 1:200, dilution range 1:100-1:1000), immunoprecipitation [1-2 µg per 100-500 µg of total protein (1 ml of cell lysate)], immunofluorescence (starting dilution 1:50, dilution range 1:50-1:500) and immunohistochemistry (including paraffin-embedded sections) (starting dilution 1:50, dilution range 1:50-1:500).
References
Li et al. Nat Microbiol. 2019 Jun;4(6):985-995.
Geißler et al. Nucleic Acids Res. 2013 Sep;41(16):7875-88.

4. Anti -FOP (Abnova, 2B1, H00011116-M01)

Species and application: Human, immunofluorescence, western blot, ELISA
Mouse monoclonal antibody raised against a full-length recombinant FGFR1OP. FGFR1OP (AAH11902, 1 a.a. ~ 379 a.a) full-length recombinant protein with GST tag. MW of the GST tag alone is 26 KDa. immunofluorescence: 10 ug/ml, ELISA: 0.03ng/ml

references
Chouaib et al. Dev Cell. 2020 Sep 28;54(6):773-791.e5.
Mahuzier et al. N.Nat Commun. 2018 Jun 11;9(1):2279.

5. Anti-Polyglutamylated tubulin (ADIPOGEN, GT-335, AG-20B-0020)

Species and application: all, electron microscopy, immunocytochemistry, immunohistochemistry, immunoprecipitation, western blot
Purified from concentrated hybridoma tissue culture supernatant. Octapeptide EGEGE*EEG, modified by the addition of two glutamyl units onto the fifth E (indicated by an asterisk). Recognizes the posttranslational modification (poly)glutamylation on proteins. Reacts with polyglutamylated α- and β-tubulin. Immunocytochemistry: (1:2000), Immunohistochemistry: (paraffin sections; 1:1000), Western Blot: (1:4000)
References
A. Wolff, et al.; Eur. J. Cell Biol. 59, 425 (1992)
C. Regnard, et al.; J. Biol. Chem. 275, 15969 (2000)
M.L. Kann, et al.; Cell Motil. Cytoskeleton 55, 14 (2003)

6. Anti-Y14 (Santacruz, 4C4, sc-32312)
Species and application: human, Xenopus laevis, and RBM8 of mouse and rat, western blot, IP, immunofluorescence, immunohistochemistry, and ELISA
Anti-Y14 Antibody (4C4) is a mouse monoclonal IgG2b κ Y14 antibody and raised against native full length human Y14. Y14 (4C4) is recommended for detection of Y14 of human and Xenopus laevis origin, and RBM8 of mouse and rat origin by Western Blotting (starting dilution 1:200, dilution range 1:100-1:1000), immunoprecipitation [1-2 µg per 100-500 µg of total protein (1 ml of cell lysate)], immunofluorescence (starting dilution 1:50, dilution range 1:50-1:500), immunohistochemistry (including paraffin-embedded sections) (starting dilution 1:50, dilution range 1:50-1:500) and solid phase ELISA (starting dilution 1:30, dilution range 1:30-1:3000).
References

Blazquez, L. et al. 2018. Mol. Cell. 72: 496-509.e9.
Ni, X. et al. 2018. Oncol. Rep. 39: 13-20.
Fehler, O. et al. 2014. Nucleic acids research. 42: 11107-18.

7. Anti-alpha-tubulin (Sigma, DM1A, T6199)
Species and application: human, bovine, mouse, and chicken, western blot, solid-phase RIA,1 cell antibody microinjection, immunocytochemistry, immunohistochemistry, and immunoprecipitation.
Anti-a-Tubulin antibody, Mouse monoclonal (mouse IgG1 isotype) is derived from the hybridoma DM1A produced by the fusion of mouse myeloma cells (NS1) and splenocytes from BALB/c mice immunized with purified chick brain tubulin. The isotype is determined by a double diffusion immunoassay using Mouse Monoclonal Antibody Isotyping Reagents, Product Number ISO2. Anti-a-Tubulin antibody, Mouse monoclonal recognizes an epitope located at the C-terminal end of the a-tubulin isoform (amino acids 426-430). Immunoblotting: a working antibody concentration of 0.5-1 mg/ml is recommended using a total tissue extract from chicken gizzard. Immunocytochemistry: a working antibody concentration of 0.5-1 mg/ml is recommended using cultured chicken fibroblasts (CFB).
References
Blose, S.H., et al., J. Cell Biol., 98, 847-858 (1984).
Breitling, F., and Little, M., J. Molec. Biol., 189, 367- 370 (1986).
Machaca, K. and Haun, S., et al., J. Biol. Chem., 49, 38710-38715 (2000).

8. Anti-beta-tubulin (Biolegend, TU27, 903401)
Species and application: Bovine, Rat, Chinese Hamster, Sea Urchin, Chlamydamonas, Drosophila, western blot, immunofluorescence, immunohistochemistry.
The TU27 cloneis broadly reactive to b-tubulin subtypes and binds outside of the C-terminal isotype domains, but does not crossreact with alpha-tubulin by immunoblotting. It recognizes a highly conserved epitope and reacts with ß-tubulin found in vertebrate as well as in vertebrate tissues. Reactivity to Drosophila was onlyverified with the purified format. This antibody iseffectivein immunoblotting and immunostaining. WB: 1 µg/ml (1:1000), IF: 1,000.
References
MK Lee et al. 1990. CellMotil. Cytoskel. 17:118
JE Alexander et al. 1991. Proc Natl Acad Sci USA. 88:4685
J Lisztwan et al. 1998. EMBO J. 17:368

9. Anti-gamma-tubulin (Sigma, GTU-88, T6557)
Species and application: human, bovine, dog, hamster, rat, mouse, chicken, and Xenopus, western blot, IP, immunofluorescence, ELISA
Monoclonal Anti-g-Tubulin (mouse IgG1 isotype) is derived from the GTU-88 hybridoma produced by the fusion of mouse myeloma cells and splenocytes from an immunized mouse. A synthetic g-tubulin peptide (N-terminal amino acids 38-53) conjugated to KLH was used as the immunogen. The isotype is determined by a double diffusion immunoassay using Mouse Monoclonal Antibody Isotyping Reagents, Catalog Number ISO2. Monoclonal Anti-g-Tubulin recognizes an epitope located in the N-terminal amino acids 38-53 of g-tubulin (48 kDa). Immunoblotting: a minimum working antibody dilution of 1:10,000 is determined using cultured chicken fibroblasts extract. Immunocytochemistry: a minimum working antibody dilution of 1:5,000 is determined using HeLa cells.
References
Oakley, B et al. Trends Cell Biol., 2, 1 (1992)
Stebbings, H et al. Nature, 336, 14 (1988)
Oakley, C et al. and Oakley, B., Nature, 338, 662 (1989)

10. Anti-EDC4 (Santacruz, A-6, SC-8418)
Species and application: mouse, rat and human, western blot, IP, immunofluorescence, immunohistochemistry, ELISA
The information of this antibody in web site shows different one. The validation performed in reference reports. p70 S6 kinase α (H-9) is a mouse monoclonal antibody specific for an epitope mapping between amino acids 429-441 at the C-terminus of p70 S6 kinase α of rat origin. Western Blotting (starting dilution 1:200, dilution range 1:100-1:1000), immunoprecipitation [1-2 µg per 100-500 µg of total protein (1 ml of cell lysate)], immunofluorescence (starting dilution 1:50, dilution range 1:50-1:500), immunohistochemistry (including paraffin embedded sections) (starting dilution 1:50, dilution range 1:50-1:500), flow cytometry (1 µg per 1 x 106 cells) and solid phase ELISA (starting dilution 1:30, dilution range 1:30-1:3000).
Reference
Ayache et al. Mol Biol Cell. 2015 Jul 15; 26(14): 2579–2595.
Hubstenberger et al. Mol cell, Volume 68, Issue 1, 5 October 2017, Pages 144-157.e5
Navarro et al. Nature Communications volume 10, Article number: 3230 (2019)

11. Anti-DDX6 (Bethyl, BL2142, A300-461A)
Species and application: Human, Mouse, western blot, IP, immunohistochemistry.
The epitope recognized by A300-461A maps to a region between residues 425 and 483 of human DEAD (Asp-Glu-Ala-Asp) box polypeptide 6 using the numbering given Swiss-Prot entry NP_004388.2 (GeneID 1656). Western Blot 1:25,000 - 1:50000, Immunoprecipitation 2 - 10 µg/mg lysate, Immunohistochemistry 1:1000 to 1:5000.
Reference
Wilbertz et al., 2019, Molecular Cell 73, 946–958
Berchtold et al., 2018, Molecular Cell 72, 1035–1049

12. Anti-TIA-1 (Santacruz, C-20, SC-1751)
Species and application: human, mouse, rat, mouse, rat, human, western blot, IP, immunofluorescence, ELISA
TIA-1 Antibody (C-20) is a goat polyclonal IgG; 100 µg/ml, epitope mapping near the C-terminus of TIA-1 of human origin. TIA-1 (C-20) is recommended for detection of TIA-1 of mouse, rat and human origin by Western Blotting (starting dilution 1:200, dilution range 1:100- 1:1000), immunoprecipitation [1-2 µg per 100-500 µg of total protein (1 ml of cell lysate)], immunofluorescence (starting dilution 1:50, dilution range 1:50- 1:500), immunohistochemistry (including paraffin-embedded sections) (starting dilution 1:50, dilution range 1:50-1:500) and solid phase ELISA (starting dilution 1:30, dilution range 1:30-1:3000).
Reference
Del Gatto-Konczak, F., et al. 2000. Mol. Cell. Biol. 20: 6287-6299

Subramaniam, K., et al. 2010. Cancer Lett. 297: 259-268
Sola, I., et al. 2011. J. Virol. 85: 5136-5149

13. Anti-eIF4E (Santacruz, FL217, SC-13963)
Species and application: mouse, rat, human, western blot, IP, immunofluorescence, immunohistochemistry, ELISA
eIF4E (FL-217) is recommended for detection of eIF4E of mouse, rat and human origin by Western Blotting (starting dilution 1:200, dilution range 1:100-1:1000), immunoprecipitation [1-2 μg per 100-500 μg of total protein (1 ml of cell lysate)], immunofluorescence (starting dilution 1:50, dilution range 1:50-1:500), immunohistochemistry (including paraffin-embedded sections) (starting dilution 1:50, dilution range 1:50-1:500) and solid phase ELISA (starting dilution 1:30, dilution range 1:30-1:3000).
Reference
Iresjö, B., et al. 2008. Clin. Sci. 114: 603-610
Schweitzer, C.J., et al. 2012. PLoS ONE 7: e40537
Rozelle, D.K., et al. 2014. Mol. Cell. Biol. 34: 2003-2016

14. Anti-GM130 (BD bioscience, 35GM130, BD610822)
Species and application: Human, Mouse, western blot, immunohistochemistry, ICC, ELISA
The epitope recognized by A300-461A maps to a region between residues 425 and 483 of human DEAD (Asp-Glu-Ala-Asp) box polypeptide 6 using the numbering given Swiss-Prot entry NP_004388.2 (GeneID 1656). Western Blot 1:25000 - 1:50000 Immunoprecipitation 2 - 10 μg/mg lysate Immunohistochemistry 1:1000 to 1:5,000.
Reference
von Gamm, M., Schaub, A., et al. The Journal of Experimental Medicine on 1 July 2019
Wilbertz, J. H., Voigt, F., et al. Molecular Cell on 7 March 2019

15. Anti-Pericentrin (Covance, PRB-432C)
Species and application: Vertebrate, Insect, Ciliate, Microorganisms Flagellate, immunoblotting, immunofluorescence, immunoprecipitation, and immunogold electron microscopy.
This antibody was generated against a fusion protein containing ~60kD of pericentrin. Each lot of this antibody is quality control tested by immunocytochemistry. The optimal working dilution should be determined for each specific assay condition. WB: 1:300, IF: 1:500
References
Connell M et al. HMMR acts in the PLK1-dependent spindle positioning pathway and supports neural development. elife. 2017;6
Kim M et al. Methods Cell Biol. 2016;136:269-83

16. Anti-PCM1(Cell signaling, G2000, 5213S)
Species and application: Human, Mouse, immunofluorescence, immunoprecipitation, western Blot, Flow cytometry
PCM-1 (G2000) Antibody detects endogenous levels of PCM-1 protein. Species cross-reactivity for IF-IC is human only. Western Blotting 1:1000, Immunoprecipitation 1:100, Immunofluorescence 1:800, Flow Cytometry 1:100.
References
Zimmerman, W. and Doxsey, S.J. (2000) Traffic 1, 927-34
Balczon, R. et al. (1994) J Cell Biol 124, 783-93

17. Anti-Arl13b (proteintech, 17711-1-AP)
human, mouse, rat, dog, immunofluorescence, immunohistochemistry, immunoprecipitation, western blot, ELISA
This antibody detectstwo specific bands at 60 kDa and 48 kDa. Arl13b is predicted to be a 48 kDa protein, and the 60 kDa band islikely to represent a modified form of Arl13b. ARL13B can be used to mark the cilia (PMID:22072986). WB 1:500-1:2000, IP 0.5-4.0 ug for IP, IHC 1:50-1:500, IF 1:50-1:500
References
Capowski et al. Development. 2019 Jan 9;146(1):dev171686.
Lee et al. Mol Cells. 2019 Feb 28;42(2):113-122.

Antibodies against following proteins used for immunofluorescence, western blot, and RIP are previously described and validated. eIF4A3, Y14, MLN51, Rab5 antibodies were previously described and validated in previous reports (Wang et al. Genome Biology, volume 15, Article number: 551 (2014), Saulière et al. Nature Structural & Molecular Biology, volume 19, pages1124–1131(2012)). eIF4A3, Y14, MLN51 antibodies showed specific bands at expected size and down-regulation by corresponding siRNAs. RAB5 antibody was used as a negative control that are not associated with EJC in previous and this reports. Antibody against eIF4A3 showed specific down-regulated fluorescence intensity upon siRNA treatment in this report.
Antibodies against SC35 and 9G8 was previously described and validated (Daguenet et al. Mol Biol Cell. 2012 May;23(9):1765-82, CAVALOC et al. RNA (1999), 5:468–483). Immunoprecipitation shows SC35 and 9G8 antibodies recognize exclusively their corresponding proteins, and immunofluorescence signal shows clear nuclear speckle pattern.
Anti NIN antibody was previously described and validated (Bouckson-Castaing et al. Journal of Cell Science 1996 109: 179-190). Immunoprecipitation with NIN antibody specifically capture NIN protein. Immunofluorescence and immunoelectron microscopic studies showed specific localization of NIN protein at centrosome.

Eukaryotic cell lines

Policy information about cell lines

Cell line source(s)

RPE1 cells, a human retinal pigment epithelial cell line that stably expresses human telomerase reverse transcriptase (hTERT-RPE1; Clontech Laboratories, Inc.). RPE1 cen-GFP was kind gift from Benedicte Delaval and described in previous report (Mikule et al. Nature Cell Biology volume 9, pages160–170(2007)).

Authentication

RPE1 cells were genotyped by Eurofins. Since RPE1 cen-GFP was reported previously (Mikule et al. Nature Cell Biology volume 9, pages160–170(2007)), we did not authenticate.

Mycoplasma contamination	All cell lines used in this study were routinely checked for mycoplasma by contamination and had no contamination.
Commonly misidentified lines (See ICLAC register)	No commonly identified cell line was used.

Animals and other organisms

Policy information about studies involving animals; ARRIVE guidelines recommended for reporting animal research

Laboratory animals	The mice used in this study have already been described and include: RjOrl:SWISS (Janvier Laboratories). Both males and females were used in this study. Mice were grown in individual ventilated cages. Temperature and humidity were maintained in 20-21? and in 50-55%, respectively. Light cycle was 7 AM-7PM.
Wild animals	No wild animals were used in the study.
Field-collected samples	No field collected samples were used in the study.
Ethics oversight	All animal studies in this report were performed in accordance with the guidelines of the European Community and French Ministry of Agriculture and were approved by the Direction départementale de la protection des populations de Paris and ISERM (Approval number APAFIS#9343-201702211706561 v7).

Note that full information on the approval of the study protocol must also be provided in the manuscript.

Flow Cytometry

Plots

Confirm that:

☒ The axis labels state the marker and fluorochrome used (e.g. CD4-FITC).

☒ The axis scales are clearly visible. Include numbers along axes only for bottom left plot of group (a 'group' is an analysis of identical markers).

☒ All plots are contour plots with outliers or pseudocolor plots.

☒ A numerical value for number of cells or percentage (with statistics) is provided.

Methodology

Sample preparation	Cells were trypsinized and resuspended with DPBS. Resuspended cells were permeabilized by incubating with extraction buffer (0.2 % Triton X-100 in PBS) for 5 min in ice, fixed by incubating with fixation buffer (2 % PFA in PBS) for 15 min in RT and stored in storage buffer (3 % FBS, 0.09 % sodium azide in DPBS). Cells were labeled by 10 microgram/ml of HOECHST 33258 for 30 min in RT.
Instrument	ZE5 cell analyzer (Bio Rad)
Software	Samples were analyzed on a ZE5 cell analyzer (Bio-Rad) using Everest acquisition software (Version 2.5). Data were analyzed using FlowJo software (FlowJo, LLC, version 10.6.0).
Cell population abundance	We used only RPE1 cells in this research.
Gating strategy	Cells were selected based on their morphology (SSC versus FSC: "Cells" population) then doublets of cells were excluded based on Hoechst fluorescent parameters (Hoechst-A versus Hoechst-W: "Single cells" population). Cell cycle analysis was performed on single cell population using FlowJo module for cell cycle.

☒ Tick this box to confirm that a figure exemplifying the gating strategy is provided in the Supplementary Information.
